# Origins of Ultrasensitivity and Complex Signaling Dynamics of Cellular Hydrogen Peroxide and Peroxiredoxin

**DOI:** 10.3390/antiox14020235

**Published:** 2025-02-18

**Authors:** Shengnan Liu, Jingbo Pi, Qiang Zhang

**Affiliations:** 1 Key Laboratory of Environmental Stress and Chronic Disease Control & Prevention Ministry of Education, China Medical University, Shenyang 110122, China; 2 Key Laboratory of Liaoning Province on Toxic and Biological Effects of Arsenic, China Medical University, Shenyang 110122, China; 3 Program of Environmental Toxicology, School of Public Health, China Medical University, Shenyang 110122, China; 4 Gangarosa Department of Environmental Health, Rollins School of Public Health, Emory University, Atlanta, GA 30322, USA

**Keywords:** H_2_O_2_, peroxiredoxin, ultrasensitivity, bistability, feedback, circadian rhythm

## Abstract

Hydrogen peroxide (H_2_O_2_) plays a crucial role in cell signaling in response to physiological and environmental perturbations. H_2_O_2_ can oxidize typical 2-Cys peroxiredoxin (PRX) first into a sulfenic acid, which resolves into a disulfide that can be reduced by thioredoxin (TRX)/TRX reductase (TR). At high levels, H_2_O_2_ can also hyperoxidize sulfenylated PRX into a sulfinic acid that can be reduced by sulfiredoxin (SRX). Therefore, PRX, TRX, TR, and SRX (abbreviated as PTRS system here) constitute the coupled sulfenylation and sulfinylation cycle (CSSC), where certain oxidized PRX and TRX forms also function as redox signaling intermediates. Earlier studies have revealed that the PTRS system is capable of rich signaling dynamics, including linearity, ultrasensitivity/switch-like response, nonmonotonicity, circadian oscillation, and possibly, bistability. However, the origins of ultrasensitivity, which is fundamentally required for redox signal amplification, have not been adequately characterized, and their roles in enabling complex nonlinear dynamics of the PTRS system remain to be determined. Through in-depth mathematical modeling analyses, here we revealed multiple sources of ultrasensitivity that are intrinsic to the CSSC, including zero-order kinetic cycles, multistep H_2_O_2_ signaling, and a mechanism arising from diminished H_2_O_2_ removal at high PRX hyperoxidation state. The CSSC, structurally a positive feedback loop, is capable of bistability under certain parameter conditions, which requires embedding multiple sources of ultrasensitivity identified. Forming a negative feedback loop with cytosolic SRX as previously observed in energetically active cells, the mitochondrial PTRS system (where PRX3 is expressed) can produce sustained circadian oscillations through supercritical Hopf bifurcations. In conclusion, our study provided novel quantitative insights into the dynamical complexity of the PTRS system and improved appreciation of intracellular redox signaling.

## 1. Introduction

### 1.1. H_2_O_2_ as a Redox Signaling Molecule

Although reactive oxygen species (ROS) are generally cytotoxic in aerobic cells [1], their role as redox signaling molecules, especially hydrogen peroxide (H_2_O_2_), has also been well established [2,3]. H_2_O_2_ mediates diverse physiological responses including cell proliferation, differentiation, death, migration, hormone secretion, and circadian rhythm [4,5,6,7,8,9,10]. Dysregulation of redox signaling is associated with numerous disease conditions [11]. Signaling H_2_O_2_ can be generated by several endogenous sources. For instance, when cells are stimulated by a variety of growth factors, H_2_O_2_ can derive from NADPH oxidase (NOX) residing on the plasma membrane or the endomembrane of the endoplasmic reticulum (ER) and endosome [12,13,14]. H_2_O_2_ so produced can act as a second messenger transducing redox signals to downstream molecules such as protein tyrosine phosphatases (PTP) [5,15]. Another source of signaling H_2_O_2_ is the mitochondrion. We and others have demonstrated that H_2_O_2_ derived from glucose oxidative metabolism in the mitochondrion serves as an obligatory metabolic signal to mediate glucose-stimulated insulin secretion in pancreatic β-cells [9,16] and glucose-sensing in the hypothalamus [17].

### 1.2. Complex H_2_O_2_ Signaling Dynamics

Molecular signaling in cells is complex, often exhibiting nonlinear dynamics, including sigmoidal responses for signal amplification, switch-like responses for binary decision-making, and oscillations for biological rhythms [18]. As a matter of fact, cell functions cannot be sustained if relying on linear signaling alone [19]. The redox system is no exception. A number of experimental studies have demonstrated that cellular H_2_O_2_ can exhibit biphasic responses or bimodal distributions, supporting the notion of threshold or switch-like behaviors of H_2_O_2_. For instance, in both fission yeast and mammalian HEK298 cells, intracellular H_2_O_2_ exhibited biphasic dose responses to increasing extracellular H_2_O_2_ concentrations [20]. Bimodal intracellular H_2_O_2_ distributions were observed in mammalian cells, e.g., in umbilical cord mononuclear blood cells from tobacco smokers [21] and in bone marrow mononuclear cells of aged C57BL/6 mice [22]. In T98 cells, a glucose-addicted cell line, ROS, particularly H_2_O_2_, can be toggled between two distinct states with hysteresis by controlling the availability of glucose [23]. ROS signal amplification and self-perpetuation have also been suggested for the YAP-HIF-Notch-(PD-L1) signaling axis, which may underpin decision-making in development, tissue homeostasis, and cancer progression [24]. Feedback loops containing growth factor receptors, PTP, peroxiredoxin, and Src are believed to amplify locally NOX-generated H_2_O_2_ into hotspots near the cell membrane [25,26,27,28,29]. Many theoretical studies through mathematical modeling have shown that, depending on the cellular context of the peroxiredoxin system, H_2_O_2_ can exhibit switching behaviors with spatial complexities [20,30,31,32,33,34,35,36,37]. Last but not least, H_2_O_2_ circadian or ultradian oscillations have also been observed in fish, yeast, and mammals, which play a crucial role in redox timekeeping [10,38,39,40].

### 1.3. Nonlinear Dynamics of the Peroxiredoxin (PRX), Thioredoxin (TRX), TRX Reductase (TR), and Sulfiredoxin (SRX) System (PTRS)

**Figure 1:**
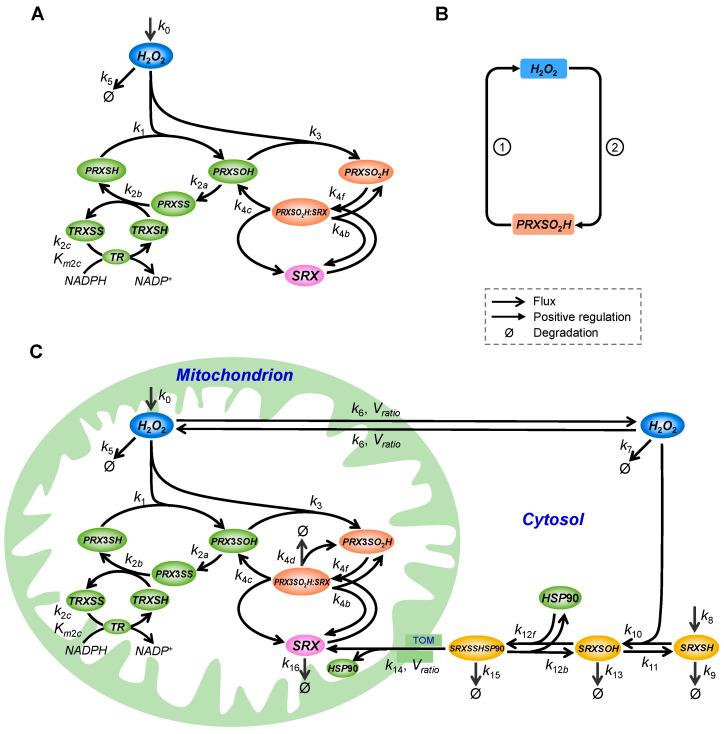
**Model structures of the PTRS systems.** (**A**) The reaction scheme of the modeled CSSC of 2-Cys PRX. (**B**) The positive feedback loop between *H*_2_*O*_2_ and *PRXSO*_2_*H* hidden in (**A**). ① and ② indicate the two arms. (**C**) The reaction scheme of the modeled *SRX*-mediated negative feedback for circadian oscillation of *H*_2_*O*_2_ and *PRX3*.

The rich H_2_O_2_ dynamics can be underpinned by a variety of complex molecular interactions and circuitries that regulate its production and metabolism [8,23,24,25,26,27,28,29]. Among them, a key metabolic pathway is through the PTRS system. As the most abundant antioxidants with high second-order rate constants, PRX is responsible for eliminating most, as high as 90%, of cellular H_2_O_2_ produced [31,41], while the rest is degraded by glutathione peroxidase (GPx) and catalase (CAT). There are six mammalian PRX isoforms according to their subcellular locations. PRX1, PRX2, and PRX6 are mainly in the cytoplasm, PRX3 in the mitochondrion, PRX4 in the ER, and PRX5 in different cellular compartments including peroxisome and mitochondrion [42]. Although PRX often exists as decamers (PRX1/2) or dodecamers (PRX3), the function unit of a typical 2-Cys PRX molecule is a homodimer, with each monomer containing a conserved peroxidative cysteine (C_P_) residue and a resolving cysteine (C_R_) residue. During the peroxidation cycle, C_P_ in the reduced form of PRX (PRXSH) is oxidized by H_2_O_2_ into C_P_-SOH to form a sulfenic acid PRXSOH (Figure 1A). Reduction in PRXSOH involves a two-step process, where C_P_-SOH first spontaneously reacts with C_R_ in the partner PRX monomer of the homodimer to form an intra-molecular disulfide bond (PRXSS); the disulfide bond is then reduced back to PRXSH by TRX, and the resulting oxidized TRX (TRXSS) is reduced by TR [43]. PRXSOH can also be hyperoxidized, especially under high oxidative stress conditions, by H_2_O_2_ into a sulfinic acid (PRXSO_2_H), which is inactive as a peroxidase and can be reduced by SRX back to PRXSOH [44]. It is worth noting that at physiological pH the sulfenic and sulfinic acids of PRX are expected to be deprotonated; the abbreviations PRXSOH and PRXSO_2_H, which represent the protonated forms of the acids, are used here for typographical convenience.

The PTRS system is capable of nonlinear signaling dynamics. For instance, it was demonstrated experimentally in Jurkat cells that sulfinylated PRX1 and 2 exhibit all-or-none responses when exogenously added H_2_O_2_ concentrations doubled from 10 to 20 µM [45]. Similar albeit less steep responses in PRX1 and 2 sulfinylation were also observed in HeLa cells in the same study. Such nonlinear responses are also corroborated by many mathematical modeling studies. Models of the PRX2 system in red blood cells (RBC) and PRX3 system in HeLa cells showed highly switch-like H_2_O_2_ and PRXSO_2_H responses to increasing H_2_O_2_ production [36,37]. Salvador and colleagues et al. conducted further analyses, showing that different types of cells can in principle respond to increased H_2_O_2_ production in qualitatively distinct manners with spatial complexities, depending on the abundance of PRX, TRX, and TR and associated kinetic parameters [30,31,32]. These diverse responses range from proportional, saturable, sigmoidal, and nonmonotonic, to, in some cases, bistability (Bistability is the capability of a system staying in one of two possible stable steady states under the same condition and often exhibits a hysteretic response when the condition is varied). These complex dynamics are not limited to H_2_O_2_ and PRXSO_2_H, but are also exhibited by intermediate species such as PRXSOH, PRXSS, and TRXSS, which are believed to play crucial roles in relaying redox signaling initiated by H_2_O_2_ to target proteins [46,47,48,49,50,51,52].

In addition to enzymatic kinetics, a key contributing factor to these nonlinear dynamic behaviors of the PTRS system is its topology. Structurally, the coupled sulfenylation and sulfinylation cycle (CSSC) forms a double-negative feedback loop (DNFL) or positive feedback loop (PFL), which can be in disguise at first glimpse. Since PRXSH (the fully reduced form) participates in the sulfenylation reaction to remove H_2_O_2_ directly, and PRXSOH and PRXSS participate in the sequential de-sulfenylation reactions to recover depleted PRXSH, these three non-sulfinylated PRX species thus work collectively to reduce the amount of H_2_O_2_, hence logically speaking, “inhibiting” H_2_O_2_ levels. By promoting the sulfinylation of PRXSOH, H_2_O_2_ reduces the total amount of non-sulfinylated PRX but increases PRXSO_2_H, which is inactive. As a result, a DNFL arises between H_2_O_2_ and non-sulfinylated PRX. Equivalently, the system can be viewed as a PFL between H_2_O_2_ and PRXSO_2_H (Figure 1B), where H_2_O_2_ promotes PRXSO_2_H production as one arm and PRXSO_2_H increases H_2_O_2_ as the other arm through decreasing non-sulfinylated PRX (due to mass conservation of PRX). When the parameter condition is strongly favored for the operation of the PFL, the CSSC can produce highly switch-like and nonmonotonic responses [31].

Besides the steady-state response modality, PRX and H_2_O_2_ also exhibit circadian rhythms. The redox circadian clock involving PRX is believed to be evolutionarily more ancient [38,53], compared with the master circadian clock involving BMAL-CLOCK in eukaryotic cells [54,55]. Although the fraction of hyperoxidized PRX involved is uncertain, circadian hyperoxidation of different isoforms of PRX has been observed in a variety of species, spanning the spectrum of bacteria, archaea, and mammals [56,57]. These redox circadian oscillations do not appear to rely on a transcription–translation feedback loop (TTFL) circuit as exemplified by the BMAL-CLOCK master clock, since PRX also oscillates in anucleated RBC, which involves PRX2, and in eukaryotes where the transcriptional machinery was inhibited [58,59,60,61,62]. While the circadian oscillations of different PRX isoforms may operate via different molecular mechanisms in different cell types, the mammalian PRX3 circadian oscillation, observed for the sulfinylated form in mouse adrenal gland, brown adipose tissue, and heart, was believed to be mediated by a posttranslational trans-mitochondrial NFL embedding the P3TRS system (Figure 1C). It involves mitochondrial H_2_O_2_ release into the cytosol, oxidation of cytosolic SRX, translocation of oxidized SRX to the mitochondrion, and reduction of PRX3SO_2_H by SRX [7,8,57].

### 1.4. Objectives

Given the rich nonlinear dynamics exhibited by H_2_O_2_ and PRX, it is crucially important to understand the quantitative aspects of the PTRS system and the molecular and cellular conditions that are conducive to these dynamical behaviors. Although many mathematical modeling studies as above mentioned have analyzed the complexity of the PRX1, 2, and 3 systems in detail [30,31,32,36,37,63], the molecular reactions and associated kinetic parameter conditions that render these behaviors are still incompletely understood. In particular, the mechanistic origins of signal amplification, i.e., ultrasensitivity, in the CSSC have not been fully identified and characterized, despite its critical roles for PRXSOH, PRXSS, and TRXSS-mediated redox signaling relay and for the emergence of both bistability and oscillation [19,64]. Ultrasensitivity is defined as a nonlinear input–output relationship where a small percentage change in the input signal leads to a larger percentage change in the output signal, and it often exhibits a sigmoidal steady-state dose response [19,65]. The circadian oscillation model of the P3TRS system by del Olmo et al. suffers from several caveats, where a number of rate constants and molecular abundances are parameterized at non-physiological values [63]. For instance, the sulfinylation and SRX-mediated reduction of PRX3SO_2_H were described as very fast reactions, and the mitochondrion-to-cytosol translocation of H_2_O_2_ was unrealistically slow. It is thus unclear whether, with physiologically aligned parameter conditions, the NFL circuit of the P3TRS system can still oscillate robustly within a nearly 24-h period.

In the present study, we aimed to address the multiple knowledge gaps identified above regarding H_2_O_2_ and PTRS dynamics and presented several novel findings. Through in-depth mathematical analyses of the CSSC using physiologically aligned parameter values and more realistic reaction kinetics, we elucidated multiple sources of molecular interactions intrinsically embedded in the CSSC that render ultrasensitivity, which enables possible bistability and oscillation. Through exploring the physiological conditions for circadian oscillation of the P3TRS system, we showed that such oscillation may emerge through supercritical Hopf bifurcations under high H_2_O_2_ production. Our modeling study provides novel insights into the quantitative behaviors of the PTRS system, which can guide and help prioritize future experimental studies of complex redox signaling.

## 2. Methods

### 2.1. Model Structures

#### 2.1.1. Ultrasensitivity and Bistability Models of the PTRS System

Given the molecular details of the PTRS system, as described in the Introduction, we modeled the CSSC of typical 2-Cys PRX and H_2_O_2_ turnover as follows (Figure 1A). Since in most cases, the PRX homodimer is obligatorily pre-formed and does not appear to dissociate during the oxidation–reduction cycles, the dimer can be treated as a single entity without considering the dimerization process per se. Here, we use italicized names to refer to the state variables and parameters in the mathematical models. The production of *H*_2_*O*_2_ follows a zero-order reaction with a rate constant *k*_0_. Degradation of *H*_2_*O*_2_ by all non-PRX enzymes is collectively described by a first-order reaction with a rate constant of *k*_5_. The formation of *PRXSOH* by *PRXSH* and *H*_2_*O*_2_ is described by a second-order reaction with a rate constant *k*_1_. The reduction in *PRXSOH* to *PRXSH* is described with two sequential reactions: the first is a first-order reaction where *PRXSH* resolves to *PRXSS* with a rate constant *k*_2*a*_, and the second is a second-order reaction where *PRXSS* and *TRXSH* react to produce *PRXSH* and *TRXSS* with a rate constant *k*_2*b*_. *TRXSS* is then reduced to *TRXSH* by *TR* described as a total quasi-steady-state approximation (tQSSA) kinetics with parameters *k*_2*c*_ as the catalytic rate constant and *K_m_*_2*c*_ as the Michaelis constant. tQSSA is a more accurate simplification than the Michaelis–Menten kinetics for modeling enzymatic reactions, especially when the substrate abundance is not necessarily in excess of the enzyme abundance [66]. The formation of *PRXSO*_2_*H* by *PRXSOH* and *H*_2_*O*_2_ is described by a second-order reaction with a rate constant of *k*_3_. The reduction of *PRXSO*_2_*H* to *PRXSOH* catalyzed by *SRX* is described as an explicit enzymatic reaction, where *PRXSO*_2_*H* and *SRX* first associate reversibly to form an intermediate complex *PRXSO*_2_*H:SRX* with a second-order association rate constant *k*_4*f*_ and a first-order dissociation rate constant *k*_4*b*_, and *PRXSO*_2_*H:SRX* then dissociates into *PRXSOH* and *SRX* with a first-order catalytic rate constant *k*_4*c*_.

#### 2.1.2. Oscillation Model of the PTRS System

This model captures the global NFL mediated by H_2_O_2_, SRX, and PRX3 [8,67]. H_2_O_2_, released from the mitochondrion into the cytosol, first oxidizes SRX, which then associates with HSP90 by slowly forming a disulfide bond between Cys99 of SRX and the M domain of HSP90 (SRXSSHSP90). Through the interaction between the amphipathic sequence of the N-terminal 20 residues of SRX and the mitochondrial translocase outer membrane (TOM) complex, the SRXSSHSP90 complex then translocates across the mitochondrial outer and inner membranes into the matrix. In the matrix, dissociated SRX participates in the reduction of sulfinylated PRX3. Imported SRX is degraded by Lon but is somehow stabilized when in complex with sulfinylated PRX3 [8].

Given the molecular details described above, we modeled the SRX-mediated feedback as follows (Figure 1C). The movement of H_2_O_2_ between the mitochondrion and cytosol is described as a bi-directional first-order process with identical nominal rate constant *k*_6_ for both directions; however, because of the volume differences between the cytosolic and mitochondrial compartments, the mass fluxes of H_2_O_2_ are volume-adjusted by a factor, *V_ratio_*, which represents the mitochondrial matrix/cytosol volume ratio (see Table S4 for details). Cytosolic *H*_2_*O*_2_ (*H*_2_*O*_2*cyto*_) is degraded in a first-order process with a rate constant *k*_7_. SRX in the cytosol (*SRX_cyto_*) is synthesized with a zero-order rate constant *k*_8_ and degraded with a first-order rate constant *k*_9_. *SRX_cyto_* is oxidized by *H*_2_*O*_2*cyto*_ into *SRXSOH* with a second-order rate constant *k*_10_ and the reduction is a first-order reaction with a rate constant *k*_11_. *SRXSOH* is degraded with a first-order rate constant *k*_13_. *SRXSOH* and *HSP*90 associate to form the *SRXSSHSP*90 complex with a second-order associate rate constant *k*_12*f*_ and a first-order dissociation rate constant *k*_12*b*_. *SRXSSHSP*90 is degraded with a first-order rate constant *k*_15_. *SRXSSHSP*90 translocates into the mitochondrion with a first-order rate constant *k*_14_ with volume adjustment, where *HSP*90 and *SRX* dissociate with the latter renamed to *SRX_mito_*. *SRX_mito_* is degraded with a first-order rate constant *k*_16_. Besides the interactions of *SRX_mito_* with *PRX*3*SO*_2_*H* as described above, SRX in *PRX*3*SO*_2_*H:SRX* can also be degraded with a first-order rate constant *k*_4*d*_ where *PRX*3*SO*_2_*H* is recycled.

### 2.2. Model Parameters and Ordinary Differential Equations (ODEs)

The model parameters, including rate constants and molecular concentrations, are provided in Supplemental Table S1. The parameter values were obtained or derived from the primary experimental literature with the references and details of justifications provided in the Table footnote. The unit of concentration of the state variables is µM and unit of time is second (s). The ODEs and algebraic equations are presented in Tables S2–S5.

### 2.3. Sensitivity Analysis

Local sensitivity analysis was performed by increasing and decreasing one parameter at a time by 1% or 5% from the default value. Relative sensitivity coefficients for various endpoints, including the maximum or minimum local response coefficients and the corresponding *k*_0_ in the Ultrasensitivity Model, and the relative amplitude, absolute amplitude, and period of mitochondrial *H*_2_*O*_2_ in the oscillation model, were calculated by averaging the normalized percentage changes in the endpoints in both directions.

### 2.4. Modeling Tools and Code Sharing

The deterministic models were constructed and simulated in MATLAB version 2022b (The Mathworks, Inc, Natick, MA, USA) using the ordinary differential equation solver “ode15s”. XPP-AUT was used to conduct bifurcation analysis [68]. All simulation results were graphically rendered in MATLAB. All model codes are available at the GitHub repository: https://github.com/pulsatility/2024-Mathematical-Modeling-of-PTRS.git (accessed on 27 January 2025).

## 3. Results

### 3.1. Ultrasensitivity of the PTRS System

#### 3.1.1. Ultrasensitive Responses

**Figure 2:**
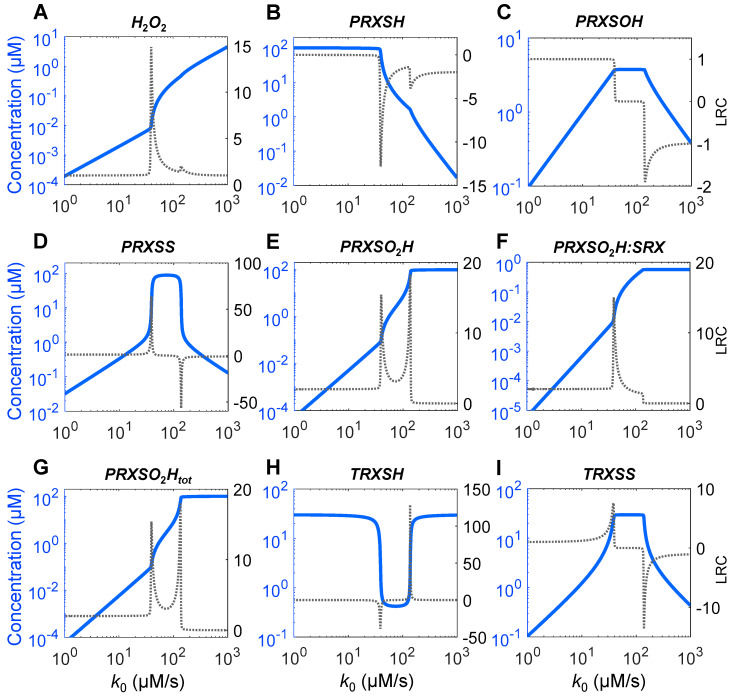
**Ultrasensitive responses of the PTRS model.** (**A**–**I**) Steady-state concentrations (solid blue lines) of the variables as indicated with respect to *k*_0_ and the associated *LRC* (dash gray lines). All parameters except *k*_0_ use the default values indicated for “Ultrasensitivity Model” in Table S1.

With the default parameter values as defined for the Ultrasensitivity Model in Table S1, as the *H*_2_*O*_2_ production rate *k*_0_ increases, the steady-state levels of *H*_2_*O*_2_ and all species of PRX and TRX exhibit various degrees of ultrasensitivity (Figure 2). Ultrasensitivity occurs in segments of the response curves having slope of local response coefficient (*LRC*) >1 or <−1 on the log–log scale, where a small percentage change in *k*_0_ results in a larger percentage change in the concentrations of these variables.

The *H*_2_*O*_2_ response is sigmoidal—it is linear when *k*_0_ is low but becomes ultrasensitive when *k*_0_ approaches 35 µM/s, with the *LRC* quickly reaching a maximum (*LRC_max_*) of 15 at *k*_0_ near 40 µM/s (Figure 2A). *LRC* then declines toward unity and along the way a second but much smaller peak occurs when *k*_0_ is near 139 µM/s. *PRXSH* monotonically decreases as *k*_0_ increases, reaching a negative minimum *LRC* (*LRC_min_*) of −12.8 and a secondary *LRC_min_* of −3.8 (Figure 2B) at *k*_0_ values associated with the two corresponding *LRC_max_* values for *H*_2_*O*_2_ above. *PRXSOH* exhibits a nonmonotonic trapezoid response, which first maintains a linear profile all the way till reaching a plateau, and then declines almost linearly with a small blip of ultrasensitivity (Figure 2C). In contrast, the *PRXSS* response is nonmonotonically inverted U-shaped and displays highly strong ultrasensitivity, with *LRC_max_* and *LRC_min_* reaching 64 and −57, respectively (Figure 2D) at *k*_0_ values associated with the two corresponding *LRC_max_* for *H*_2_*O*_2_. Total *PRXSO*_2_*H* (*PRXSO*_2_*H_tot_*, Figure 2G) is dominated by the free form (Figure 2E), and the *PRXSO*_2_*H:SRX* complex is only a small fraction (Figure 2F). *PRXSO*_2_*H* increases monotonically in two distinct phases, exhibiting *LRC_max_* of 15.4 and 18.2, respectively, at *k*_0_ values associated with the corresponding *LRC_max_* for *H*_2_*O*_2_, while *PRXSO*_2_*H:SRX* only shows one *LRC* peak. *TRXSH* exhibits a steep U-shaped response, with *LRC_min_* and *LRC_max_* reaching −38.6 and 128.8, respectively (Figure 2H). In contrast, *TRXSS* exhibits an inverted U-shaped response, with *LRC_max_* and *LRC_min_* reaching 7.6 and −13.7, respectively (Figure 2I). Although these *LRC_max_* or *LRC_min_* values occur near the *k*_0_ values mentioned above, the overall ultrasensitivity of *TRXSS* appears to start at lower *k*_0_ values, at about 16.7 µM/s where *LRC* begins to surpass 1.5.

#### 3.1.2. Origins of Ultrasensitivity

**Figure 3:**
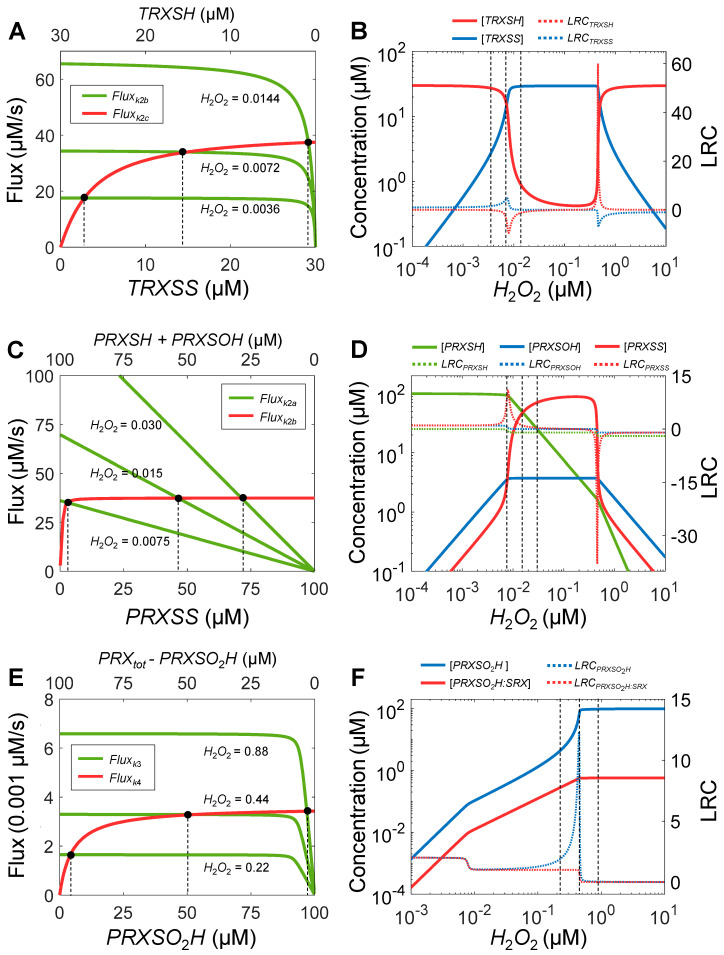
**Origins of ultrasensitivity of the PTRS model.** (**A**) Flux analysis of *TRXSH* oxidation and *TRXSS* reduction. *Flux_k_*_2*b*_ and *Flux_k_*_2*c*_ were obtained by clamping *TRXSS* at different concentrations and *H*_2_*O*_2_ at concentrations (µM) as indicated and then letting the system reach steady states. Solid dots denote steady states and vertical dash lines indicate the corresponding *TRXSS* concentrations. Same denotation for panel (**C**,**E**). (**B**) Ultrasensitivity of *TRXSH* and *TRXSS* in response to *H*_2_*O*_2_ clamped at different concentrations. The 3 vertical dash lines indicate the *H*_2_*O*_2_ concentrations as shown in (**A**). Similar denotation for panel (**D**,**F**). (**C**) Flux analysis of *PRXSOH* resolution and *PRXSS* reduction. *Flux_k_*_2*a*_ and *Flux_k_*_2*b*_ were obtained by clamping *PRXSS* at different concentrations, *H*_2_*O*_2_ at concentrations (µM) as indicated, and *PRXSO*_2_*H* and *PRXSO*_2_*H:SRX* at zero, then letting the system reach steady states. (**D**) Ultrasensitivity of *PRXSH*, *PRXSOH*, and *PRXSS* in response to *H*_2_*O*_2_ clamped at different concentrations. (**E**) Flux analysis of *PRXSOH* sulfinylation and *PRXSO*_2_*H* reduction. *Flux_k_*_3_ and *Flux_k_*_4_ were obtained by clamping *PRXSO*_2_*H* at different concentrations, *H*_2_*O*_2_ at concentrations (µM) as indicated, and *PRXSH* at zero, then letting the system reach steady states. (**F**) Ultrasensitivity of *PRXSO*_2_*H* and *PRXSO*_2_*H:SRX* in response to *H*_2_*O*_2_ clamped at different concentrations.

We next analyzed the origins of these ultrasensitivities exhibited by the variables of the PTRS system. There exist three redox cycles driven directly or indirectly by *H*_2_*O*_2_: (i) *TRXSH* → *TRXSS* → *TRXSH*, (ii) *PRXSH* → *PRXSOH* → *PRXSS* → *PRXSH*, and (iii) *PRXSOH* → *PRXSO*_2_*H* → *PRXSOH*. In theory, when one or both reactions in a cycle operate near saturation by the substrates, zero-order ultrasensitivity can arise where the substrate accumulates sharply as its removal approaches a constant rate due to zero-order kinetic [69,70]. Using flux analysis, we examined this possibility for each redox cycle above. For the *TRXSH* → *TRXSS* → *TRXSH* cycle, the flux of the reduction (named *Flux_k_*_2*c*_ here) appears as a typical rectangular hyperbola, where it approaches saturation at high concentrations of the substrate *TRXSS* (Figure 3A). The saturation is due to the low Michaelis constant *K_m_*_2*c*_ (3 µM) and low enzyme concentration (*TR_to_*_t_ = 2.76 µM) relative to the substrate *TRXSS* concentration, which can go up to 30 µM. For the reverse, oxidative reaction where *TRXSH* is the substrate, its flux (*Flux_k_*_2b_) exhibits a stronger saturable profile with a plateau for most of the concentration range of *TRXSH* (Figure 3A). This saturation occurs because the maximal reaction rate is ultimately determined and thus limited by the capacity of *H*_2_*O*_2_-driven *Flux_k_*_1_, which is capped by *k*_1_*H_2_*O*_2_*PRX_tot_. Thus, the height of the plateau depends on the clamped *H*_2_*O*_2_ level. The intersection points of the *Flux_k_*_2c_ and *Flux_k_*_2b_ curves represent the steady states. At 0.0072 µM *H*_2_*O*_2_, the two flux curves intersect very tightly because of the saturation. Then, as *H*_2_*O*_2_ increases or decreases by 2-fold, the intersection point swings widely where the fold changes in the corresponding steady-state *TRXSS* and *TRXSH* concentrations are greater than 2 or much less than 0.5, indicating ultrasensitivity. Driving the PTRS system to steady states by clamping *H*_2_*O*_2_ at different concentrations confirmed the ultrasensitivity, which is in the *H*_2_*O*_2_ concentration range of 0.0036–0.014 µM, where *LRC* for *TRXSS* peaks at *LRC_max_* = 4.69, and *LRC* for *TRXSH* dips to *LRC_min_* = −9.5 (Figure 3B). Past this ultrasensitivity range, *TRXSS* immediately reaches a plateau at a concentration nearly equal to *TRX_tot_*. The ultrasensitivity at much higher *H*_2_*O*_2_ concentrations (0.35–0.6 µM) is due to the involvement of the sulfinylation cycle, which is analyzed below. Note here that driving the system with *H*_2_*O*_2_ clamped at different concentrations as opposed to with *k*_0_ as in Figure 2 allows us to distinguish the ultrasensitivities intrinsic to the TRX and PRX redox cycles, not those secondary to the ultrasensitivity of *H*_2_*O*_2_ itself.

We next examined the *PRXSH* → *PRXSOH* → *PRXSS* → *PRXSH* cycle. The flux of the reduction reaction *Flux_k_*_2*b*_ is saturated for most of the range of its substrate *PRXSS* (Figure 3C), as it is limited by the maximal *Flux_k_*_2*c*_, at about 37.5 µM/s. In contrast, the flux of the resolution reaction *Flux_k_*_2*a*_ is linear with the slope proportional to the clamped *H*_2_*O*_2_ level. The nearly zero-order *Flux_k_*_2*b*_ with respect to *PRXSS* results in a wide swing of the steady-state intersection points as *H*_2_*O*_2_ varies within a certain range, leading to a strong ultrasensitive response of *PRXSS* with *LRC_max_* of 10.9 in the vicinity of 0.0075 µM *H*_2_*O*_2_ (Figure 3D). Interestingly, *PRXSH* and *PRXSOH* remain either linear or flat as *H*_2_*O*_2_ varies. The plateau of *PRXSOH* results from *Flux_k_*_2*c*_ maxing out, such that *Flux_k_*_2*a*_, which is equal to *Flux_k_*_2*b*_ and *Flux_k_*_2*c*_, remains constant up to *H*_2_*O*_2_ approaching 0.42 µM.

Lastly, we examined the *PRXSOH* → *PRXSO*_2_*H* → *PRXSOH* cycle. The flux of the reduction reaction *Flux_k_*_4_ is saturated for most of the range of its substrate *PRXSO*_2_*H* (Figure 3E). The saturation is due to the low Michaelis constant as determined by (*k*_4*b*_ + *k*_4*c*_)/*k*_4*a*_ = 5 µM and low enzyme concentration (*SRX_to_*_t_ = 0.6 µM) relative to the substrate *PRXSO*_2_*H* concentration which can go up to 100 µM. The sulfinylation reaction flux *Flux_k_*_3_ exhibits a stronger saturable profile when *H*_2_*O*_2_ is at high concentrations (Figure 3E). The saturation occurs because the reaction rate is determined by both *H*_2_*O*_2_ and *PRXSOH* where the latter reaches a plateau at high *H*_2_*O*_2_ levels as indicated in Figure 3D. The nearly zero-order responses of both *Flux_k_*_3_ and *Flux_k_*_4_ result in a wide swing of the steady-state intersection points as *H*_2_*O*_2_ varies, leading to a strong ultrasensitive response of *PRXSO*_2_*H* with *LRC_max_* of 12.3 in the vicinity of 0.44 µM *H*_2_*O*_2_ (Figure 3F), which also explains the second ultrasensitivity peaks in Figure 3B,D.

Taken together, all these redox cycles can operate in a range near saturation, producing intrinsic zero-order ultrasensitivity in response to *H*_2_*O*_2_ challenges at different concentrations. When the CSSC is driven by increased *H*_2_*O*_2_ production (*k*_0_), *H*_2_*O*_2_ itself also exhibits ultrasensitivity because its elimination by the PRTS system approaches saturation when the peroxidatic cycles are saturated.

#### 3.1.3. Sensitivity Analysis of the Ultrasensitive Responses

**Figure 4:**
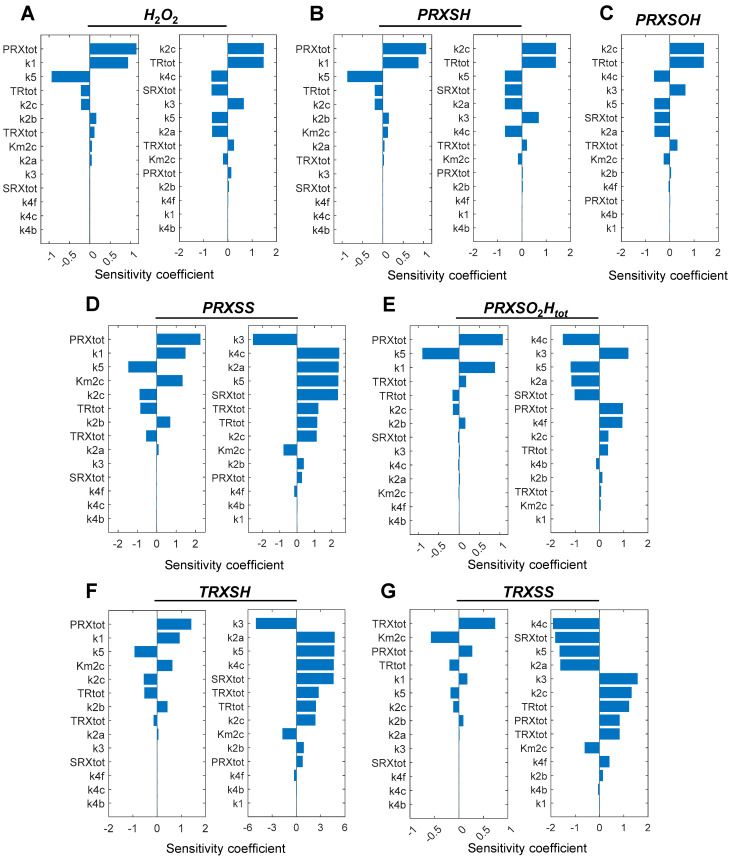
**Sensitivity analyses of ultrasensitivity of the PTRS model.** Left panels in (**A**,**B**,**D**–**G**): relative sensitivity coefficients for *LRC_max_* or *LRC_min_* occurring at the lower *k*_0_ value near 40 µM/s as in Figure 2. Right panels in (**A**,**B**,**D**–**G**) and (**C**): relative sensitivity coefficients for *LRC_max_* or *LRC_min_* occurring at the higher *k*_0_ value near 139 µM/s as in Figure 2. The sensitivity analysis was conducted as follows: after each parameter was increased or decreased by 1% from the default value, the entire response curve as in Figure 2 was generated by scanning *k*_0_ in the range of 1–1000 µM/s, then the new *LRC_max_* or *LRC_min_* values were captured to calculate the relative sensitivity coefficients as described in Section 2.3.

We next conducted a sensitivity analysis to reveal parameters that affect the degree of ultrasensitivity, i.e., the *LRC_max_* or *LRC_min_*, the most. For the ultrasensitivity occurring at the lower *k*_0_ value, i.e., in the vicinity of 40 µM/s (Figure 2) where redox cycle (i) and (ii) are primarily involved, *PRX_tot_*, *k*_1_, and *k*_5_ appear to be the most influential parameters for most of the variables (Figure 4A,B,D,E, left panels). An exception is *TRXSS*, where *TRX_tot_*, *k_m_*_2*c*_, and *PRX_tot_* are the most sensitive ones. For the ultrasensitivity occurring at the higher *k*_0_ value, i.e., in the vicinity of *k*_0_ = 139 µM/s (Figure 2) where redox cycle (iii), the sulfinylation cycle, is primarily involved, *k*_2*c*_ and *TR_tot_* are the most influential parameters for *H*_2_*O*_2_, *PRXSH*, and *PRXSOH* (Figure 4A–C, right panels). In comparison, *k*_3_, *k*_4*c*_, *k*_5_, *k*_2*a*_, and *SRX_tot_* are among the top sensitive parameters for *PRXSS*, *PRXSO_2_H, TRXSH*, and *TRXSS* (Figure 4D–G, right panels). We also conducted a sensitivity analysis for *k*_0_ associated with these *LRC_max_* or *LRC_min_*, which showed that *k*_2*c*_ and *TR_tot_* are consistently among the most sensitive parameters for the ultrasensitivity occurring at the lower *k*_0_ value in the vicinity of 40 µM/s, whereas for the ultrasensitivity occurring at the higher *k*_0_ value in the vicinity of 139 µM/s, *SRX_tot_*, *k*_5_, *k*_4*c*_, *k*_3_, and sometimes *TR_tot_* and *k*_2*a*_, are among the top-ranking parameters (Figure S1).

#### 3.1.4. Dynamical Responses to Varying H_2_O_2_ Production Rate

We next examined how fast the ultrasensitive PTRS system responds to changes in *H*_2_*O*_2_ production rate. As *k*_0_ is stepped up from a basal 10 µM/s to higher values, most species except *PRXSO*_2_*H* reach steady states almost instantaneously in a few seconds (Figure S2). It appears that intermediate *k*_0_ values (yellow line) are associated with a more sluggish response than lower or higher *k*_0_ values. As *k*_0_ is switched back to 10 µM/s, the activated state of the PTRS system turns off almost immediately. The simulations indicate that when the PTRS system operates in the ultrasensitive domain, the switching of *H*_2_*O*_2_, *PRXSOH*, *PRXSS*, and *TRXSS* is fast, which is commensurate with their postulated roles as a redox second messenger or signaling intermediate.

### 3.2. Bistability of the PTRS System

#### 3.2.1. Bistable Switch

**Figure 5:**
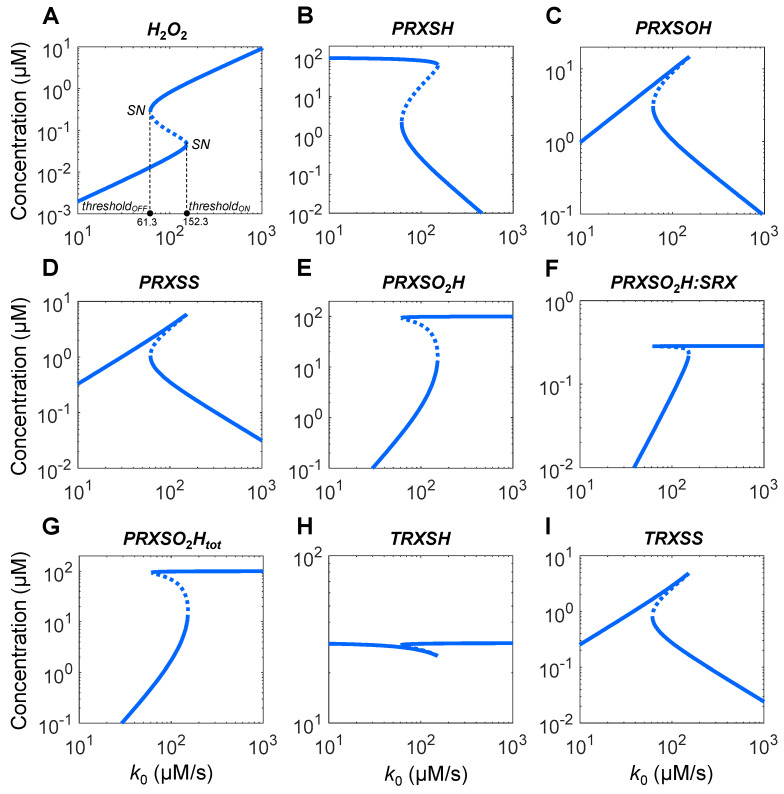
**One-parameter bifurcation analysis of bistability of the PTRS model.** (**A**–**I**) Saddle–node bifurcation of steady-state levels of *H*_2_*O*_2_, *PRXSH*, *PRXSOH*, *PRXSS*, *PRXSO*_2_*H*, *PRXSO*_2_*H:SRX*, *PRXSO*_2_*H_tot_*, *TRXSH*, and *TRXSS* as indicated with respect to *k*_0_. *SN*: saddle–node; solid blue line: stable steady state; dashed blue line: unstable steady state; vertical dashed black lines: denote *threshold_OFF_* and *threshold_ON_* for *k*_0_ at the two *SN* bifurcation points.

To make the PTRS system bistable, parameters *k*_2*c*_, *k*_5_, *TR_tot_*, and *SRX_tot_* were varied from the default values in the Ultrasensitivity Model, but still within the ranges reported in the literature (for details see Bistability Model in Table S1). With these parameter values, the model is capable of typical hysteretic steady-state response behaviors, as demonstrated by one-parameter bifurcation analysis with respect to *k*_0_ (Figure 5). 

The bifurcation diagrams indicate that as *k*_0_ increases, *H*_2_*O*_2_ first increases linearly with a slope of 1; however, upon *k*_0_ reaching about 152.3 µM/s (named *threshold_ON_* here), *H*_2_*O*_2_ switches sharply from about 0.045 to 1.34 µM (Figure 5A). At this high level, *H*_2_*O*_2_ resumes its linear response to *k*_0_. The linearity remains till *k*_0_ drops to a threshold near 61.3 µM/s (*threshold_OFF_*), where *H*_2_*O*_2_ switches off from 0.28 to 0.01 µM. The *threshold_ON_* and *threshold_OFF_* are two saddle–node bifurcation points delimiting a bistable zone in between. As *k*_0_ increases from low levels but prior to reaching *threshold_ON_*, *PRXSH* barely changes (Figure 5B), while *PRXSOH* and *PRXSS* increase linearly (Figure 5C,D), and *PRXSO*_2_*H* and *PRXSO*_2_*H:SRX* also increase but more steeply with a slope of 2 on log–log scale (Figure 5E,F). At *threshold_ON_*, *PRXSH*, *PRXSOH*, and *PRXSS* drop sharply, while *PRXSO*_2_*H* jumps from 13.4 µM to nearly 100 µM which is the total PRX level (*PRX_tot_*) defined in the model. When *k*_0_ is above *threshold_ON_*, *PRXSH*, *PRXSOH,* and *PRXSS* decrease as *k*_0_ increases further while *PRXSO*_2_*H* and *PRXSO*_2_*H:SRX* stay relatively constant. When *k*_0_ drops below *threshold_OFF_*, *PRXSH* switches back up to near *PRX_tot_*, and *PRXSO*_2_*H* drops sharply to about 0.45 µM. Compared with the wide dynamic range of *PRXSH*, *TRXSH* only switches on and off in a very narrow range (Figure 5H). *TRXSS* exhibits a similar response profile to *PRXSS*, where it either increases or decreases linearly with *k*_0_ before switching occurs (Figure 5I).

#### 3.2.2. Origins of Ultrasensitivity That Enables Bistability

**Figure 6:**
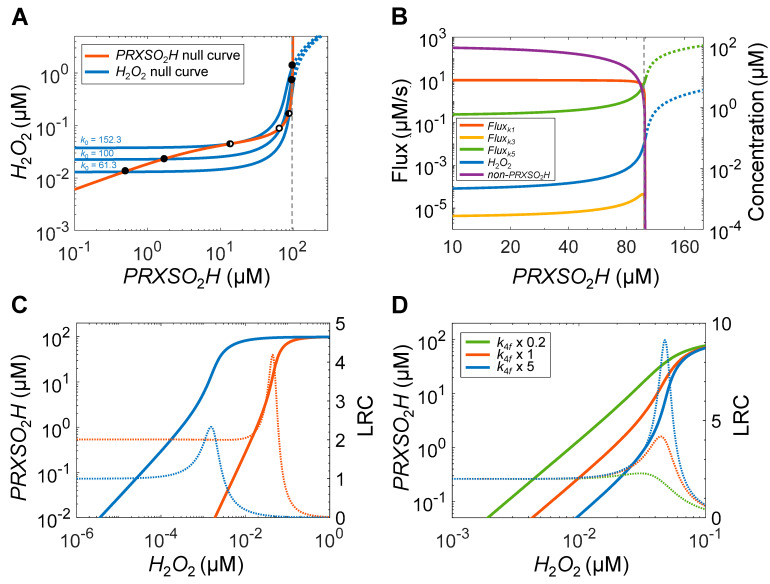
**Origins of ultrasensitivity in the bistable PTRS model.** (**A**) Ultrasensitive *PRXSO*_2_*H* null curve and *H*_2_*O*_2_ null curves obtained at 3 different *k*_0_ values (µM/s) as indicated. Intersection points represent steady states. Solid dot: stable steady state; empty dot: unstable steady state; half-empty dot: saddle–node unstable steady state. Dashed blue line: *PRXSO*_2_*H* levels exceeding *PRX_tot_* = 100 µM as defined in the model, which exists only in theory. (**B**) Analysis of the origin of ultrasensitivity of the *H*_2_*O*_2_ null curve. It arises due to the sharp decrease in the non-sulfinylated species (*non-PRXSO*_2_*H = PRXSH* + *PRXSOH* + *PRXSS*, purple line) as *PRXSO*_2_*H* approaches *PRX_tot_* and the consequential steep decrease in the rate of *H*_2_*O*_2_ elimination by *PRXSH* (*Flux_k_*_1_, orange line). (**C**) Origin of ultrasensitivity of the *PRXSO*_2_*H* null curve due to dual-step signaling of *H*_2_*O*_2_, leading to generation of *PRXSO*_2_*H*. Solid orange line: default *PRXSO*_2_*H* null curve; solid blue line: *PRXSO*_2_*H* null curve generated with *H*_2_*O*_2_ removed from participating in the sulfinylation reaction; dotted lines: corresponding *LRC*. (**D**) Origin of ultrasensitivity of the *PRXSO*_2_*H* null curve due to zero-order (saturable) reduction of *PRXSO*_2_*H* by *SRX*. *PRXSO*_2_*H* null curves were obtained with *k*_4*f*_ varied to different folds relative to the default value (×1) as indicated. Solid lines: *PRXSO*_2_*H* null curve; dotted lines: corresponding *LRC*.

In addition to a PFL or DNFL topology, another requirement for the emergence of bistability is that at least one arm of the feedback loop must embed ultrasensitivity, which can provide percentage-wise signal amplification [19,64]. For the feedback loop of the PTRS system as illustrated in Figure 1B, it means that either arm 1 or arm 2 or both need to be ultrasensitive. Though related, this is different than the ultrasensitive responses driven externally by varying *k*_0_ as explored in Section 1. One way to explore ultrasensitivity in an arm is to examine the nullclines (or null curves for 3-variable or higher-dimension systems) of the state variables by obtaining a steady-state input/output relationship for the arm. We obtained the null curve of *H*_2_*O*_2_, which is the output variable of arm 1, by clamping *PRXSO*_2_*H* at different fixed levels (keeping the sum of *PRXSH*, *PRXSOH*, *PRXSS*, *PRXSO*_2_*H*, and *PRXSO*_2_*H:SRX* constant, i.e., equal to *PRX_tot_*) and then letting the system to reach steady state. The *H*_2_*O*_2_ null curves are steeply sigmoidal when *PRXSO*_2_*H* approaches *PRX_tot_* (Figure 6A, blue lines). When *k*_0_ = 100 µM/s, the *LRC_max_* on the curve is 8.47. For *k*_0_ at *threshold_OFF_* value of 61.3 and at *threshold_ON_* value of 152.3 µM/s, the *LRC_max_* are 11.49 and 6.32, respectively. We then obtained the *PRXSO*_2_*H* null curve for arm 2 by clamping *H*_2_*O*_2_ at different fixed levels to obtain steady-state levels of *PRXSO*_2_*H*. The *PRXSO*_2_*H* null curve (Figure 6A, orange line) is also sigmoidal and the *LRC_max_* is 4.18 when *H*_2_*O*_2_ is at 0.045 µM. Since the *LRC_max_* values are greater than unity for both the *H*_2_*O*_2_ and *PRXSO*_2_*H* null curves, both arms of the PFL are thus ultrasensitive. When *k*_0_ = *threshold_OFF_* or *k*_0_ = *threshold_ON_*, the *H*_2_*O*_2_ and *PRXSO*_2_*H* null curves intersect once but are also tangential to each other at a different location, demarcating the two saddle–node bifurcation points (indicated by half-empty dots). When *threshold_OFF_* < *k*_0_ < *threshold_ON_*, the two sigmoidal null curves are aligned in such a way that they intersect with each other three times (Figure 6A). As a result, two stable steady states (solid dots) and one unstable steady state (empty dot) are born out, indicating bistability.

We next analyzed the origins of the ultrasensitivity of the two null curves. The ultrasensitivity of the *H*_2_*O*_2_ null curve is due to the following. When the *PRXSO*_2_*H* level approaches *PRX_tot_* (100 µM here), a small percentage increase of *PRXSO*_2_*H*, e.g., from 90 to 91 µM (+1.11%), results in a much larger percentage decrease in non-sulfinylated PRX species, from 10 to 9 µM (−10%), with negligible *PRXSO*_2_*H:SRX*. This leads to a highly ultrasensitive response of non-*PRXSO*_2_*H* (Figure 6B, purple line). Since *H*_2_*O*_2_ is predominately eliminated by *PRXSH* through the peroxidatic cycle, the rate of *H*_2_*O*_2_ elimination by the cycle, represented by *Flux_k_*_1_, also exhibits an ultrasensitive decline as *PRXSO*_2_*H* increases (Figure 6B, orange line). As a result, the steady-state *H*_2_*O*_2_ level increases steeply with a high degree of ultrasensitivity (Figure 6B, blue line). When *Flux_k_*_1_, i.e., the *PRXSH*-mediated *H*_2_*O*_2_ elimination, drops to very low levels, non-PRX-mediated *H*_2_*O*_2_ elimination, as represented by *Flux_k_*_5_, starts to take over and increase sharply (Figure 6B, green line). The *H*_2_*O*_2_ elimination through sulfinylation, *Flux_k_*_3_, is negligible in the process (Figure 6B, yellow line). In summary, the ultrasensitivity of the *H*_2_*O*_2_ null curve for the *PRXSO*_2_*H* → *H*_2_*O*_2_ arm originates from the fact that when PRX is highly sulfinylated, the capacity of *PRXSH*-mediated *H*_2_*O*_2_ elimination is reduced tremendously and becomes sensitive to small changes in the *PRXSO*_2_*H* level.

The ultrasensitivity of the *PRXSO*_2_*H* null curve comes from two sources. One is multistep signaling, where an input signal simultaneously regulates two or more biochemical processes that synergistically control a common output [19,65]. In the present model, *H*_2_*O*_2_ simultaneously participates in the sequential processes that first sulfenylate *PRXSH* into *PRXSOH* and then sulfinylate *PRXSOH* into *PRXSO*_2_*H*. Therefore, as the common input, *H_2_O_2_* regulates the production of *PRXSO*_2_*H* in two synergistic steps. Examining the *PRXSO*_2_*H* null curve closely reveals that at low *H_2_O_2_* concentrations, steady-state *PRXSO*_2_*H* indeed increases with a slope (*LRC*) of 2 (Figure 6C, orange line), consistent with the dual-step signaling scenario. Moreover, when we artificially disabled the participation of *H*_2_*O*_2_ in the sulfinylation reaction while still allowing it to occur as a first-order process by itself, the slope of the *PRXSO*_2_*H* null curve at low *H_2_O_2_* concentrations is reduced to 1 (Figure 6C, blue line). This analysis confirms the notion that the dual-step *H*_2_*O*_2_ signaling to generate *PRXSO*_2_*H* contributes to the ultrasensitivity of the *PRXSO*_2_*H* null curve.

In the absence of dual-step *H_2_O_2_* signaling, the remaining ultrasensitivity of the *PRXSO*_2_*H* null curve (Figure 6C, blue dotted line) comes from the saturable sulfinylation reaction as analyzed above in Section 1 (Figure 3E,F). With the same Michaelis constant (*k*_4*b*_ + *k*_4*c*_)/*k*_4*f*_ = 5 µM and an even lower total enzyme *SRX_tot_* at 0.3 µM compared with the Ultrasensitivity Model, *PRXSO*_2_*H*, which can go up to 100 µM, can readily saturate *SRX*, leading to zero-order ultrasensitivity. Varying the Michaelis constant by changing, for instance, *k*_4*f*_ value, clearly shows that the maximal slope of the *PRXSO*_2_*H* null curve, which is *LRC_max_* = 4.18 at default parameter value, becomes much steeper (*LRC_max_* = 9.14) when *k*_4*f*_ is increased by 5-fold, and becomes shallower (*LRC_max_* = 2.28) when *k*_4*f*_ is decreased by 5-fold (Figure 6D). Varying *K_m_* by changing *k*_4*b*_ and *k*_4*c*_ achieves similar slope-altering effects. If the *SRX*-mediated reduction is simplified to a 2nd order reaction with a fixed amount of *SRX*, the *PRXSO*_2_*H* null curve is only ultrasensitive with a slope of 2 (on log–log scale) due to the dual-step *H_2_O_2_* signaling that remains.

#### 3.2.3. Flux Analysis of H_2_O_2_ Turnover

While the above null-curve approach is able to reveal where ultrasensitivity resides within the feedback loop and consequently how bistability arises, it may not be very straightforward to interpret or predict the changes in the model’s stability in response to parameter variations. A more intuitive approach is flux analysis, which can help interpret the results of two-parameter bifurcation to be presented later. In the model, the turnover of *H*_2_*O*_2_ is as follows: it enters the system through the zero-order *k*_0_ process and exits through the *k*_1_, *k*_3,_ and *k*_5_ processes (Figure 1A). The relationships between the null curves presented above in Figure 6A, saddle–node bifurcation presented above in Figure 5A, and *H*_2_*O*_2_ turnover fluxes are described in Figure S3, corresponding to Figure S3A, S3B, and S3C, respectively. At low *H*_2_*O*_2_ concentrations, it is mostly eliminated through the peroxidatic cycle, i.e., *Flux_k_*_1_ (Figure S3C, orange line), while the eliminations through the non-PRX pathway, *Flux_k_*_5_ (Figure S3C, green line), and hyperoxidation, *Flux_k_*_3_ (Figure S3C, yellow line), are negligibly small. Initially, as *H*_2_*O*_2_ increases from low concentrations, both *Flux_k_*_1_ and *Flux_k_*_5_ increase linearly with a slope of 1, while *Flux_k_*_3_ increases ultrasensitively with a slope of 2 (on log–log scale) due to the dual-step signaling mentioned above. However, interestingly, as *H*_2_*O*_2_ increases further to near 0.047 µM (one of the two saddle–node bifurcation points), *Flux_k_*_1_ reverses the upward trend and starts to decrease, hence exhibiting a nonmonotonic, bell shape. The reversal occurs because with higher *H*_2_*O*_2_ more *PRXSOH* is sulfinylated to *PRXSO*_2_*H* and the abundance of *PRXSH* which carries out *H*_2_*O*_2_ elimination decreases.

In comparison, *Flux_k_*_3_ grows monotonically and then becomes saturated (Figure S3C, yellow line). However, it remains several orders of magnitude lower than *Flux_k_*_1_ and *Flux_k_*_5_ throughout the range of *H*_2_*O*_2_; thus, its contribution to *H*_2_*O*_2_ elimination is negligible. Therefore, the total *H*_2_*O*_2_ removal rate (i.e., *Flux_total_* = *Flux_k_*_1_ + *Flux_k_*_5_ + *Flux_k_*_3_) is determined predominantly by the sum of the first two. At low *H*_2_*O*_2_ concentrations, *Flux_k_*_1_ dominates over *Flux_k_*_5_; thus, *Flux_total_* is also bell-shaped following the trend of *Flux_k_*_1_ with an inflection point at about 0.047 µM *H*_2_*O*_2_ (Figure S3C, blue line). As *H*_2_*O*_2_ concentration continues to increase to near 0.28 µM, *Flux_k_*_1_ and *Flux_k_*_5_ intersect and are thus equal, creating another inflection point for *Flux_total_*, beyond which it reverses the downtrend and starts to increase, following the trend of *Flux_k_*_5_. Overall, a twisted S-shaped curve is born for *Flux_total_*. The *H*_2_*O*_2_ production rate, *Flux_k_*_0_ (Figure S3C, horizontal dashed lines), crosses the S-shaped *Flux_total_* curve three times when *k*_0_ = 100 µM/s, generating three steady states. When *k*_0_ = 61.3 (*threshold_OFF_*) or 152.3 (*threshold_ON_*) µM/s, *Flux_k_*_0_ becomes tangential to the *Flux_total_* curve at the two inflection points, respectively, generating two saddle nodes. These steady states and their stability obtained through flux analysis correspond well to those obtained through the null-curve and bifurcation stability analysis (Figure S3A,B), demonstrating the equivalence of these approaches.

#### 3.2.4. Two-Parameter Bifurcation Analysis

**Figure 7:**
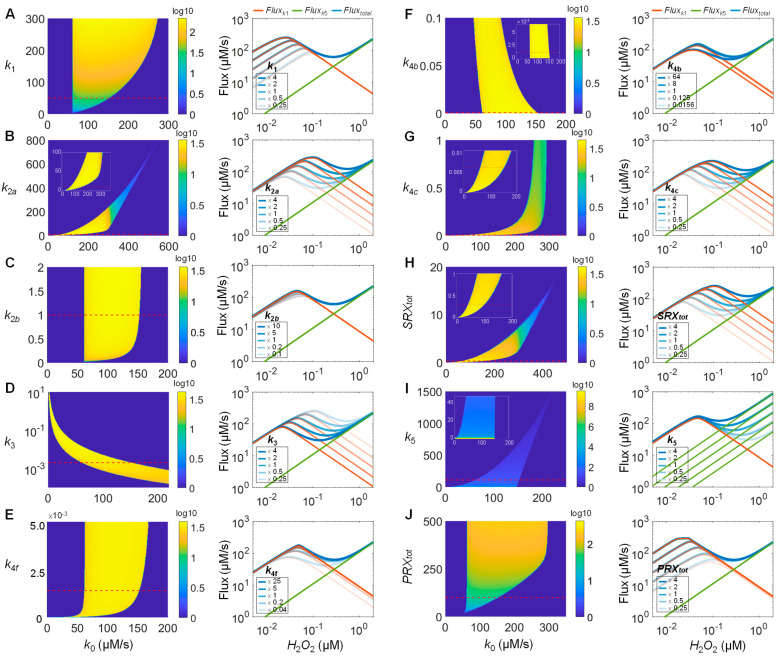
**Two-parameter bifurcation and flux analysis for bistability of the PTRS model.** (**A**–**J**, **Left panels**): Bistable and monostable zones and their boundaries with respect to *k*_0_ and a second parameter as indicated. The heatmap represents the *bistability magnitude* defined as the log10 ratio of the stable steady-state *H*_2_*O*_2_ levels in the high and low steady states. *Bistability magnitude* > 0: bistable; *bistability magnitude* = 0: monostable (dark blue region). Horizontal red dashed lines: default values of the second parameters. (**A**–**J**, **Right panels**): Flux analysis for the corresponding parameters varied in left panels. The color code for different fluxes is indicated on the top. The fold-change in the parameter value relative to the default value (×1) is indicated with lines of varying shade.

To further explore how the stability of the bistable PTRS system changes with respect to model parameters, two-parameter bifurcation analyses were performed for *k*_0_ versus other parameters. The analyses showed that bistability occurs within defined boundaries of these parameters and the system becomes monostable outside the boundaries (Figure 7, left panels). As indicated by the heatmap, the logarithmic ratio of the high to low steady-state *H*_2_*O*_2_ levels in the bistable zone (referred to as *bistability magnitude* herein) also varies and reduces to unity in the monostable zones (dark blue regions). To understand why the bistable zone boundaries change the way they do as parameters vary, we resort to the flux analysis by examining how each parameter affects the *H*_2_*O*_2_ elimination rates (Figure 7, right panels). Since, in most conditions, *Flux_k_*_1_ and *Flux_k_*_5_ >> *Flux_k_*_3_, we only need to focus on the former two fluxes to understand how *Flux_total_* is altered by these parameters.

Increasing *k*_1_, the rate constant for sulfenylation of *PRXSH*, clearly leads to higher *Flux_k_*_1_, but only on the ascending phase (Figure 7A, right panel). As a result, the peak of the S-shaped *Flux_total_* curve shifts up and leftward. Decreasing *k*_1_ has the opposite effect. The peak level of *Flux_total_* determines the *threshold_ON_* value of *k*_0_ for the saddle–node bifurcation, as shown in Figure S3, which corresponds to the right boundary of the bistable zone (Figure 7A, left panel). In comparison, the trough of the *Flux_total_* curve remains unchanged as *k*_1_ varies. The trough level determines the *threshold_OFF_* value of *k*_0_ for the saddle–node bifurcation, which corresponds to the left boundary of the bistable zone. Therefore, increasing *k*_1_ results in an expanding bistable zone but only on the right boundary, with increasing *bistability magnitude*.

Two parameters are involved in the reduction in *PRXSOH*, *k*_2*a*_ and *k*_2*b*_. Increasing *k*_2*a*_, the rate constant for the initial resolution step, pushes up the descending phase of *Flux_k_*_1_ (Figure 7B, right panel). As a result, both the peak and trough of the *Flux_total_* curve shift up and rightward. Decreasing *k*_2*a*_ has the opposite effect. The shifting leads to a bistable zone whose left and right boundaries shift to lower *k*_0_ values with higher *bistability magnitude* as *k*_2*a*_ decreases and to higher *k*_0_ values as *k*_2*a*_ increases but, eventually, bistability is lost at too high or too low *k*_2*a*_ values (Figure 7B, left panel and inset). Decreasing *k*_2*b*_, the rate constant for *PRXSS* reduction, does not have much impact initially, but larger fold decreases lead to decreases in the slope of the ascending phase of *Flux_k_*_1_ near the inflection point (Figure 7C, right panel). As a result, the peak of the *Flux_total_* curve continuously shifts down and rightward and the S shape eventually disappears. Increasing *k*_2*b*_ has an opposite but much minor effect, which eventually is saturated at higher *k*_2*b*_ values. Therefore, decreasing *k*_2*b*_ from the default value has a tendency to shrink the bistable zone which eventually completely disappears, while increasing *k*_2*b*_ expands the bistable zone a bit with the two borders eventually stabilizing at high *k*_2*b*_ values (Figure 7C, left panel). Likewise, varying *TRX_tot_*, *k*_2*c*_, *K_m_*_2*c*_, and *TR_tot_*, which are parameters related to *TRXSS* reduction, all produce similar effects as *k*_2*b*_ on the bistable zone boundaries (Figure S4). In general, bistability is favored when these parameters change in directions that facilitate the reduction in *PRXSS* to *PRXSH*.

Varying *k*_3_, the rate constant for sulfinylation, affects the descending phase of *Flux_k_*_1_, and thus both the peak and trough of the *Flux_total_* curve (Figure 7D, right panel). As a result, as *k*_3_ increases, the left and right boundaries of the bistable zone shift leftward, but eventually the bistability is lost for too high *k*_3_ values. (Figure 7D, left panel). Four parameters are involved in the reduction of *PRXSO*_2_*H*, *k*_4*f*_, *k*_4*b*_, *k*_4*c*_, and *SRX_tot_*. Varying *k*_4*f*_, the association rate constant for the binding between *PRXSO*_2_*H* and *SRX*, affects the descending phase of *Flux_k_*_1_ (Figure 7E, right panel). As a result, decreasing *k*_4*f*_ from the default value shrinks the bistable zone till it completely disappears, while increasing *k*_4*f*_ initially expands the bistable zone slightly, but the expansion asymptotically comes to an end at high *k*_4*f*_ values (Figure 7E, left panel). In the bistable zone, the *bistability magnitude* appears to be constant without a progressive decrease near the boundaries. Increasing *k*_4*b*_, the dissociation rate constant for *PRXSO*_2_*H:SRX*, reduces the peak and the descending phase of *Flux_k_*_1_ (Figure 7F, right panel). As a result, increasing *k*_4*b*_ shrinks and decreasing *k*_4*b*_ expands the bistable zone (Figure 7F, left panel), without loss of bistability even at very high *k*_4*b*_ values. Increasing *k*_4*c*_, the catalytic rate constant for the *SRX*-mediated reduction of *PRXSO*_2_*H*, shifts the peak and the descending phase of *Flux_k_*_1_ up and rightward and the opposite occurs with decreasing *k*_4*c*_ (Figure 7G, right panel). As a result, increasing *k*_4*c*_ shifts the left and right boundaries of the bistable zone rightward, but the shifting asymptotically comes to an end at very high *k*_4*c*_ values without losing bistability (Figure 7G, left panel). Bistability is lost when *k*_4*c*_ decreases to zero (Figure 7G, left panel inset). Varying *SRX_tot_* has a similar effect to varying *k*_4*c*_, but bistability is lost at high *SRX_tot_* values as well as when it drops to zero (Figure 7H).

Increasing *k*_5_, the non-PRX-mediated *H*_2_*O*_2_ elimination, leads to a parallel upward shift of *Flux_k_*_5_ and consequently an up and leftward shift in the trough of the *Flux_total_* curve without affecting the peak (Figure 7I, right panel). However, eventually, the S shape of the *Flux_total_* curve disappears, resulting in a gradual loss of the bistable zone (Figure 7I, left panel). Decreasing *k*_5_ expands the bistable zone with a leftward shifting boundary and increases *bistability magnitude* (Figure 7I, left panel inset). Increasing *PRX_tot_* leads to a parallel upward shift in the ascending phase and thus the peak of *Flux_k_*_1_, and consequently an up and leftward shift in the peak of the *Flux_total_* curve without affecting the trough much (Figure 7J, right panel). This results in an expanding bistable zone on the right boundary initially as *PRX_tot_* increases and loss of bistability when *PRX_tot_* is too low (Figure 7J, left panel).

#### 3.2.5. Dynamical Responses to Varying H_2_O_2_ Production Rate

We next examined how fast the bistable PTRS system responds to changes in *H*_2_*O*_2_ production rate. As *k*_0_ is stepped up from a basal 10 µM/s to higher values but still below *threshold_ON_* at 152.3 µM/s, most species reach steady states fast (Figure S5). When *k*_0_ exceeds *threshold_ON_*, the switching to the new steady state takes a much longer time, i.e., many hours, to complete, depending on the *k*_0_ value. Even when *k*_0_ increases to 200 µM/s, which is considered to be a very high *H*_2_*O*_2_ production rate, it still takes nearly 10 h to reach steady state. At supra-*threshold_ON_ k*_0_ values, *PRXSOH*, *PRXSS*, and *TRXSS* first switch up to high levels and then switch down to much lower levels (Figure S5C,D,F). This is due to fast sulfenylation of *PRXSH* to *PRXSOH*, followed by slow sulfinylation that converts most *PRXSOH* to *PRXSO*_2_*H*. As *k*_0_ is stepped back to 10 µM/s, the activated state of the PTRS system turns off quickly if the previous *k*_0_ is sub-*threshold_ON_*. However, if the previous *k*_0_ is supra-*threshold_ON_*, the PTRS system turns off much more slowly, taking about 19 h for *H*_2_*O*_2_ and *PRXSH*, 13 h for *PRXSOH*, *PRXSS*, and *TRXSS*, and 26 h for *PRXSO*_2_*H* to reach steady state. *PRXSOH*, *PRXSS*, and *TRXSS* spike down first before they rise. Taken together, the speed of the bistable switching is not conducive to rapid (seconds to minutes) redox signal transduction. The hours-long switching time suggests that the bistable switch may be more suitable for longer time-scale events, such as circadian oscillation of *H*_2_*O*_2_ and *PRXSO*_2_*H*.

### 3.3. PRX Isoform-Specific Responses of the PTRS System

The analyses in the two sections above indicated that a PTRS system, in general, can exhibit complex nonlinear dynamics, with intrinsic ultrasensitivity and possibly bistability. However, different isoforms of PRX may have different tendencies for certain dynamic behaviors because they can have vastly different, as detailed in Table S1 footnote, rate constants in the respective CSSC, including *k*_1_, *k*_2*a*_, *k*_2*b*_, and *k*_3_ [27,31,71,72,73,74,75,76,77,78,79,80,81,82,83]. PRX1, 2, and 3 have the most reported parameter values than other isoforms in the primary experimental literature; therefore, in this section, we explored the specific responses of these three PTRS systems. Given that the protein abundance of a PRX isoform is cell-specific [31,45,74,84,85], we first conducted the analysis by keeping the abundance of each isoform the same, at 100 µM, to reveal their response differences due solely to the differences in the rate constants.

**Figure 8:**
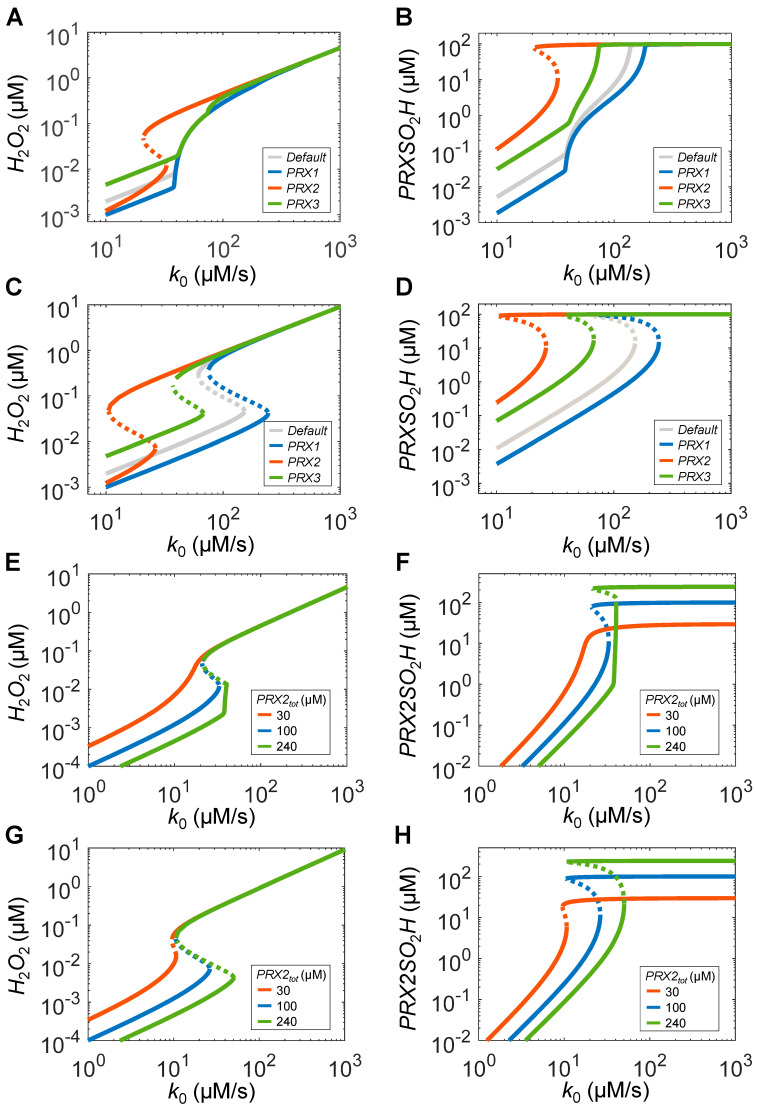
**PRX isoform-specific responses of the PTRS system.** (**A**,**B**) Steady-state responses of *H*_2_*O*_2_ and *PRXSO*_2_*H* respectively with respect to *k*_0_ for different PRX isoforms as indicated using parameter values specific to the Ultrasensitivity Model in Table S1 except for isoform-specific values as indicated below. (**C**,**D**) Steady-state saddle–node bifurcation of *H*_2_*O*_2_ and *PRXSO*_2_*H* respectively with respect to *k*_0_ for different PRX isoforms as indicated using parameter values specific to the Bistability Model in Table S1 except for isoform-specific values as indicated below. (**E**,**F**) Steady-state responses of *H*_2_*O*_2_ and *PRX2SO*_2_*H*, respectively, with respect to *k*_0_ for different total PRX2 as indicated using parameter values specific to the Ultrasensitivity Model in Table S1, except for isoform-specific values as indicated below. (**G**,**H**) Steady-state saddle–node bifurcation of *H*_2_*O*_2_ and *PRX2SO*_2_*H* respectively with respect to *k*_0_ for different total PRX2 as indicated using parameter values specific to the Bistability Model in Table S1, except for isoform-specific values as indicated below. Solid line: stable steady state; dashed line: unstable steady state. Isoform-specific parameter values: for PRX1, *k*_1_ = 100 µM^−1^s^−1^, *k*_2*a*_ = 11 s^−1^, *k*_2*b*_ = 2.2 µM^−1^s^−1^, *k*_3_ = 0.0015 µM^−1^s^−1^; for PRX2, *k*_1_ = 100 µM^−1^s^−1^, *k*_2*a*_ = 0.5 s^−1^, *k*_2*b*_ = 0.61 µM^−1^s^−1^, *k*_3_ = 0.0034 µM^−1^s^−1^; for PRX3, *k*_1_ = 30 µM^−1^s^−1^, *k*_2*a*_ = 22 s^−1^, *k*_2*b*_ = 1 µM^−1^s^−1^, *k*_3_ = 0.012 µM^−1^s^−1^. Note there are no reported values for *k*_2*b*_ of PRX3, but using a wide range of values (0.2–10 µM^−1^s^−1^) barely altered the responses of the P3TRS system in (**A**–**D**).

With the parameter setting (*k*_2*c*_, *k*_5_, *TR_tot_*, and *SRX_tot_*) favoring ultrasensitivity as in Section 1, both the P1TRS and P3TRS systems exhibit similar ultrasensitivity to the default condition, albeit starting at different *H*_2_*O*_2_ and *PRXSO*_2_*H* levels for low *k*_0_ values (Figure 8A,B). In contrast, the P2TRS system becomes bistable with *threshold_OFF_* and *threshold_ON_* of *k*_0_ lower than the *k*_0_ values corresponding to *LRC_max_* for the ultrasensitive responses. With the parameter setting (*k*_2*c*_, *k*_5_, *TR_tot_*, and *SRX_tot_*) favoring bistability as in Section 2, all three PTRS systems still exhibit bistability, but with the bistable zone shifted compared with the default condition (Figure 8C,D). Specifically, the bistable zone of P1TRS shifts to a slightly higher *k*_0_ range with the zone widened, that of P3TRS to an approximately 2-fold lower *k*_0_ range with the zone slightly narrowed, and that of P2TRS to an approximately 6-fold lower *k*_0_ range starting with lower *H*_2_*O*_2_ and higher *PRXSO*_2_*H* levels.

Therefore, PRX2 appears to be more likely to be bistable. However, whether a PTRS system can be bistable is also dependent on the abundance of PRX, with higher abundance favoring bistability and vice versa (Figure 7J). The intracellular PRX2 abundance varies in different cell types and appears to be mostly at sub-100 µM concentrations, although it can be higher in RBCs [31,45,74]. With the parameter setting favoring ultrasensitivity as in Section 1, bistability of P2TRS is totally lost when PRX2 abundance is lowered to 30 µM, but is retained with enhanced ultrasensitivity before the saddle–node bifurcation when PRX2 abundance is 240 µM (Figure 8E,F). With the parameter setting favoring bistability as in Section 2, the bistability of P2TRS is tremendously diminished and almost gone when PRX2 abundance is lowered to 30 µM, whereas the bistable zone is further expanded when PRX2 abundance is increased to 240 µM (Figure 8G,H).

### 3.4. Circadian Oscillation Model

Mitochondrial sulfinylated PRX3 and SRX have been shown to exhibit circadian rhythm [7,8,57]. This oscillatory behavior is believed to involve a global NFL mediated by mitochondrial H_2_O_2_ diffusion into the cytosol, followed by oxidation and translocation of cytosolic SRX into the mitochondrion (Figure 1C). In this section, we set out to explore when the PTRS system is parameterized with available PRX3-specific rate constant values, referred to as the P3TRS module here, and whether it is conducive to generating sustained oscillation when embedded in the global NFL. This involves modifying three parameters from default values to values known for human PRX3 to the best of our knowledge (for details See Table S1 and its footnote), i.e., *k*_1_ from 50 to 20 µM^−1^s^−1^, *k*_2*a*_ from 10 to 22 s^−1^, and *k*_3_ from 0.002 to 0.012 µM^−1^s^−1^ [75,78,83]. In addition, the P3TRS module also includes the *k*_6_ and *k*_7_ steps since both affect *H*_2_*O*_2_ (Figure 1C). In theory, besides an NFL structure, signal amplification is also required to be embedded in the loop for sustained oscillation [19,86]. In the absence of any additional source of ultrasensitivity known to the NFL, the task of signal amplification would rely on the P3TRS module. However, this would require a redox signaling flow from *SRX* to *PRX*3*SO*_2_*H* to mitochondrial *H*_2_*O*_2_ and then to cytosolic *H*_2_*O*_2_. Therefore, we first examined the dynamic behaviors of the P3TRS module per se with *SRX* and *H*_2_*O*_2_ as the respective input and output signals.

**Figure 9:**
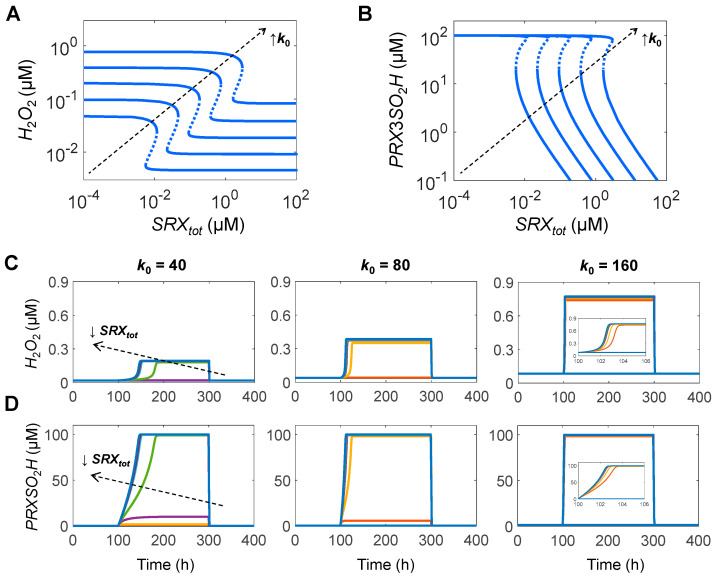
Steady-state and dynamical responses of the P3TRS module to varying *SRX* with parameter setting the same as the Bistability Model in Section 2 except *k*_1_ = 20 µM^−1^s^−1^, *k*_2*a*_ = 22 s^−1^, and *k*_3_ = 0.012 µM^−1^s^−1^ as well as *k*_6*a*_ = *k*_6*b*_ = 100 s^−1^ and *k*_7_ = 3000 s^−1^. (**A**,**B**) Saddle–node bifurcation of steady-state levels of mitochondrial *H*_2_*O*_2_ and *PRX3SO*_2_*H* respectively with respect to *SRX_tot_* under different *k*_0_ conditions (10, 20, 40, 80, 160 µM/s). Solid line: stable steady state; dashed line: unstable steady state. (**C**,**D**) Time–course responses of mitochondrial *H*_2_*O*_2_ and *PRX3SO*_2_*H* respectively to varying *SRX_tot_* values under different *k*_0_ conditions (µM/s) as indicated. The P3TRS module is at a steady state first from 0 to 100 h with *SRX_tot_* = 5 µM. *SRX_tot_* is then stepped down to different levels (5, 0.5, 0.25, 0.1, 0.05, 0.01, 0.005, 0 µM) between 100 and 300 h, after which it is stepped up to 5 µM. Dashed arrows indicate the direction of change of the parameter values as indicated.

Since the PTRS system is capable of both ultrasensitivity and bistability, we explored the behaviors of the P3TRS module by using the two-parameter settings (except for the PRX3-specific *k*_1_, *k*_2*a*_ and *k*_3_) corresponding to the Ultrasensitivity Model and Bistability Model in Section 1 and Section 2, respectively. Our simulations showed that for the parameter setting associated with the Ultrasensitivity Model, *H*_2_*O*_2_ and *PRX*3*SO*_2_*H* exhibit ultrasensitivity when *k*_0_ is >66 µM/s and bistability when *k*_0_ < 66 µM/s in response to varying *SRX_tot_* (Figure S6A,B). The fold-change of *H*_2_*O*_2_ in the ultrasensitive responses is, however, quite small, <2. Time–course simulations showed that *SRX*-driven switching occurs on a time scale of hours (Figure S6C,D). At low *k*_0_ values when the P3TRS module is bistable, such as 40 µM/s, it can take minimally 63 h to turn on the switch by decreasing *SRX_tot_*. At higher *k*_0_ values where the P3TRS module is ultrasensitive only, the activation time is considerably shorter but still on the scale of hours. For instance, at *k*_0_ = 80 µM/s, it takes as short as 13 h to switch on, and at *k*_0_ = 160 µM/s, 4.5 h. In contrast, the switching-off time is insensitive to *k*_0_, and the P3TRS module can be turned off in 1.2 h by increasing *SRX_tot_*. For the parameter setting associated with the Bistability Model, *H*_2_*O*_2_ and *PRX*3*SO*_2_*H* exhibit bistability for the entire range of *k*_0_ (10–160 µM/s) (Figure 9A,B). Time–course simulations showed that *SRX*-driven bistable switching occurs on a time scale of hours (Figure 9C,D). At low *k*_0_ values, such as 40 µM/s, it can take minimally 50 h to turn on the switch by decreasing *SRX_tot_*. At higher *k*_0_ values, the activation time is considerably shorter but still on the scale of hours. For instance, at *k*_0_ = 80 µM/s, it takes as short as 12 h to switch on, and at *k*_0_ = 160 µM/s, 3 h. In contrast, the switching-off time is insensitive to *k*_0_, and the system can be turned off in less than 2 h by increasing *SRX_tot_*.

These simulation results indicate that the *SRX*-driven switching of *H*_2_*O*_2_ and *PRX*3*SO*_2_*H*, regardless of operating in the bistability or ultrasensitivity mode, does not fit for instant signaling, but is compatible with long-time-scale events such as circadian oscillation. The fold-change in the *H*_2_*O*_2_ response in the ultrasensitivity mode is quite small (Figure S6A), posing a challenge to provide sufficient amplification to produce sustained oscillations of reasonable amplitude in the absence of any other amplification sources. Therefore, we went on to use the parameter setting of the Bistability Model (i.e., *k*_1_, *k*_2*a*_, and *k*_3_) for the oscillation model.

**Figure 10:**
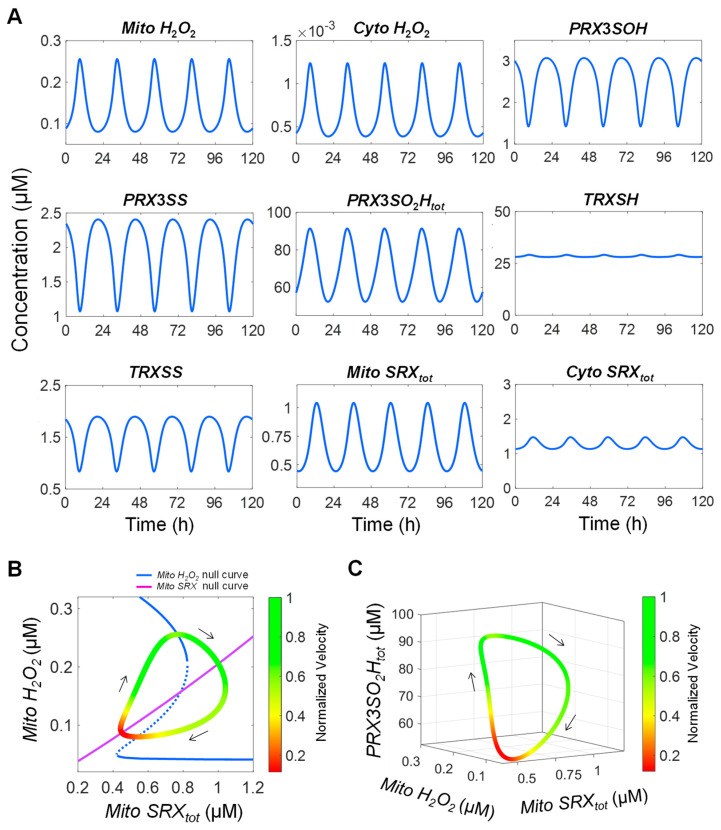
**Sustained 24 h oscillation of H_2_O_2_, PRX3, TRX, and SRX species of the oscillation model.** (**A**) Time–course of sustained oscillation for variables as indicated. (**B**) Null curves of *Mito H*_2_*O*_2_ and *Mito SRX_tot_* as indicated overlaid with 2-D trajectory with normalized velocity. The normalized velocity was defined by first calculating the square root of the sum of the squared percentage changes in *Mito H*_2_*O*_2_ and *Mito SRX_tot_*, relative to their peak values, every 10 s, and then normalizing these square root values to the maximum square root value (see MATLAB code for details). (**C**) The 3-D trajectory of *Mito H*_2_*O*_2_, *Mito SRX_tot_*, and *PRXSO*_2_*H_tot_* with normalized velocity was calculated similarly as in (**B**). Arrows in (**B**,**C**) indicate the direction of movement.

With appropriate parameterization of the cytosolic processes, the *SRX*-mediated NFL can indeed produce sustained oscillation of 24 h period (Figure 10A). At *k*_0_ = 84.2 µM/s, mitochondrial (*Mito*) and cytosolic (*Cyto*) *H*_2_*O*_2_ oscillate in phase with pulses that can reach peak levels near 0.26 and 0.00124 µM and trough levels near 0.08 and 0.0004 µM, respectively. *PRX3SO*_2_*H_tot_* oscillates with a peak level that can reach over 90 µM and a trough level near 50 µM. *PRX3SOH* and *PRX3SS* oscillate at low µM levels with peak/trough ratios between 2:1 and 3:1. TRX is predominantly in the reduced state with *TRXSS* oscillating at low µM levels. *Mito SRX_tot_*, including free *SRX* and *PRX3SO*_2_*H:SRX*, oscillates with the peak reaching 1 µM and trough down to 0.44 µM. In comparison, *Cyto SRX_tot_* oscillates around 1.3 µM with a much smaller peak/trough ratio, consistent with the experimental observation that mitochondrial SRX oscillates while cytosolic SRX exhibits minimal oscillation [8].

The two null curves, *Mito SRX_tot_* and *Mito H*_2_*O*_2_, which can be regarded as the respective steady-state input–output relationships of the two arms of the NFL, (i) *Mito H*_2_*O*_2_ → *Cyto H*_2_*O*_2_ → oxidized *Cyto SRX* → *Mito SRX_tot_* and (ii) *Mito SRX_tot_* ┤ (inhibits) *PRX3SO*_2_*H_tot_* → *Mito H*_2_*O*_2_, are plotted together in Figure 10B. They intersect only once, at an unstable steady state of the *Mito H*_2_*O*_2_ null curve. When the 2-D trajectory of *Mito H*_2_*O*_2_ and *Mito SRX_tot_* are overlaid, it becomes obvious that the trajectory loops around the *Mito H*_2_*O*_2_ null curve. The normalized velocity (see Figure 10B legend for definition), as indicated by the color of the trajectory, varies dramatically depending on the position of the trajectory relative to the S-shaped *Mito H*_2_*O*_2_ null curve. The slowest points are clustered around the corner when *Mito H*_2_*O*_2_ is about to increase with *Mito SRX_tot_* decreasing toward the lowest levels (Figure 10B, red segment at bottom left corner). This slowness is consistent with the relatively long *H*_2_*O*_2_ initial take-off time indicated in Figure 9C in response to decreasing *SRX_tot_*. The fastest points are when *Mito H*_2_*O*_2_ is on its way to spiking up (Figure 10B, green segment). When the 3-D trajectory of *Mito H*_2_*O*_2_, *Mito SRX_tot_*, and *PRXSO*_2_*H_tot_* is plotted, the slowest and fastest segments remain relatively in the same locations (Figure 10C).

**Figure 11:**
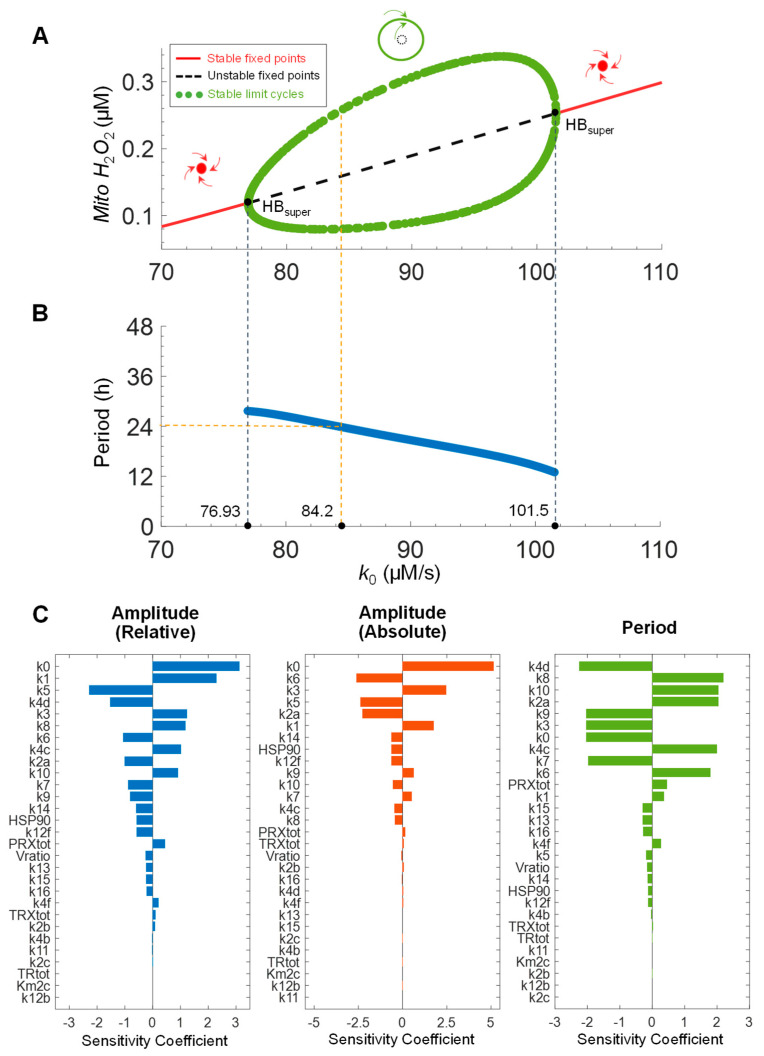
**Bifurcation and sensitivity analyses of the oscillation model.** (**A**) One-parameter bifurcation analysis for *Mito H*_2_*O*_2_ with respect to *k*_0_. Symbol denotations are as indicated, HB_super_: supercritical Hopf bifurcation. Red dots with arrows or green circles with arrows on the top illustrate the stability behaviors corresponding to the respective segments underneath. (**B**) One-parameter bifurcation analysis for the oscillation period of *Mito H*_2_*O*_2_ with respect to *k*_0_. Vertical orange dashed line connecting (**A**,**B**) indicates the default *k*_0_ value and black dashed lines indicate *k*_0_ values associated with the two HB_super_ points. (**C**) Local sensitivity analysis for the relative and absolute amplitude and period of *Mito H*_2_*O*_2_ oscillation.

One-parameter bifurcation analysis showed that as *k*_0_ varies between 76.93 and 101.5 µM/s, stable limit cycles emerge as supercritical Hopf bifurcations (Figure 11A). Here, a stable limit cycle refers to stable periodic oscillation, and supercritical Hopf bifurcation describes a phenomenon where as a nonlinear system’s parameter is varied, the system, which was at a stable steady state, starts to oscillate with a small amplitude. The oscillation amplitude first increases and then decreases as *k*_0_ increases. The oscillation period decreases almost linearly as *k*_0_ increases (Figure 11B). To further explore how the oscillation behaviors depend on the parameters, we conducted a local sensitivity analysis for the amplitude and period of *Mito H*_2_*O*_2_ pulses (Figure 11C). For the relative amplitude (defined as peak/trough ratio), besides some cytosolic parameters (*k*_8_, *k*_10_, *k*_7_, *k*_9_), mitochondrial parameters *k*_0_, *k*_1_, *k*_5_, *k*_4*d*_, and *k*_3_ are among the most sensitive. For the absolute amplitude (defined as the difference between peak and trough), *k*_0_, *k*_6_, *k*_3_, *k*_5_, *k*_2*a*_, and *k*_1_ are the most sensitive parameters. For the oscillation period, a number of cytosolic and mitochondrial parameters are among the sensitive ones.

## 4. Discussion

The CSSC of the PTRS system is capable of underpinning a rich array of signaling dynamics of H_2_O_2_, PRX, and TRX, including linearity, ultrasensitivity, bistability, and nonmonotonicity [30,31,32]. In the present study, we first explored in-depth the origins of ultrasensitivity driven by H_2_O_2_, which can be attributed to the saturable nature of the redox cycles. We then analyzed the ultrasensitivity in the two arms of the PFL and the parameter conditions that render bistability. Lastly, we showed that the P3TRS system operating in the mitochondrion can provide the necessary signal amplification to enable supercritical Hopf bifurcation when embedded in a global NFL involving H_2_O_2_-stimulated SRX oxidation and mitochondrial translocation, a circuit that is believed to underpin the circadian rhythm of PRX3 sulfinylation in several mammalian cell types [7,8,57]. Our contribution to the field here is to reveal the multiple sources of ultrasensitivity intrinsic to the CSSC of PRX and their mechanisms, with added kinetic details to the sulfinylation cycle. Our study extends the earlier work in these areas and provides key insights into the signal amplification scheme in the PTRS system, which renders nonlinear dynamics in redox signaling.

### 4.1. Origins of Ultrasensitivity

Our analysis showed that the sulfenylation cycle is intrinsically ultrasensitive through zero-order reactions, a mechanism that can produce strong ultrasensitivity [70,87]. Specifically, it can be attributed to (i) the TR-mediated reduction in TRXSS to TRXSH that is for the most part near saturation due to the low Michaelis constant *K_m_*_2*c*_ and total TR relative to the high concentration of total TRX in mammalian cells (for value details see Table S1) [31,74,88,89,90,91,92,93], and (ii) also the capped capacity of oxidization of TRXSH to TRXSS by PRXSS, which is limited by total PRX. Both work collectively to generate the zero-order ultrasensitivity exhibited by TRXSS and PRXSS in response to H_2_O_2_ and the ultrasensitivity of H_2_O_2_ itself in response to increasing production of H_2_O_2_. This ultrasensitivity does not involve the sulfinylation cycle, requiring minimal PRX hyperoxidation.

The sulfinylation cycle itself is also intrinsically ultrasensitive to H_2_O_2_ as an input, due, in part, to a similar zero-order mechanism. Specifically, the concentration of cytosolic SRX is usually at very low µM, but the concentrations of various types of PRX can be in the order of hundreds of µM [31,45,74,94]. Moreover, the available Michaelis constant value for the SRX-catalyzed reduction of PRXSO_2_H is < 10 µM [8,95]. This combination of parameter conditions favors the saturation of SRX by PRXSO_2_H. In addition, H_2_O_2_ promotes the production of PRXSO_2_H by participating as a reactant in both (i) the step where PRXSOH is formed through the sulfenylation reaction; and (ii) the subsequent step where PRXSO_2_H is formed through the sulfinylation reaction. This sequential participation of H_2_O_2_ also generates ultrasensitivity, similar to the multistep signaling situation where the MAPK kinase (MAPKK) catalyzes the dual phosphorylation of MAPK in a non-processive manner, which contributes to the canonical ultrasensitive response observed in the MAPK cascade [96]. The difference is that in the current model the input H_2_O_2_ is consumed while MAPKK is not.

### 4.2. Differential Signaling Modality of PRX and TRX Intermediates

The fast reaction with and thus elimination of H_2_O_2_ by PRX and potentially by other antioxidant enzymes create a redox signaling conundrum. At only 10–100 M^−1^s^−1^, the rate constants of the second-order reactions between H_2_O_2_ and the target signaling proteins, such as PTP1B and SHP-2, are 5–6 orders of magnitude lower than those between H_2_O_2_ and PRX, GPx, and CAT, which range between 10^5^–10^8^ M^−1^s^−1^ [1,41,72,74,75]. For oxidation of the target proteins to occur fast enough as cell signaling events, i.e., in seconds to minutes rather than hours, and to reach sufficiently high levels, the intracellular H_2_O_2_ concentration needs to reach 100 µM or higher. Yet, intracellular H_2_O_2_ at low µM can be already cytotoxic [97] and much lower apoptosis-inducing concentrations were even suggested [98]. This contrasts with the basal steady-state levels of H_2_O_2_ in the cytosol, which are believed to be in the low 1–10 nM range or lower [99]; and it was estimated that it can rise only to the 500–700 nM level transiently during oxidative signaling [100].

This concentration mismatch suggests that direct oxidation of target proteins by H_2_O_2_ may not be conducive to redox signal transduction, and cells utilize other strategies, including H_2_O_2_ hotspots to boost local concentrations and/or signaling relay via intermediate redox proteins [101,102]. Signaling relay through intermediate molecules such as oxidized PRX, GPx, TRX, or bicarbonate has been demonstrated in many studies [46,47,48,49,50,51,52,103,104,105]. For instance, in human cells oxidized PRX1 and 2 can relay the H_2_O_2_ signal to a wide spectrum of proteins by forming transient disulfide bonds with them, a process that does not require TRX and TR. More recently using a mass spectrometry-based approach it was shown that human PRX1-5 can form disulfide-dependent heterodimers with hundreds of proteins, with each isoform displaying a differential preference for target proteins [106]. In yeast cells, light stimulates, via Pox1, a conserved peroxisomal oxidase, the production of H_2_O_2_; in turn, H_2_O_2_ drives the oxidation of Tsa1 which then relays the redox signal to TRX to inhibit PKA activity to regulate the nuclear exportation of Msn2 [107].

Differentially parameterized PTRS systems may utilize different intermediates for redox signaling relays. When the resolution step is rate-limiting, PRXSOH accumulates; when the reduction step is rate-limiting, PRXSS accumulates, and when the TR step is rate-limiting, TRXSS accumulates. For instance, the resolution step of human PRX2 can be as much as 50 times slower than that of PRX1, suggesting that PRX2SOH and PRX1SS may be used as the redox signaling intermediates, respectively [108]. Cellular signaling requires more than simple linear responses as signal amplification through ultrasensitivity is required for a variety of cellular functions [19]. Our model and others [31] revealed that signaling relay through PRXSOH, PRXSS, and TRXSS can be linear or ultrasensitive with drastically different degrees of amplification, suggesting that quantitatively different signal transduction modalities exist to accommodate a wide variety of redox regulation needs. The nonmonotonic responses of PRXSOH, PRXSS, TRXSH, and TRXSS, resulting from hyperoxidation of PRX, further highlight the complexity of redox signaling under different oxidative conditions. The reversal of TRXSH at high *k*_0_ values is consistent with the experimentally observed increases in the reduced state of TRX and cellular proteins when cells are oxidatively stressed severely [40,109,110].

### 4.3. Cellular Parameter Conditions for Bistability

The PTRS system has the potential to be bistable due to the existence of the PFL between H_2_O_2_ and PRXSO_2_H and the intrinsic ultrasensitivity embedded in the two arms of the PFL. As mentioned above, the H_2_O_2_ → PRXSO_2_H arm is ultrasensitive because of the zero-order sulfinylation-desulfinylation cycle and the 2-step formation of PRXSO_2_H from PRXSH through PRXSOH simultaneously driven by H_2_O_2_. Therefore, the sulfinylation of PRXSOH to PRXSO_2_H by H_2_O_2_ plays a dual role—it closes the PFL and adds some additional degree of ultrasensitivity to the feedback loop. Our analysis showed that the PRXSO_2_H → H_2_O_2_ arm is also ultrasensitive when PRXSO_2_H approaches the total PRX abundance because of the consequent dramatic percentage decrease in the abundance of non-PRXSO_2_H species under the condition that H_2_O_2_ is eliminated predominantly by PRX relative to other elimination routes. This manner of ultrasensitivity generation does not seem to conform to any of the known ultrasensitive response motifs or their variants [19,65,111], thus representing a potentially novel mechanism.

As revealed in our two-parameter bifurcation analysis, the size and magnitude of the bistable zone can be influenced by many parameters. Salvador and colleagues had conducted in-depth studies with parameter conditions belonging to different cell types and suggested that bistability is a relatively rare phenomenon of the PRX1 and two systems, although its occurrence cannot be ruled out in some cell types [31,32]. The early version of their models [30] favors bistability due to two approximations: (i) no alternative H_2_O_2_ sink (equivalent to *k*_5_ = 0 in our model); and (ii) no TRX oxidation–reduction cycle, despite the reduction in TRXSS, is a TR-mediated, saturable reaction thus potentially rate-limiting [32]. Adding back these reactions, especially when the V_max_ of the reduction in TRXSS is low, bistability becomes less likely to occur or vanishes [31]. Intuitively, when the reduction in TRXSS by TR becomes rate-limiting, TRXSH is low, and PRXSS accumulates; thus, less PRXSOH is available for sulfinylation, which disfavors bistability. Our flux analysis further corroborated this by showing that lowering V_max_ by significantly decreasing *k*_2*c*_ or *TR_tot_* causes a lowering and flattening of the peak of the *Flux_k_*_1_ curve, consequently diminishing the S-shape of the *Flux_total_* curve (Figure S4A,D), which leads to diminished and eventually loss of bistability.

Mathematical models by Salvador et al. and others have used pseudo-first-order kinetics to describe the reduction of PRXSO_2_H by SRX [30,31,32]. Absent other information, this was partially justified by an inferred Michaelis constant of about 20 µM between yeast SRX and Tsa1 [31,112]. The Michaelis constants of mammalian sulfinylated PRX1 and 2 reductions are unknown. Using SRX in the mouse liver mitochondrial and cytosolic fractions and sulfinylated PRX3 as substrate, the measured association rate constant is 0.0014 µM^−1^s^−1^ and the dissociation rate constant is 0.001 s^−1^, resulting in a *K_d_* = 0.71 µM [8]. The measured *K_d_* for the binding between human PRX4 and SRX is 4.0–7.0 µM [95]. The SRX concentrations are usually at very low µM in contrast to the much higher (up to hundreds of µM) PRX concentrations [45,84,85,94,113]. Furthermore, as demonstrated in yeast with Tsa1 decamer, PRXSH may bind with and thus sequester SRX away from its substrate PRXSO_2_H, further reducing the availability of SRX [114]. Taken together, these reported parameter settings prompt us to suspect that in some circumstances it may not take much PRXSO_2_H to drive the SRX-mediated reaction out of the linear range and into saturation; thus, an explicit treatment of the enzymatic kinetics is probably warranted to describe this reaction than pseudo-first-order kinetics (for details, see Table S1 footnote). As shown in the flux analysis, this saturable reaction as implemented here provides a source of zero-order ultrasensitivity of H_2_O_2_-stimulated PRXSO_2_H production (Figure 3E), which would facilitate bistability. Increasing *k*_4*f*_ and decreasing *k*_4*b*_, which decreases the apparent Michaelis constant, expands the bistability zone and its right *k*_0_ border without affecting the *bistability magnitude* much (Figure 7E,F). Decreasing *k*_4*c*_, which decreases V_max_ and also to a small extent the Michaelis constant, or decreasing *SRX_tot_* shifts the bistable zone to a lower *k*_0_ range with enhanced *bistability magnitude* (Figure 7G,H).

Despite sharing a common structure of CSSC, different isoforms of PRX have very different parameter values for the CSSC and are expressed at different abundances in different cell types. Based on our two-parameter bifurcation analysis (Figure 7), bistability seems to be more favored at higher *k*_1_, lower *k*_2*a*_, higher *k*_2*b*_, higher *k*_3_, and higher *PRX_tot_* values, with each parameter having different sensitivities around the default values. The propensity of a PRX isoform for bistability is determined by the combined setting of these isoform-specific parameters and cellular conditions. Among the three PRX isoforms we explored, PRX2 seems more readily to exhibit bistability than PRX1 and 3 (Figure 8A–D). This prediction is consistent with experimental observations that PRX2 is generally more prone to hyperoxidation than PRX1 and 3 [27,45,71,78,83]. Comparing the parameter values between the three isoforms, it appears that PRX2 has higher *k*_1_, lower *k*_2*a*_, lower *k*_2*b*_, and intermediate *k*_3_ values compared with PRX1 and 3 [27,31,71,72,73,74,75,78,79,80,81,82,83]. Thus, for PRX2, its *k*_1_ and *k*_2*a*_ values favor while its *k*_2*b*_ value disfavors bistability. *k*_2*a*_ is likely the main contributor here among the four as its value for PRX2 is more than 20-folder but lower than PRX1 and 3, and the bistable zone shifts sensitively to lower *k*_0_ values as *k*_2*a*_ decreases (Figure 7B). It is worth noting that on the log–log scale, the leftward shifting of the left and right borders of the bistable zone is almost parallel to each other within a wide range of *k*_2*a*_, thus in the fold-change term, the bistable zone is not “shrinking” as it appears on the linear-linear scale. Although the *k*_2*b*_ value of PRX2 can be as much as 10-fold lower than those of PRX1 and 3, it is a relatively insensitive parameter as lowering its value by 10-fold from the default value only shifts the right border of the bistable zone to a *k*_0_ value about 25% lower than the default condition (Figure 7C). Lastly, whether PRX2 can exhibit bistability in a cell also depends on its abundance as when PRX drops below 30 µM, bistability may just disappear (Figure 8G,H).

It is worth noting that the above analysis was performed with each PRX isoform system modeled in isolation, while multiple isoforms can coexist in the same subcellular compartment in cells, for instance, PRX1 and 2 in the cytosol. The differential switching threshold of each PTRS system suggests that the overall response could be staggered [72]. The strong H_2_O_2_-degrading capacity of one PRX isoform is equivalent to substantially increasing *k*_5_, the alternative H_2_O_2_ path, for another PRX isoform system, which will reduce the possibility of bistability. In the most recent study by Griffith et al., where both PRX1 and 2 were simultaneously modeled, only ultrasensitivity and no bistability were predicted [32]. Lastly, according to the simulations by us and others, the H_2_O_2_ production rate that is required to elicit ultrasensitive or bistable responses is generally in a high range of up to over 100 µM/s. While this may be attainable under severe oxidative stress or in experimental conditions with high exogenous H_2_O_2_ concentrations, whether ultrasensitivity and especially bistability occur in vivo, where the physiological H_2_O_2_ production rate tends to be more moderate, remains to be determined.

### 4.4. Existing Models of Switch-like Redox Responses

Many mathematical modeling studies have been published to investigate switch-like redox signaling and responses and potential mechanisms. A model of the coupled TRX and TPx redox cycle in *E. coli* showed the potential to generate ultrasensitivity in reduced TRX and related fluxes in response to TR [33,34,35]. The mechanism could be a variant of zero-order ultrasensitivity, where the reduction in TRX by TR was described as a Michaelis–Menten reaction, and its oxidation by TPx, which itself is oxidized by H_2_O_2_, was treated as a mass action. In yeast cells, to explain the observed biphasic increases in intracellular H_2_O_2_ and sulfinylated TPx1 in response to extracellular H_2_O_2_, mathematical modeling suggested that it was due to saturation of the H_2_O_2_-metabolizing capacity comprising enzymes and protein thiols other than the TPx1 system [20]. A model of the PRX2 system in RBC showed that under the condition that PRX2 is abundant and readily available to react with H_2_O_2_, H_2_O_2,_ and PRXSO_2_H can exhibit bistable switch-like responses to increasing H_2_O_2_ supply rate; however experimental evidence supports that it is more likely that most of PRX2 is held in an inhibited form by associating with other proteins which may include hemoglobulin, and as a result the H_2_O_2_ response is mostly graded but ultrasensitive at a higher H_2_O_2_ supply rate [36]. In a more recent study modeling the mitochondrial PRX3 network in HeLa cells, an abrupt dynamic switching of H_2_O_2_ and PRX3SO_2_H was predicted when the mitochondrial H_2_O_2_ production rate was increased to nearly 54 µM/s; however, experimental findings in the same study did not support the switching behaviors when the cells were challenged with increased mitochondrial H_2_O_2_ production [37]. Salvador and colleagues revealed the theoretical existence of bistability in the PRTS system but concluded that it is unlikely to be common [30,31,32]. Among the many cell types they explored, Jurkat T cells may most likely show bistability for composite PRX1 and 2 but only with a very narrow bistable zone pending parameter uncertainties [31]. Interestingly, experimental studies are suggestive of bistability in Jurkat cells in which sulfinylated PRX1 and 2 exhibited highly switch-like responses when exogenously added H_2_O_2_ increased from 10 to 20 µM, while the response in HeLa cells was more graded [45]. However, without showing hysteresis whether the switch-like response represents true bistability in Jurkat cells remains to be determined.

Besides the PTRS system, switch-like responses appear to be an emergent property in other redox systems. In a study on the coupling of the nutrient metabolic and ROS signaling, it was demonstrated in T98, a glucose-addicted cell line, that ROS, particularly H_2_O_2_, can be toggled between two distinct states with hysteresis by controlling the availability of glucose [23]. A mathematical model suggested that a DNFL between glutathione/NADPH and ROS is at the center of the nutrient-redox network underpinning the observed bistability. ROS signal amplification, self-perpetuation, and bistability have also been suggested for multiple PFLs in ROS-dependent YAP-HIF-Notch-(PD-L1) signaling axis, which may underpin decision-making in development, tissue homeostasis, and cancer progression [24].

### 4.5. Circadian Oscillations of the P3TRS System

In our circadian oscillation model, the H_2_O_2_-stimulated SRX oxidation and mitochondrial translocation provide a long, global NFL, while the P3RTS module acts as an embedded PFL. Despite the P3RTS module being parameterized to be bistable in response to SRX in our model (Figure 10B), the stable limit cycle is born, surprisingly, out of supercritical Hopf bifurcation (Figure 11A). This suggests that a resonant-like oscillator is at work, which is consistent with the smooth, sinusoid-like pulses exhibited by nearly all variables (Figure 10A). The lack of relaxation oscillation is likely owing to the long timescale of the H_2_O_2_ response to SRX (Figure 9C), such that in a 24 h timeframe, the P3RTS module may not function as a full bistable switch but instead operates to provide ultrasensitivity.

The basal H_2_O_2_ production rate varies tremendously among different cell types and in different organelles [32,115,116,117,118,119,120,121,122,123]. While it is mostly in the low µM/s range, it can be higher in energetic organelles such as mitochondria. Our bifurcation analysis indicates that oscillation only occurs within a certain range of relatively high H_2_O_2_ production rates (Figure 11A). This result is consistent with the observation that PRX3 and SRX circadian oscillations mainly occur in the mitochondria of the adrenal gland, heart, and brown adipose tissues [8], where cell metabolism is expected to be high compared with many other tissues.

del Olmo et al. published a computational model of circadian oscillation of H_2_O_2_ and PRX3 showing the oscillation is robust within a wide range of parameter variations [63]. However, there are several caveats which question the robustness of the modeled oscillator: (i) The model variables, H_2_O_2_, PRX3, and SRX, are all normalized to arbitrary units, with unity as their maximum values, despite the vast differences in concentrations among these species as we documented in Table S1; (ii) While the physiological values of several key rate constants were provided with second as the time unit, these numbers appeared to be used as is without appropriate conversion to the hourly time unit that was actually used in their model; (iii) The mitochondrial H_2_O_2_ translocation to cytosol was modeled as a unidirectional delayed step (about 5 h), despite that cross-membrane H_2_O_2_ trafficking is expected to be nearly instantaneous and bidirectional. This time delay causes an unrealistic, several-hour phase difference between the mitochondrial and cytosolic H_2_O_2_ pulses in their model; (iv) The reduction in PRXSS to PRXSH was only modeled as a first-order process without the necessary sophistication of TRX and TR participation, which puts a limit to the reduction capacity. The waveforms of the pulses of PRXSOH, PRXSO_2_H, and H_2_O_2_ are triangular in their study, based on which the oscillation was claimed to be relaxation-like. While the authors noted that relaxation oscillations usually require a PFL nested within an NFL, the CSSC was not identified as a source of PFL. Compared with the del Olmo model, our model provides substantial improvement by making several modifications, including using physiological concentrations and parameters, making H_2_O_2_ translocation a non-limiting and bidirectional step, and treating the reduction in PRXSS as saturable TR-mediated reaction and reduction of PRXSO_2_H as saturable SRX-mediated enzymatic kinetics.

### 4.6. Limitations and Future Directions

The present study has several limitations. One underlying assumption for the 2-Cys PRX modeled here is that the two pairs of C_p_ and C_R_ in the homodimer are oxidized and reduced independently of each other. Recently it was demonstrated that positive cooperativity exists for the sulfenylation of the two C_p_ in the PRX2 dimer when exposed to acutely high, not slowly rising, H_2_O_2_, and negative cooperativity exists for the disulfide formation, the first step of the reduction process [79]. These molecular interaction details may modulate the ultrasensitivity of the PTRS system, but we do not expect the model’s behavior will be impacted qualitatively.

One cellular mechanism to protect against protein hyperoxidation is the persulfidation of protein sulfenic acids, by reacting with H_2_S, followed by depersulfidation [124]. Persulfidation has been demonstrated with the C_p_ residue of human PRX2, which is recovered to PRX2SH by depersulfidation via a mechanism involving thioredoxin-related protein of 14 kDa (TRP14) and TR2 [125,126]. Future iterations of our model may consider including these cycles which may modify the nonlinear behavior of the PTRS system.

The present study modeled the PTRS system as in a well-mixed compartment, but it is well known that within a limit of H_2_O_2_ production, steep concentration gradients are established from the vicinity of the H_2_O_2_ source to the distal area in the cytosol and potentially within an organelle such as mitochondria [30,32,127]. With these spatial considerations, where H_2_O_2_, PRX, TRX, and TR can all diffuse with vastly different diffusion constants, the degree of ultrasensitivity and potential bistability may be modulated locally, but ultrasensitivity seems to still occur in the vicinity or distal site of the H_2_O_2_ source, as recently showed in [32].

In our model here, PRX3 is oxidized and hyperoxidized by H_2_O_2_ only, while in the mitochondrion PRX3 can also be readily oxidized and hyperoxidized by fatty acid hydroperoxides and peroxynitrite [128,129]. Given that the formation of these reactive species normally correlates with that of H_2_O_2_ as they all source from superoxide [130,131,132], the actual *k*_1_ and especially *k*_3_ values can be higher than we used here. This may facilitate the ultrasensitivity/switching or oscillation behaviors of the P3TRS system at a lower *k*_0_ rate, which can be explored in future iterations of our models.

In our oscillation model, PRX3 has to be sulfinylated to levels (>50% of total) that can significantly decrease the amount of non-PRX3SO_2_H species to tangibly alter mitochondrial H_2_O_2_ levels. Whether such a high fraction of hyperoxidation occurs in vivo is unclear. The study by Kil et al. used Western blot to determine the oscillating amount of PRX3SO_2_H, which appeared to be significant relative to total PRX3 based on band optical density [8,57]. However, since Western blot is a semi-quantitative technique, the actual fraction of PRX3SO_2_H cannot be determined. Whether it requires >50% of PRX3 to be hyperoxidized, as suggested in our model, remains to be determined. In addition, the oscillating pulses of mitochondrial PRX3SO_2_H appear to be largely in anti-phase with SRX in the study by [8,57]; yet, the two variables in our model only exhibit a phase lag of several hours, suggesting additional mechanisms may operate in cells that were not captured in our model.

One simplification of the oscillation model is the omission of a background cytosolic H_2_O_2_ production which may attenuate the dynamic range of mitochondria-released H_2_O_2_, thus reducing the overall feedback gain. However, because of the reducing activity of PRX1 and PRX2 in the cytosol, mitochondria-released H_2_O_2_ is only expected to be within the cytosolic proximity of the mitochondrial outer membrane [127]. Therefore, the action of SRX oxidation is expected to occur nearby, immediately followed by translocation of oxidized SRX to the mitochondrion. With this spatially focused signaling scenario, cytosol-generated H_2_O_2_ may not have a tangible impact on the local concentrations of mitochondrion-derived H_2_O_2_. In the model, we had to set the trans-mitochondrion H_2_O_2_ translocation constant *k*_6_ to be high to ensure sufficient cytosolic H_2_O_2_ concentrations. If mitochondrion-released H_2_O_2_ can be limited to the vicinity instead of being distributed into the whole cytosol, a much lower *k*_6_ value may be used. These spatial effects can be explored in the future. The mitochondrial translocation of the SRXSSHSP90 complex via TOM is more complicated than we modeled here, which requires protein unfolding and folding [133]. SRX may need to be first unfolded and then, once in the mitochondrion, re-folded and reduced to regain its proper structure and enzymatic activity. Although these events, which are slow, are not explicitly considered in our model, they are lumped into the *k*_14_ step and, thus, implicitly considered. In future iterations, such rate-limiting processes can be more explicitly modeled, which may further help with oscillation given the time delay they introduce.

### 4.7. Perspectives

Under oxidative stress, endogenous antioxidant enzymes can be coordinately upregulated as part of the cytoprotective response mediated by the master transcription factor NRF2 [134,135,136]. Among the inducible antioxidant enzymes relevant to our model are PRX1, 2, and 5 [137,138,139], TRX and TR [140,141,142,143], and SRX [144,145,146,147,148,149]. While in the present study, we investigated the behaviors of the PTRS system at basal conditions where the total abundances of PRX, TRX, and SRX remain constant, it is unclear how their collective upregulation under oxidative stress affects the dynamical behaviors. During oxidative stress responses, the H_2_O_2_ level increases, at least initially, to drive the PTRS responses, but the responses are expected to be altered by subsequent upregulation of PRX, TRX, TR, and SRX. These changes in antioxidant capacity may also affect the amplitude and period of the H_2_O_2_ circadian oscillations. Future modeling in this regard can help understand the disruption of H_2_O_2_ signaling and circadian rhythm by environmental and therapeutic chemicals that either activate NRF2 directly [150,151,152] or indirectly through other molecular initiating events such as activation of aryl hydrocarbon receptors [153,154].

While the oscillation of PRX3 hyperoxidation arises from the SRX-mediated NFL, the circadian rhythm of other PRX isoforms results from different mechanisms, e.g., PRX2 oscillation in RBC appears to involve hemoglobin autoxidation and the 20S proteasome [62]. Regardless of the oscillation mechanisms, these redox clocks do not operate in isolation. They can be entrained by environmental, physiological, and metabolic signals, and even intermingled with the core circadian clock in a variety of species. In cyanobacterium, Synechococcus elongatus PCC7942, the endogenous redox rhythm and the central KaiABC oscillator mutually interact with each other [155]. In mammals, the mitochondrial H_2_O_2_ production can be controlled by the core circadian clock, where CLOCK/BMAL1 transcriptionally regulates the nicotinamide phosphoribosyltransferase (NAMPT) to increase the biosynthesis of NAD^+^, NAD^+^ then activates Sirtuin 3 (SIRT3), a deacetylase, to enhance the Krebs cycle to produce more NADH, and NADH is fed to the mitochondrial respiratory chain to induce more H_2_O_2_ production [156]. It has been recently demonstrated that the redox rhythm and TTFL clock are closely coupled through the diurnal variation in endogenous H_2_O_2_ and p66 to reinforce the circadian oscillation [10]. The coupling of the redox and TTFL oscillators may increase the robustness against noise perturbations [157].

The control of daily oxidative stress due to cell metabolism and the circadian rhythm appear to be mutually regulated [158]. The cellular oxidative load fluctuates through the day as the basal metabolism changes, which needs to be timely suppressed in certain cells. To this end, NRF2 is transcriptionally regulated by the CLOCK/BMAL1 complex [159,160,161,162], which in turn drives the circadian expression of target antioxidants. Therefore, acute redox and H_2_O_2_ signaling may have to operate on a daily fluctuating background. Given the obligatory role of metabolic H_2_O_2_ signaling in glucose-stimulated insulin secretion [9], it is not surprising that circadian clock-controlled timely suppression of oxidative stress in β-cells is crucial to the function of these cells, disruption of which may lead to diabetes [163,164].

Conversely, oxidative stress can entrain or induce phase changes in the circadian clock in vitro and in vivo [165]. The circadian rhythm may be weakened by increased adaptive antioxidant response as the H_2_O_2_ amplitude will be smaller, thus affecting its action on BMAL [10]. In hepatocytes, NRF2 upregulates the clock repressor gene Cry2 to suppress CLOCK/BMAL1-mediated transcriptional activity, and chemical activation of NRF2 at the peak of endogenous oxidative signals reinforces the circadian amplitude [166]. The pentose phosphatase pathway provides the reducing agent NADPH which in turn regulates the circadian clock [167]. Its perturbation leads to amplitude and phase but not period changes [168]. Taken together, metabolic redox signal and oscillation, NRF2, and the TTFL clock constitute inter-locking loops that coordinately control circadian timekeeping.

## 5. Conclusions

In conclusion, in the present study, we conducted a detailed mathematical analysis of the PTRS system, including SRX-mediated feedback regulation. Our analysis provided novel mechanistic insights into the origins of ultrasensitivity, bistability, and sustained circadian oscillation. The in-depth quantitative understanding can help us to better appreciate the functional significance of intracellular redox signaling and circadian rhythm.

## Data Availability

All model codes are available at the GitHub repository: https://github.com/pulsatility/2024-Mathematical-Modeling-of-PTRS.git (accessed on 27 January 2025).

## Supplementary Materials

The following supporting information can be downloaded at https://www.mdpi.com/article/10.3390/antiox14020235/s1. Figure S1: Sensitivity analyses of ultrasensitivity of the PTRS model; Figure S2: Dynamical responses of the ultrasensitive PTRS model to varying *H*_2_*O*_2_ production rate *k*_0_; Figure S3: Stability analysis using *H*_2_*O*_2_ turnover fluxes and its relationship to the null-curve/bifurcation for bistability of the PTRS model; Figure S4: Additional two-parameter bifurcation and flux analysis for bistability of the PTRS model; Figure S5: Dynamical responses of the bistable PTRS model to varying *H*_2_*O*_2_ production rate *k*_0_; Figure S6: Steady-state and dynamical responses of the P3TRS module to varying *SRX* with parameter setting the same as the Ultrasensitivity Model in Section 1 except *k*_1_ = 20 μM^−1^s^−1^, *k*_2__*a*_ = 22 s^−1^, and *k*_3_ = 0.012 μM^−1^s^−1^ as well as *k*_6_ = 100 s^−1^ and *k*_7_ = 3000 s^−1^; Table S1: Default values of model parameters; Table S2: ODEs of Ultrasensitivity and Bistability Models; Table S3: Algebraic Equations of Ultrasensitivity and Bistability Models; Table S4: ODEs of Oscillation Model; Table S5: Algebraic Equations of Oscillation Model. Additional references [169,170,171,172,173,174,175,176,177,178,179,180,181,182,183,184,185,186,187,188,189,190] are cited in the Supplementary Materials.
