# Supplementary material for: Origins of Ultrasensitivity and Complex Signaling Dynamics of Cellular Hydrogen Peroxide and Peroxiredoxin
Source: Antioxidants (Basel). 2025 Feb 18;14(2):235. doi: 10.3390/antiox14020235 (PMC11852172; doi:10.3390/antiox14020235)
Supplement: Supplementary file 1 [file antioxidants-14-00235-s001.zip › antioxidants-3425267-supplementary.pdf]

Supplemental Materials

Origins of Ultrasensitivity and Complex Signaling Dynamics of Cellular Hydrogen Peroxide and Peroxiredoxin

Shengnan Liu, Jingbo Pi, and Qiang Zhang

Table of Contents

Figures **2**
Figure S1........ 2
Figure S2........ 3
Figure S3........ 4
Figure S4........ 5
Figure S5........ 6
Figure S6........ 7
Tables **8**
Table S1. Default values of model parameters 8
Table S2. ODEs of Ultrasensitivity and Bistability Models........16
Table S3. Algebraic Equations of Ultrasensitivity and Bistability Models........16
Table S4. ODEs of Oscillation Model........16
Table S5. Algebraic Equations of Oscillation Model........17
References **18**

Figures

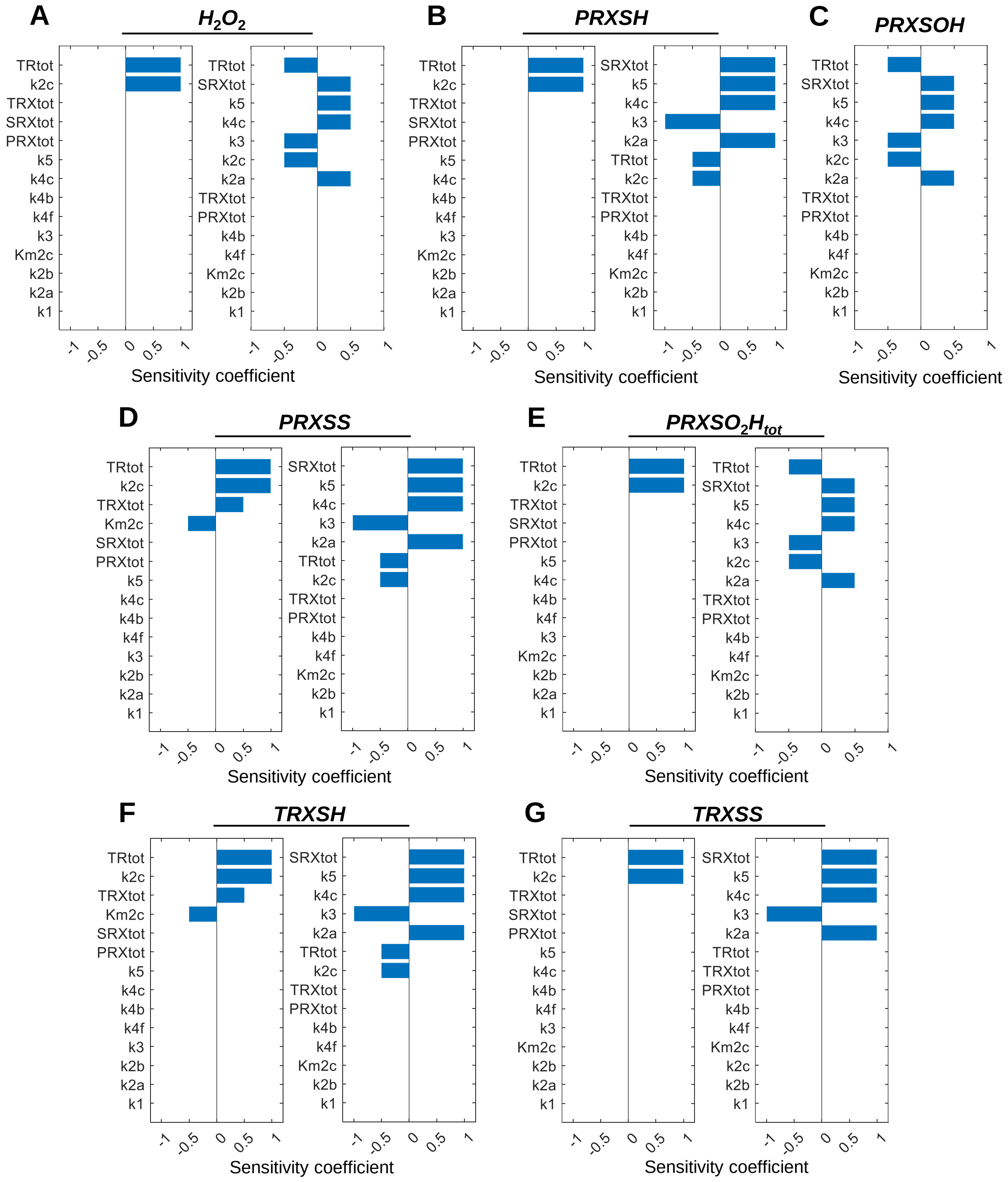

Figure S1. Sensitivity analyses of ultrasensitivity of the PTRS model. Left panels in **(A-B, D-G)**: relative sensitivity coefficients for k_0 associated with LRC_{max} or LRC_{min} occurring at the lower k_0 value near 40 μM/s as in Fig. 2. **(C)** and right panels in **(A-B, D-G)**: relative sensitivity coefficients for k_0 associated with LRC_{max} or LRC_{min} occurring at the higher k_0 value near 139 μM/s

as in Fig. 2. The sensitivity analysis was conducted as follows: after each parameter was increased or decreased by 1% from the default value, the entire response curves as in Fig. 2 was generated by scanning k_0 in the range of 1-1000 μM/s, then the new k_0 values corresponding to the new *LRC* peak or nadir were captured calculate the relative sensitivity coefficients as described in *section 2.3*.

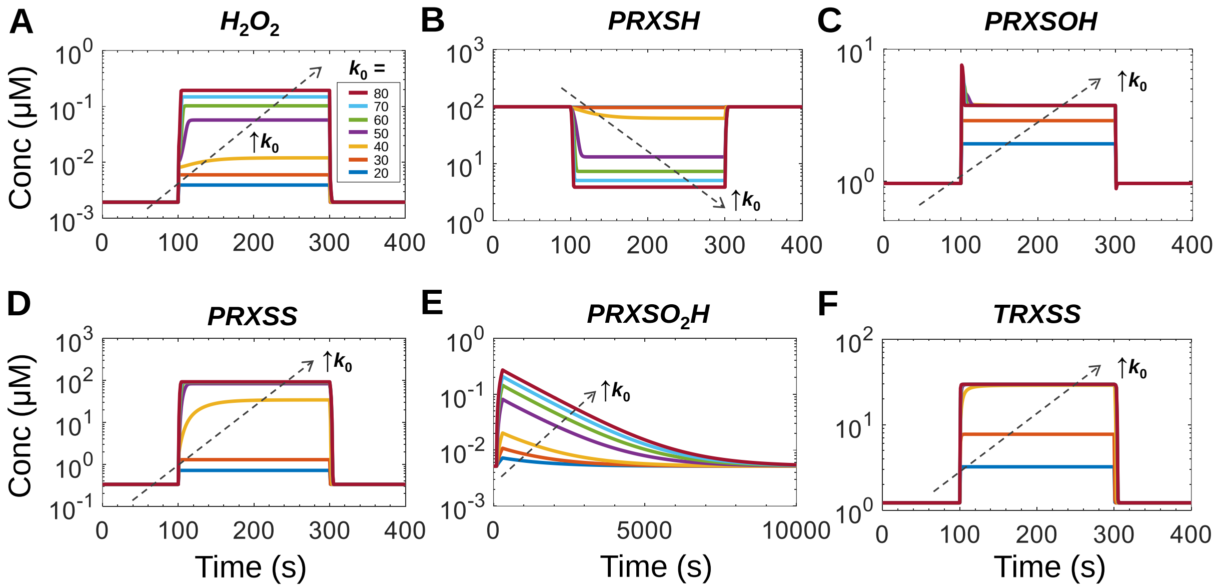

Figure S2. Dynamical responses of the ultrasensitive PTRS model to varying H_2O_2 production rate k_0. (A-F) Variables as indicated are at steady state first from 0-100 s with k_0=10 μM/s. k_0 is then stepped up to different levels as indicated in (A) between 100-300 s, after which it is stepped down to 10 μM/s. Note the time range is much longer for *$PRXSO_2H$* (E).

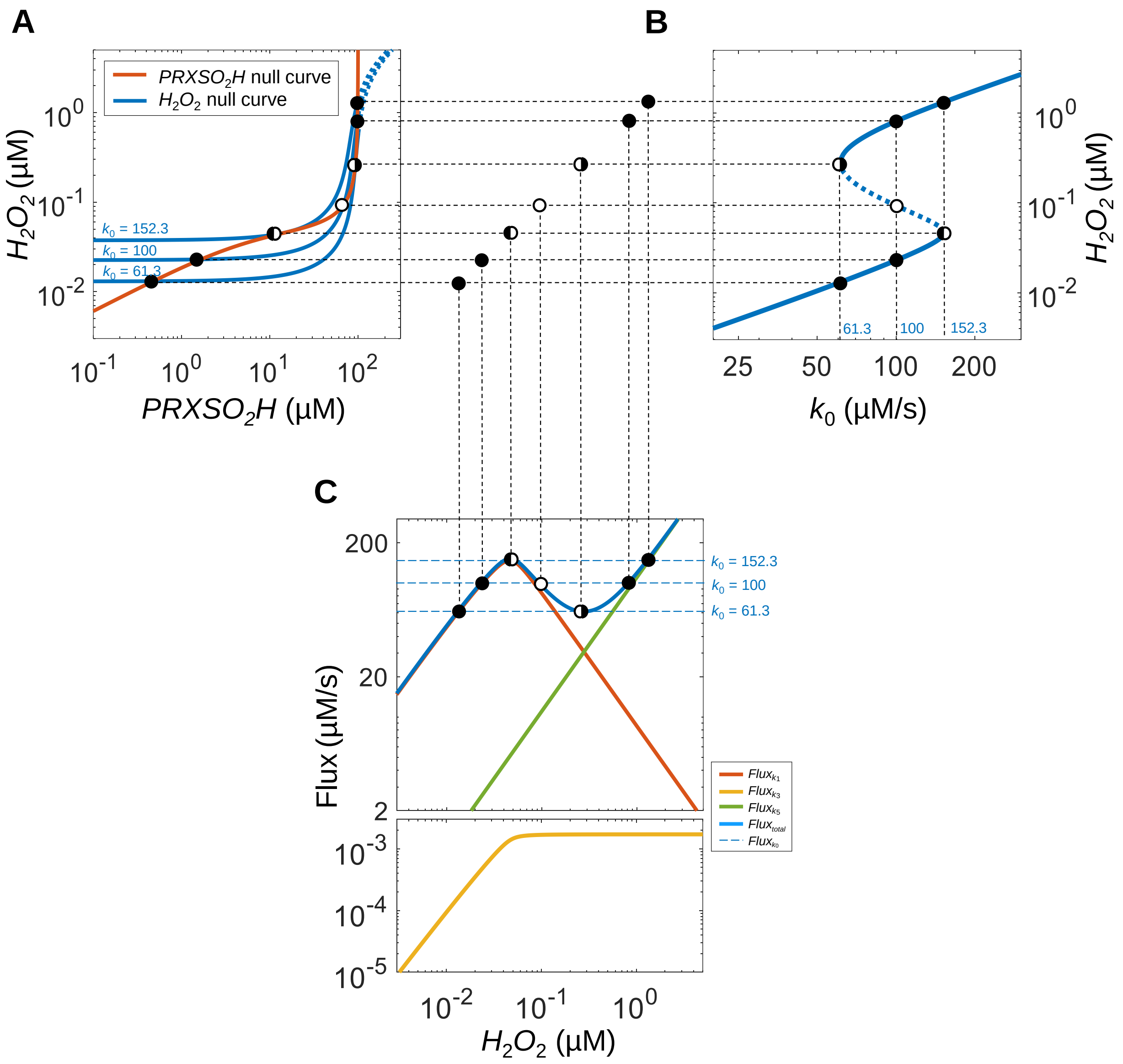

Figure S3. Stability analysis using H_2O_2 turnover fluxes and its relationship to the null-curve/bifurcation for bistability of the PTRS model. (A) Same ultrasensitive H_2O_2 null curve and $PRXSO_2H$ null curves as in Fig. 6A. **(B)** Bifurcation of H_2O_2 with respect to k_0 (zoomed-in view of Fig. 5A). **(C)** $Flux_{k0}$, $Flux_{k1}$, $Flux_{k5}$, and $Flux_{total}$ ($Flux_{total}$ = $Flux_{k1}$ + $Flux_{k5}$ + $Flux_{k3}$) with respect to clamped H_2O_2 concentrations. Intersection points between $Flux_{total}$ and $Flux_{k0}$ represent steady states. Solid dot: stable steady state, empty dot: unstable steady state, half-empty dot: saddle-node unstable steady state. Dashed lines between panels indicate the equivalence of the flux approach and null-curve approach for stability analysis.

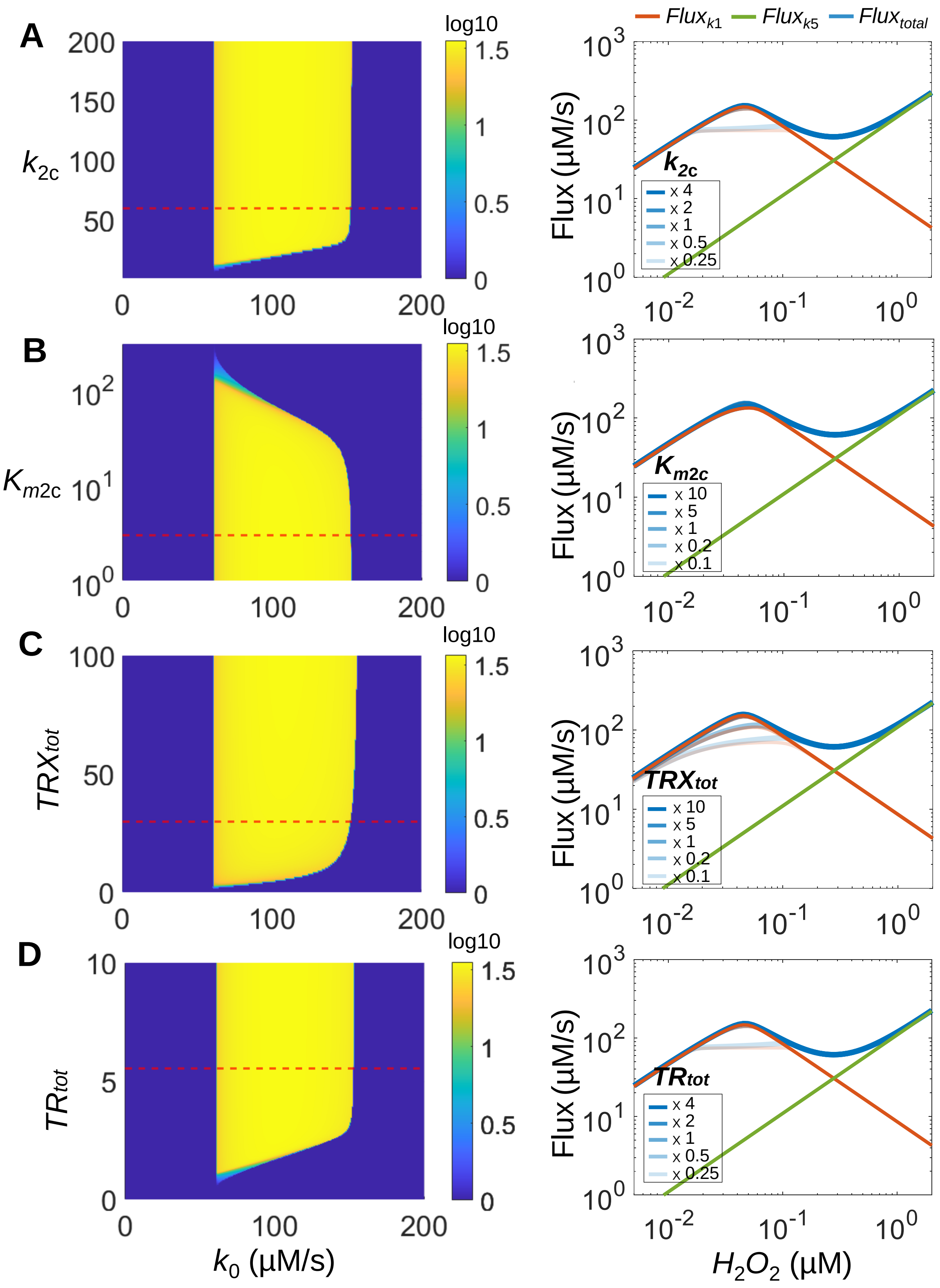

Figure S4. Additional two-parameter bifurcation and flux analysis for bistability of the PTRS model. (A-D) Left panels: Bistable and monostable zones and their boundaries with respect to k_0 and a second parameter as indicated. The heatmap represents the *bistability magnitude*

defined as the log10 ratio of the stable steady-state H_2O_2 levels in the high and low steady states. *Bistability magnitude* > 0: bistable; *bistability magnitude* = 0: monostable (dark blue region). Horizontal red dashed lines: default values of the second parameters. **Right panels:** Flux analysis for the corresponding parameters varied in left panels. The color code for different fluxes is indicated on the top. The fold change of the parameter value relative to the default value (x 1) is indicated with lines of varying shade.

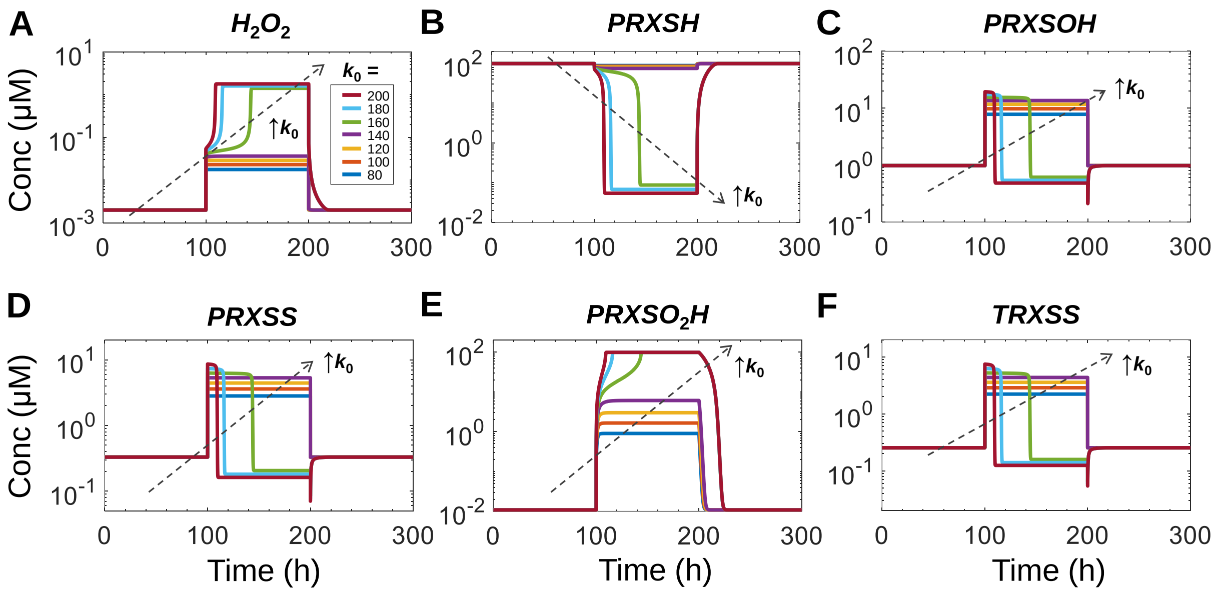

Figure S5. Dynamical responses of the bistable PTRS model to varying H_2O_2 production rate k_0. (A-F) Variables as indicated are at steady state first from 0-100 h with k_0=10 μM/s. k_0 is then stepped up to different levels as indicated in (A) between 100-200 h, after which it is stepped down to 10 μM/s.

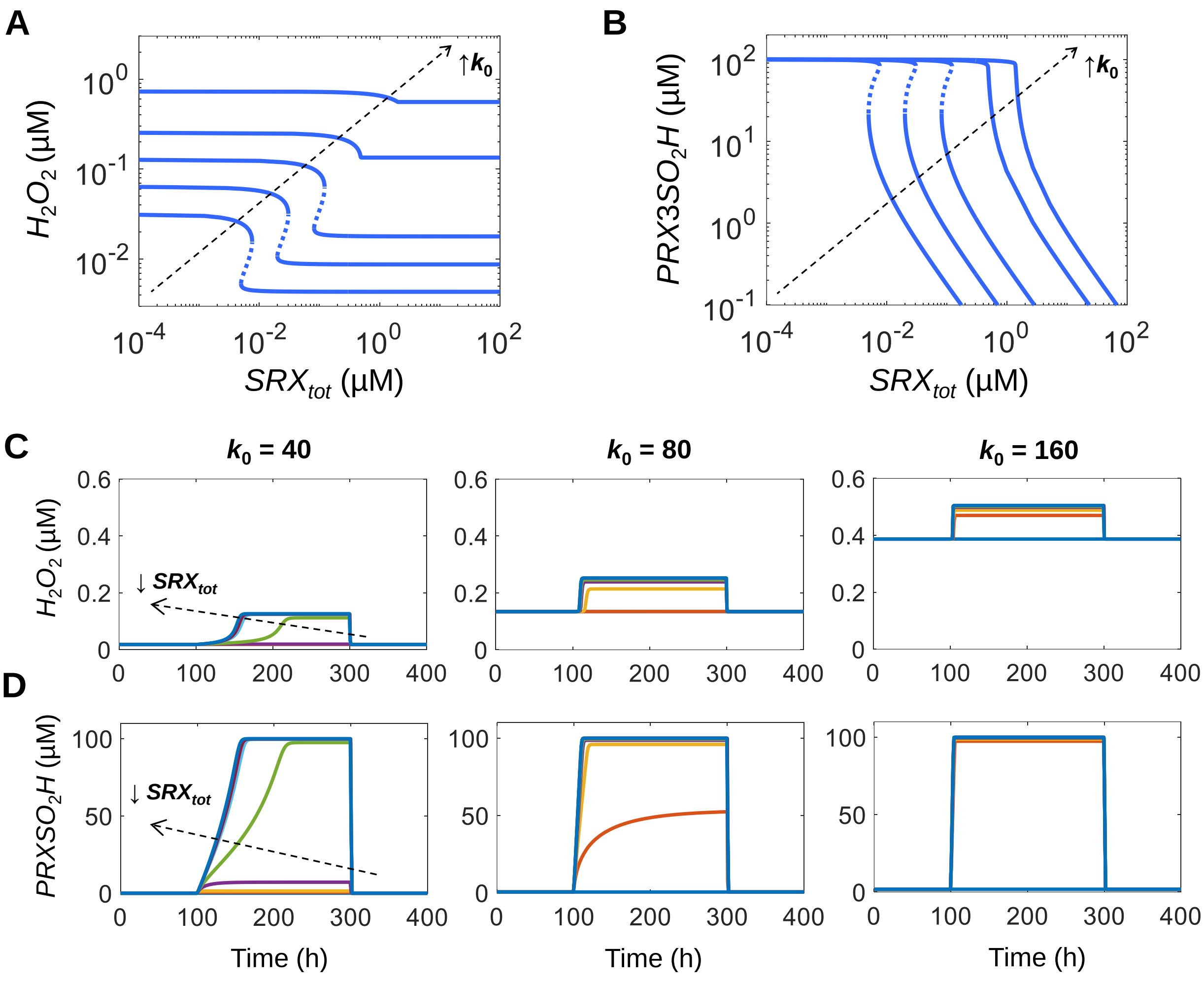

Figure S6. Steady-state and dynamical responses of the P3TRS module to varying *SRX* with parameter setting the same as the Ultrasensitivity Model in Section 1 except k_1=20 μM^{-1}s^{-1}, k_{2a}=22 s^{-1}, and k_3=0.012 μM^{-1}s^{-1} as well as k_6=100 s^{-1} and k_7=3000 s^{-1}. (A-B) Ultrasensitivity or saddle-node bifurcation of steady-state levels of mitochondrial H_2O_2 and $PRX3SO_2H$ respectively with respect to SRX_{tot} under different k_0 conditions (10, 20, 40, 80, 160 μM/s). Solid line: stable steady state, dashed line: unstable steady state. **(C-D)** Time-course responses of mitochondrial H_2O_2 and $PRX3SO_2H$ respectively to varying SRX_{tot} values under different k_0 conditions as indicated. The P3TRS module is at a steady state first from 0-100 h with SRX_{tot}=5 μM. SRX_{tot} is then stepped down to different levels (5, 0.5, 0.25, 0.1, 0.05, 0.01, 0.005, 0 μM) between 100-300 h, after which it is stepped up to 5 μM.

Tables

Table S1. Default values of model parameters

<table>
<tr><th>Parameter</th><th>Ultrasensitivity Model</th><th>Bistability Model</th><th>Oscillation Model</th><th>Unit</th></tr>
<tr><td>k_0</td><td colspan="2">10</td><td>84.2</td><td>μM s^{-1}</td></tr>
<tr><td>k_1</td><td colspan="2">50</td><td>20</td><td>$μM^{-1}s^{-1}$</td></tr>
<tr><td>k_{2a}</td><td colspan="2">10</td><td>22</td><td>s^{-1}</td></tr>
<tr><td>k_{2b}</td><td colspan="3">1</td><td>$μM^{-1}s^{-1}$</td></tr>
<tr><td>k_{2c}</td><td>30</td><td colspan="2">60</td><td>s^{-1}</td></tr>
<tr><td>K_{m2c}</td><td colspan="3">3</td><td>μM</td></tr>
<tr><td>k_3</td><td colspan="2">0.002</td><td>0.012</td><td>$μM^{-1}s^{-1}$</td></tr>
<tr><td>k_{4f}</td><td colspan="3">0.0014</td><td>$μM^{-1}s^{-1}$</td></tr>
<tr><td>k_{4b}</td><td colspan="3">0.001</td><td>s^{-1}</td></tr>
<tr><td>k_{4c}</td><td colspan="3">0.006</td><td>s^{-1}</td></tr>
<tr><td>k_{4d}</td><td colspan="2">na</td><td>2.91e-4</td><td>s^{-1}</td></tr>
<tr><td>k_5</td><td>220</td><td colspan="2">110</td><td>s^{-1}</td></tr>
<tr><td>k_6</td><td rowspan="14">na</td><td rowspan="14">na</td><td>100</td><td>s^{-1}</td></tr>
<tr><td>k_7</td><td>3000</td><td>s^{-1}</td></tr>
<tr><td>k_8</td><td>5.2e-4</td><td>μM s^{-1}</td></tr>
<tr><td>k_9</td><td>5.775e-4</td><td>s^{-1}</td></tr>
<tr><td>k_{10}</td><td>0.0717</td><td>$μM^{-1}s^{-1}$</td></tr>
<tr><td>k_{11}</td><td>1.25e-6</td><td>s^{-1}</td></tr>
<tr><td>k_{12f}</td><td>1.5e-4</td><td>s^{-1}</td></tr>
<tr><td>k_{12b}</td><td>1.25e-6</td><td>s^{-1}</td></tr>
<tr><td>k_{13}</td><td>1.93e-5</td><td>s^{-1}</td></tr>
<tr><td>k_{14}</td><td>1.5e-4</td><td>s^{-1}</td></tr>
<tr><td>k_{15}</td><td>1.93e-5</td><td>s^{-1}</td></tr>
<tr><td>k_{16}</td><td>3.85e-4</td><td>s^{-1}</td></tr>
<tr><td>HSP_{90}</td><td>1</td><td>na</td></tr>
<tr><td>V_{ratio}</td><td>0.15</td><td>na</td></tr>
<tr><td>TRX_{tot}</td><td colspan="3">30</td><td>μM</td></tr>
<tr><td>TR_{tot}</td><td>1.38</td><td colspan="2">5.52</td><td>μM</td></tr>
<tr><td>PRX_{tot}</td><td colspan="3">100</td><td>μM</td></tr>
<tr><td>SRX_{tot}</td><td>0.6</td><td>0.3</td><td>na</td><td>μM</td></tr>
<tr><td>H_2O_2 (state variable)</td><td colspan="3">0.001 ~ 1</td><td>μM</td></tr>
</table>

na: not applicable

Sources and justifications of parameter values

$\boldsymbol{k_0}$ – Zero-order rate constant for H_2O_2 production. The production rate varies in a wide range depending on the cell types and organelles, the substrates, and cellular conditions (Boveris et al. 1972, Boveris and Chance 1973, Oshino et al. 1975, Jones 2008, Drechsel and Patel 2010, Starkov et al. 2014, Treberg et al. 2015, Munro et al. 2016, Treberg et al. 2019). The experimentally measured H_2O_2 production rate was commonly reported as nmol/min/mg protein. Based on a number of studies it was recently argued that basal cellular H_2O_2 production rate is at low μM/s and it is unlikely to be >100 μM/s even under oxidative stress (Griffith et al. 2024). The brown adipocytes, heart, and adrenal gland, where

PRX3 circadian oscillations were observed, are metabolically active tissues and thus are expected to have high H_2O_2 production in mitochondria. The effects of a wide range of k_0 values were explored in the main text of the paper.

k_1 – Second-order rate constant for the sulfenylation of *PRXSH* by *H_2O_2* into *PRXSOH*. The value varies depending on the PRX isoforms and cell types involved (Winterbourn and Hampton 2008).

Experimentally determined values:

PRX1	38	$\mu M^{-1}s^{-1}$	Human	(Carvalho et al. 2017)
	110		Human	(Portillo-Ledesma et al. 2018)
PRX2	13		Human	(Peskin et al. 2007)
	100		Human	(Manta et al. 2009)
	160		Human	(Portillo-Ledesma et al. 2018)
PRX3	20		Human	(Cox et al. 2009)
PRX4	22		Human	(Wang et al. 2012)
PRX5	0.3		Human	(Trujillo et al. 2007)
	0.43		Human	(Portillo-Ledesma et al. 2014)

Values used in modeling studies:

The modeling paper by (Nagy et al. 2011) used 20 $\mu M^{-1}s^{-1}$ by citing (Peskin et al. 2007, Cox et al. 2009). The modeling paper by (Benfeitas 2011) used a range of 10-100 $\mu M^{-1}s^{-1}$ by citing (Peskin et al. 2007, Manta et al. 2009). The modeling paper by (Travasso et al. 2017) used 100 $\mu M^{-1}s^{-1}$ by citing (Manta et al. 2009). The two modeling papers by (Selvaggio et al. 2018, Stein et al. 2020) used 40 and 20 $\mu M^{-1}s^{-1}$ respectively. Recently, citing (Peskin et al. 2007, Manta et al. 2009, Carvalho et al. 2017, Portillo-Ledesma et al. 2018), (Griffith et al. 2024) argued that the rate constant "with recent determinations tending to similar higher values for both these peroxiredoxins" and 100 $\mu M^{-1}s^{-1}$ was used for both PRX1 and 2.

In our study here:

Taken together, 50 $\mu M^{-1}s^{-1}$ was used as the default value for the generic Ultrasensitivity and Bistability Models, with 100, 100, and 20 $\mu M^{-1}s^{-1}$ as isoform-specific values for PRX1, 2, and 3 respectively; and 20 $\mu M^{-1}s^{-1}$ was used for the PRX3 Oscillation Model.

k_{2a} – The first-order rate constant for the resolution of *PRXSOH* into *PRXSS*.

Experimentally determined values:

PRX1	9	s^{-1}	Human	(Carvalho et al. 2017)
	12.9		Human	(Portillo-Ledesma et al. 2018)
	11-12		Human	(Peskin et al. 2007)
PRX2	1.7		Human	(Peskin et al. 2013)
	0.25		Human	(Carvalho et al. 2017)
	0.64		Human	(Portillo-Ledesma et al. 2018)
	0.2-0.3		Human	(Dalla Rizza et al. 2019)
	0.5		Human	(Peskin et al. 2020)
	0.17 or 0.21		Human	(Villar et al. 2023)
PRX3	22		Human	(Peskin et al. 2013)
PRX5	14.7		Human	(Trujillo et al. 2007)
	18.7		Human	(Portillo-Ledesma et al. 2018)
AhpC	104.8		Salmonella typhimurium	(Portillo-Ledesma et al. 2018)

Values used in modeling studies:

Modeling studies including (Tomalin et al. 2016, Travasso et al. 2017, Stein et al. 2020) used similar values by citing (Peskin et al. 2013). In a modeling study on H_2O_2 metabolism in Jurkat cells (Adimora et al. 2010), 15 s^{-1} was used. In the modeling study (Selvaggio et al. 2018), a range of values 5.9-8.7

s^{-1} was used as the weighted-average for PRX1 and 2 for a variety of 13 different human nucleated cell types; the geomean of these values is 7.23 s^{-1}. In the modeling study (Griffith et al. 2024), 11 and 0.5 s^{-1} were used for PRX1 and 2 respectively by citing some of the experimental studies listed above.

In our study here:
10 s^{-1} was used as the default value for the generic Ultrasensitivity and Bistability Models, with 11, 0.5, and 22 s^{-1} as isoform-specific values for PRX1, 2, and 3 respectively; and 22 s^{-1} was used for the PRX3 Oscillation Model.

k_{2b} – Second-order rate constant for the TRX-mediated reduction of *PRXSS* to *PRXSH*.

Experimentally determined values:

PRX1	1.9	$\mu M^{-1}s^{-1}$	Human, reacting with TRX1	(Villar et al. 2023)
	2.2		Human, reacting with TRX2	
PRX2	0.21		Human, reacting with E *coli.* TRX	(Manta et al. 2009)
	0.61		Human, reacting with TRX1	(Villar et al. 2023).
	0.52		Human, reacting with TRX2	
PRX5	2		Human, reacting with TRX2	(Trujillo et al. 2007)

Values used in modeling studies:
Both the modeling studies by (Travasso et al. 2017, Selvaggio et al. 2018) used 0.21 $\mu M^{-1}s^{-1}$ by citing (Manta et al. 2009). The three modeling papers by (Adimora et al. 2010, Komalapriya et al. 2015, Stein et al. 2020) used 2.1, 0.1 and 0.22 $\mu M^{-1}s^{-1}$ respectively. In the recent modeling study (Griffith et al. 2024): 2.2 and 0.61 were used for PRX1 and PRX2 respectively (Villar et al. 2023).

In our study here:
1 $\mu M^{-1}s^{-1}$ was used as the default value for the generic Ultrasensitivity and Bistability Models, with 2.2, 0.61, and 1 $\mu M^{-1}s^{-1}$ as isoform-specific values for PRX1, 2, and 3 respectively; and 1 $\mu M^{-1}s^{-1}$ was used for the PRX3 Oscillation Model.

k_{2c} – First-order catalytic rate constant (k_{cat}) for the reduction of *TRXSS* to *TRXSH* catalyzed by *TR*, which follows a ping-pong mechanism by using NADPH as a co-substrate (Gromer et al. 1998, Zhong et al. 2000). According to (Selvaggio et al. 2018), except for severe oxidative stress conditions, TR can be considered to be saturated by the co-substrate NADPH thus NADPH is not rate-limiting.

Experimentally determined values:
It was reported that the k_{cat} of human TR1 for 3 different TRX substrates ranged between 25.8-46.6 s^{-1}, and the k_{cat} of mouse TR3 for 3 different TRX substrates ranged between 20-37.9 s^{-1} (Turanov et al. 2006). The geomean of these values is 30 s^{-1}.

Values used in modeling studies:
In the modeling paper (Benfeitas et al. 2014), 10 μMs^{-1} was used as the V_{max}. According to (Selvaggio et al. 2018) the k_{cat} is 76.3 s^{-1} as estimated in (Benfeitas et al. 2014), and a range of V_{max} values 47 – 590 μMs^{-1} was used for a variety of 13 different human nucleated cell types. In the recent modeling study (Griffith et al. 2024), 180 $\mu M\ s^{-1}$ was used as the V_{max} (which is = k_{cat}*TR_{tot}); given TR_{tot} concentration is 2.76 μM as estimated below, the estimated k_{cat} = 65 s^{-1} for V_{max}=180 $\mu M\ s^{-1}$.

In our study here:
30, 60, and 60 s^{-1} were used as the default values for the Ultrasensitivity, Bistability, and PRX3 Oscillation Models, respectively.

K_{m2c} – Michaelis constant (K_m) between the substrate *TRXSS* and enzyme *TR* for the reduction of *TRXSS* to *TRXSH*.

Experimentally determined values:

2.7-5.5 μM was reported for rat TRX with rat TR (Chae et al. 1999). The K_m values of mouse TR3 for 3 different TRX substrates were reported to range between 2.56-5.1 μM μM (Turanov et al. 2006). The K_m values of human TR1 for 3 different TRX substrates were reported to range between 1.41-1.83 μM (Turanov et al. 2006). 2.4 μM was also reported for human TRX with human TR (Manta et al. 2009). The measured K_m values for E coli TRX1 and TR were 3 and 4 μM (Dalla Rizza et al. 2019).

Values used in modeling studies:
In the modeling studies (Benfeitas et al. 2014, Selvaggio et al. 2018, Griffith et al. 2024), 1.8 or 1.83 μM was used.

In our study here:
3 μM was used as the default value for all models.

k_3 – Second-order rate constant for the sulfinylation of *PRXSOH* by H_2O_2 into *$PRXSO_2H$*.

Experimentally determined values:

PRX1	0.00177	$\mu M^{-1}s^{-1}$	Human	(Dalla Rizza et al. 2019).
	0.0013		Human	(Yang et al. 2002, Woo et al. 2010, Selvaggio et al. 2018)*
PRX2	0.012		Human	(Peskin et al. 2013)
	0.00197		Human	(Dalla Rizza et al. 2019)
	0.0034		Human	(Peskin et al. 2020)
	0.0042		Human	(Peskin et al. 2021)
	0.001-0.0025		Human	(Poynton et al. 2016)#
PRX3	0.012		Human	(Peskin et al. 2013)
	0.0176		Human	(Poynton et al. 2016)$

Note: *For human PRX1 (Selvaggio et al. 2018) fitted the kinetic data from (Yang et al. 2002, Woo et al. 2010) to obtain 0.0013 $\mu M^{-1}s^{-1}$. #In (Poynton et al. 2016), the estimated k_3/k_{2a} ratio is 4700 M^{-1} for human PRX2; using k_{2a}=0.2-0.5 s^{-1} as above, k_3=0.001-0.0025 $\mu M^{-1}s^{-1}$ for PRX2. $ In (Poynton et al. 2016), the estimated k_3/k_{2a} ratio is 800 M^{-1} for human PRX3; using k_{2a}=22 s^{-1} as above, k_3=0.0176 $\mu M^{-1}s^{-1}$.

Values used in modeling studies:
The modeling paper (Adimora et al. 2010) used 0.072 $\mu M^{-1}s^{-1}$ for Jurkat cells. The two modeling papers (Travasso et al. 2017, Stein et al. 2020) used 0.012 and 0.014 $\mu M^{-1}s^{-1}$ respectively by citing (Peskin et al. 2013). In the modeling study (Selvaggio et al. 2018), a range of values 0.0017-0.0058 $\mu M^{-1}s^{-1}$ were used as the weighted-average for PRX1 and 2 for a variety of 13 different human nucleated cell types; the geomean of these values is 0.0036 $\mu M^{-1}s^{-1}$. In the recent modeling study (Griffith et al. 2024), 0.0015 and 0.0034 were used for PRX1 and PRX2 respectively.

In our study here:
0.002 $\mu M^{-1}s^{-1}$ was used as the default value for the generic Ultrasensitivity and Bistability models, with 0.0015, 0.0034, and 0.012 $\mu M^{-1}s^{-1}$ as isoform-specific values for PRX1, 2, and 3 respectively; and 0.012 $\mu M^{-1}s^{-1}$ was used for the PRX3 Oscillation Model.

k_{4f}, ***k_{4b}***, and ***k_{4c}*** – These 3 parameters collectively describe the reduction of *$PRXSO_2H$* catalyzed by *SRX*, which is a complex GSH- and ATP-dependent reaction that remains to be fully characterized (Roussel et al. 2008, Roussel et al. 2011, Boukhenouna et al. 2015, Griffith et al. 2024). k_{4f} is the second-order rate constant for the association between *$PRXSO_2H$* and *SRX* to form *$PRXSO_2H$:SRX*, k_{4b} is the first-order rate constant for the dissociation of *$PRXSO_2H$:SRX* back to *$PRXSO_2H$* and *SRX*, and k_{4c} is the catalytic rate constant for the production of *PRXSOH* and recycling of *SRX*.

Experimentally determined values:
This is a slow reaction, for which k_{cat} has been reported to be 0.00167-0.003 s^{-1} for human, rat, and mouse SRX using sulfinylated human PRX1 as substrate or in a similar range with human PRX2 as

substrate (Chang et al. 2004). 0.0033-0.0083 s^{-1} were reported as k_{cat} values for human and rat SRX using sulfinylated human PRX1 as substrate (Jeong et al. 2006) (note: in this study Pi release was measured as a surrogate for the reaction rate of $PRXSO_2H$). 0.025-0.032 s^{-1} were reported as k_{cat} values for yeast SRX and TSA1 (Roussel et al. 2008).

The measured dissociation constant K_d for the binding between human PRX4 and SRX is 4.0–7.0 μM (Wei et al. 2011). Using SRX in the mouse liver mitochondrial and cytosolic fractions and sulfinylated PRX3 as substrate, the measured K_d=0.71 μM, with the association rate constant k_{on}=0.0014 $\mu M^{-1}s^{-1}$ and dissociation rate constant k_{off} = 0.001 s^{-1} (Kil et al. 2015). In (Selvaggio et al. 2018), a Michaelis constant K_m value of 20 μM was estimated for yeast SRX for TSA1 based on (Roussel et al. 2011).

Values used in modeling studies:
This reaction was modeled as a pseudo-first-order reaction in nearly all modeling studies so far. A rate constant of 0.0001 or 0.003 s^{-1} were used in (Adimora et al. 2010, Benfeitas et al. 2014, Travasso et al. 2017), based on some of the experimental papers cited above. In (Selvaggio et al. 2018), a range of pseudo-first-order rate constants of 0.00029-0.0066 s^{-1} were used for a variety of 13 different human nucleated cell types. In the recent modeling study (Griffith et al. 2024), a first-order rate constant of 0.001 s^{-1} was used for both PRX1 and PRX2. In the two modeling studies by (del Olmo et al. 2019, Stein et al. 2020), second-order rate constants of 2E-6 and 0.001 $\mu M^{-1}s^{-1}$ were used.

In our study here:
The Michaelis constant K_m values for PRX1 and 2 are not available. Using the above mammalian k_{cat} range of 0.00167-0.0083 s^{-1}, k_{on}=0.0014 $\mu M^{-1}s^{-1}$ and k_{off} = 0.001 s^{-1}, the corresponding K_m can be 1.9-2.86 μM for composite PRX. Together with the K_d values of 0.71 or 4.0-7.0 μM reported for PRX3 and 4 above, these parameter conditions collectively suggest that SRX may be readily saturated by $PRXSO_2H$ given that total PRX abundance is often 100 μM or higher and SRX is at low μM (see details below). Therefore, a pseudo-first-order approximation may not apply, and we chose to model this SRX-mediated reaction more explicitly. For all models in our study here, k_{4f} and k_{4b} used the above k_{on} and k_{off} values respectively, and k_{4c} used 0.006 s^{-1} as the default values, which produce a K_m=5 μM. For very low levels of $PRXSO_2H$, these values produce a pseudo first-order rate constant of 0.00036 and 0.00072 s^{-1} for a total SRX concentration of 0.3 and 0.6 μM respectively.

$\boldsymbol{k_{4d}}$ – First-order rate constant for the degradation of *SRX* in *$PRXSO_2H$:SRX* complex. In mouse tissues including heart, brown adipose, adrenal gland, and liver, it was shown that knockout of PRX3 resulted in dramatic reduction of mitochondrial SRX abundance (Kil et al. 2015). This result suggested that while free mitochondrial SRX is degraded fast, it is protected by binding to PRX3 in the mitochondrion. We therefore set k_{4d} to be lower than k_{16} which is the degradation rate constant for free mitochondrial SRX.

$\boldsymbol{k_5}$ – First-order rate constant for non-PRX-mediated H_2O_2 elimination. This value is expected to vary widely depending on the location in the cells and cell types. In the modeling study (Selvaggio et al. 2018), a range of values 44-210 s^{-1} were used for a variety of 13 different human nucleated cell types. The geomean of these values is 110 s^{-1}. 100 s^{-1} was used in the recent modeling study (Griffith et al. 2024). In our study here, 220, 110, and 110 s^{-1} were used as the default values for the Ultrasensitivity, Bistability, and PRX3 Oscillation Models, respectively.

$\boldsymbol{k_6}$ – First-order rate constant for H_2O_2 exiting and entering the mitochondrion through diffusion/permeation. Cross-membrane translocation of H_2O_2 is expected to be fast through facilitated bidirectional diffusion, thus not causing or providing time delay in the negative feedback loop. The values are the same for both directions of diffusion and a range of 0.2-1 s^{-1} were used in modeling studies (Gauthier et al. 2013, Kembro et al. 2013, del Olmo et al. 2019). 20 s^{-1} was used for cross-membrane pseudo-first order rate constant in the modeling study (Orrico et al. 2022) by citing (Antunes and Cadenas 2000). A higher value of 100 s^{-1} was used in our study here to make sure that cytosolic H_2O_2 can reach sufficient levels that can oxidize *SRX*. If mitochondrion-released H_2O_2 can be limited to the vicinity instead of distributed into the whole cytosol, a much lower k_6 value may be used.

k_7 – First-order rate constant for H_2O_{2cyto} elimination. It represents the rate constant for the collective elimination of cytosolic H_2O_2 by all relevant enzymes including PRX1 and 2. While it varies depending on cell types, the default value of 3000 s^{-1} here is a reasonable estimate based on the cellular abundance of PRX1 and 2 and k_1 values.

k_8 – Zero-order rate constant for *de novo* synthesis of SRX_{cyto}. The value was adjusted so that there is sufficient supply of *SRX* to the mitochondrion.

k_9 – First-order rate constant for SRX_{cyto} degradation. The half-life of SRX in RAW264.7 macrophage cells was estimated to be 5.3 h (Kim et al. 2010). In HeLa cells transfected with SRX, non-mitochondrion-targeted SRX half-life is 4 h, while mitochondrion-targeted SRX half-life is 0.5 h (Kil et al. 2015). In our model here, 5.775e-4 s^{-1} was used for the Oscillation Model, corresponding to a half-life of 1/3 h. This fast turnover is necessary to ensure that total SRX_{cyto} concentration is within a reasonable range while providing sufficient reserve capacity for SRX mitochondrial translocation.

k_{10} – Second-order rate constant for oxidation of SRX_{cyto} by H_2O_{2cyto} into *SRXSOH*. The value was set to be low enough that the oxidation is a slow process according to (Kil et al. 2015).

k_{11} – First-order rate constant for reduction of *SRXSOH* to SRX_{cyto}. We assumed that the oxidation of SRX by H_2O_2 is much more favored than the reverse reduction, therefore k_{11} is set to a low value here.

k_{12f} – First-order rate constant for the formation of *SRXSSHSP90* complex between HSP_{90} and *SRXSOH*, where HSP_{90} is assumed to be at constant unity. The value was set to be low to account for the slow reaction according to (Kil et al. 2015), which indicated that "formation of the disulfide-linked SRX-HSP90 complex is a slow process that requires H_2O_2 induced disulfide formation between Cys99 of SRX and the M domain of HSP90 and that this association is essential for mitochondrial import of SRX."

k_{12b} – First-order rate constant for the dissociation of *SRXSSHSP90* complex to *SRXSOH* and HSP_{90}. We assumed that the association of *SRXSOH* and HSP_{90} is much more favored than the dissociation, therefore k_{12b} is set to a low value here.

k_{13} – First-order rate constant for *SRXSOH* degradation in the cytosol. The value was adjusted to correspond to a half-life of 10 h and is the same as k_{15}.

k_{14} – First-order rate constant for mitochondrial translocation of *SRXSSHSP90*. In response to oxidizing signals, the mitochondrial translocation of SRX is a relatively slow process. In both HeLa and A549 cells treated with H_2O_2 for 10 minutes, it was observed that a fraction of cytosolic SRX translocated into mitochondria at 4 and 8 hours (no observations at earlier time points were reported) (Noh et al. 2009). Similarly, in Y-1 (adrenocortical) cells treated with H_2O_2 for 10 minutes, SRX appeared in mitochondria at 2 h and peaked at 4 h with some still remaining at 8 h (Kil et al. 2015). In A549 cells treated with H_2O_2 for 10 minutes, SRX did not appear in mitochondria until at 4 h (no appearance at 2 h) and peaked at 8 h (Kil et al. 2015). In adrenal glands treated with ACTH or rotenone *ex vivo*, both of which caused mitochondrial production of H_2O_2 that leaked to the cytosol, SRX in mitochondria rose slowly to 6 h (Kil et al. 2015). Taken together, these experiments indicate that it may take hours for cytosolic SRX from being oxidized to translocating into mitochondrion. Together with k_{12f}, k_{14} was set corresponding to an average of 1.85 h for mitochondrial translocation.

k_{15} – First-order rate constant for *SRXSSHSP90* degradation. The HSP_{90} in the complex is not tracked since cytosolic HSP_{90} is set constant. The value was set the same as k_{13}.

k_{16} – First-order rate constant for SRX_{mito} degradation, which corresponds to a half-life of 0.5 h, as observed in HeLa cells transfected with mitochondrion-targeted SRX (Kil et al. 2015).

HSP_{90} – Cytosolic HSP_{90} is assumed to be in excess, so its binding with *SRXSOH* does not cause its

consumption. It was set to a constant value of 1.

V_{ratio} – Mitochondrial matrix:cytosolic volume ratio. The cellular mitochondrial density is dynamic, varying between cell types and physiological states with theoretical maximum packing density of 0.36-0.41 (Vazquez 2018). In cardiomyocytes, the mitochondrial volume density ranges between 0.22-0.37 (Barth et al. 1992). In adrenal gland such as the zona fasciculata which secretes glucocorticoid, the mitochondrial volume percentage is 0.33 in rats (Markowska et al. 1994). Brown adipocytes also have high density of mitochondria which give cells the brown color. Mitochondrial density in many cell types including cardiomyocytes can also be found in (Vazquez 2018). The fraction of the matrix of the overall mitochondrial volume in mammalian cells was estimated around 60% (Gerencser et al. 2012). Taken together, we set V_{ratio} to 0.15 as the default value here.

TRX_{tot} – Total TRX concentration. The reported value in the literature varies depending on the species, cell types, and isoforms. Komalapriya et al. estimated that TRX1 in yeast is at 1.1518 μM (Komalapriya et al. 2015), based on a proteomic study by (Ghaemmaghami et al. 2003). A similar value, 0.7 μM, was estimated for TRX1 by a modeling study (Tomalin et al. 2016), based on another proteomic study in yeast (Marguerat et al. 2012). Based on a study measuring immunoreactive TRX in the bovine (1-week calf) liver, thymus, spleen, lung, tongue, brain, heart, erythrocytes, platelets, and plasma (Holmgren and Luthman 1978), it was estimated that the concentrations can range between 2-12 μM (Chang et al. 2004). In Jurkat cells, TRX1 was estimated to be at 0.505 μM (Adimora et al. 2010). In the modeling paper by (Travasso et al. 2017), 10 μM was used based on the Protein Abundance Database (PAXdb) (Wang et al. 2015). In the modeling study by (Stein et al. 2020), 7.7 μM was used for TRX2 in HeLa cells. In the modeling study by (Selvaggio et al. 2018), which believed that TRX concentrations had been underestimated in previous studies, a cytosolic TRX1 concentration range of 17-69 μM was estimated for a variety of 13 different human nucleated cell types based on quantitative proteomic studies (Geiger et al. 2012, Liebermeister et al. 2014, Wiśniewski et al. 2016) by using the mass fraction method (Milo 2013). The geomean of these values is 31 μM. These values are consistent with the range determined with other methods where the maximal TRX concentration is 110 μM in U937 cells (Montano et al. 2014). In the recent modeling study (Griffith et al. 2024), 20 μM was used. TRX also participates in the reduction of other Protein-SS, but these reactions can be safely omitted in the model according to (Selvaggio et al. 2018). In our model here, 30 μM was used as the default value for all three models.

TR_{tot} – Total TRX reductase (TR) concentration. In the modeling study by (Selvaggio et al. 2018), a cytosolic concentration range of 0.62-7.7 μM were estimated for a variety of 13 different human nucleated cell types based on quantitative proteomic studies (Geiger et al. 2012, Liebermeister et al. 2014, Wiśniewski et al. 2016) by using the mass fraction method (Milo 2013). The geomean of these values is 2.76 μM. In out model here, 1.38, 5.52, and 5.52 μM were used for the Ultrasensitivity, Bistability, and PRX3 Oscillation Models, respectively.

PRX_{tot} – Total concentration of PRX. The value varies depending on the PRX isoforms and subcellular locations. In Jurkat cells, PRX1 and PRX2 combined was reported to be at 65 μM without considering compartmentalization and PRX3 is at 125 μM (Cox et al. 2009). PRX2 in RBC was estimated to be 240 μM (Moore et al. 1991). In the study by (Selvaggio et al. 2018), a cytosolic concentration range of 47-120 μM was estimated for total PRX1 and 2-46 μM for PRX2 for a variety of 13 different human nucleated cell types based on quantitative proteomic studies (Geiger et al. 2012, Wiśniewski et al. 2016) by using the mass fraction method (Milo 2013). The geomean of these values is 91 μM. In the recent modeling study (Griffith et al. 2024), 110 and 30 μM were used for PRX1 and 2 respectively. In our model here, 100 μM was used as the default value for all three models. 30, 100, 240 μM were also explored for PRX2-specific Ultrasensitivity and Bistability Models.

SRX_{tot} – Total concentration of SRX. Cytosolic SRX can be at much higher abundance than mitochondrial SRX (Noh et al. 2009). In the modeling study by (Selvaggio et al. 2018), a cytosolic concentration range of 0.065-1.5 μM were estimated for a variety of 13 different human nucleated cell types based on quantitative proteomic studies (Geiger et al. 2012, Liebermeister et al. 2014, Wiśniewski et al. 2016) by using the mass fraction method (Milo 2013). The geomean of these values is 0.3 μM. In the

modeling study by (Stein et al. 2020), 8.8E-3 μM was used as the initial value for HeLa cells but it is unclear what steady-state levels it reaches given a constant influx of SRX into the mitochondrion implemented in the model. In our study here, 0.6 and 0.3 μM were used as the default values for the Ultrasensitivity and Bistability Models, respectively; the concentration of *SRX* is not fixed in the PRX3 Oscillation Model.

H_2O_2 – Cytosolic or mitochondrial *H_2O_2* concentrations. At basal conditions, the concentrations are in low nM range but vary depending on cell types and subcellular locations. In the review article by (Stone and Yang 2006), it was summarized that in mammalian cells, the physiological intracellular H_2O_2 concentration can range between 0.001 – 0.7 μM, with the high values occurring under stimulated conditions. Higher concentrations can lead to cell death. In Jurkat cells, it was demonstrated that cytosolic H_2O_2 was < 0.7 μM at resting state, cells underwent apoptosis at 1~3 μM, and underwent necrosis at > 3 μM (Antunes and Cadenas 2001). In our model here, the *H_2O_2* concentration ranges from nM to μM depending on the production rate and other parameter conditions.

Table S2. ODEs of Ultrasensitivity and Bistability Models

$\frac{dH_2O_2}{dt}$	=	$k_0 - k_1 \cdot H_2O_2 \cdot PRXSH - k_3 \cdot H_2O_2 \cdot PRXSOH - k_5 \cdot H_2O_2$
$\frac{dPRXSH}{dt}$	=	$- k_1 \cdot H_2O_2 \cdot PRXSH + k_{2b} \cdot TRXSH \cdot PRXSS$
$\frac{dPRXSOH}{dt}$	=	$k_1 \cdot H_2O_2 \cdot PRXSH - k_{2a} \cdot PRXSOH - k_3 \cdot H_2O_2 \cdot PRXSOH$ $+ k_{4c} \cdot PRXSO_2H{:}SRX$
$\frac{dPRXSS}{dt}$	=	$k_{2a} \cdot PRXSOH - k_{2b} \cdot TRXSH \cdot PRXSS$
$\frac{dPRXSO_2H}{dt}$	=	$k_3 \cdot H_2O_2 \cdot PRXSOH - k_{4f} \cdot SRX \cdot PRXSO_2H + k_{4b} \cdot PRXSO_2H{:}SRX$
$\frac{dTRXSS}{dt}$	=	$k_{2b} \cdot TRXSH \cdot PRXSS - k_{2c} \cdot 0.5 \cdot (TR_{tot} + TRXSS + K_{m2c}$ $- ((TR_{tot} + TRXSS + K_{m2c})^2 - 4 \cdot TR_{tot} \cdot TRXSS)^{0.5})$

Table S3. Algebraic Equations of Ultrasensitivity and Bistability Models

$PRXSO_2H{:}SRX$	=	$PRX_{tot} - PRX - PRXSOH - PRXSS - PRXSO_2H$
$TRXSH$	=	$TRX_{tot} - TRXSS$
SRX	=	$SRX_{tot} - PRXSO_2H{:}SRX$

Table S4. ODEs of Oscillation Model

$\frac{dH_2O_{2mito}}{dt}$	=	$k_0 - k_1 \cdot H_2O_{2mito} \cdot PRX3SH - k_3 \cdot H_2O_{2mito} \cdot PRX3SOH - k_5 \cdot H_2O_{2mito}$ $- k_6 \cdot H_2O_{2mito} + k_6 / V_{ratio} \cdot H_2O_{2cyto}$
$\frac{dH_2O_{2cyto}}{dt}$	=	$k_6 \cdot V_{ratio} \cdot H_2O_{2mito} - k_7 \cdot H_2O_{2cyto} - k_6 \cdot H_2O_{2cyto} - k_{10} \cdot SRX_{cyto} \cdot H_2O_{2cyto}$
$\frac{dSRX_{cyto}}{dt}$	=	$k_8 - k_9 \cdot SRX_{cyto} - k_{10} \cdot SRX_{cyto} \cdot H_2O_{2cyto} + k_{11} \cdot SRXSOH_{cyto}$
$\frac{dSRXSOH_{cyto}}{dt}$	=	$k_{10} \cdot SRX_{cyto} \cdot H_2O_{2cyto} - k_{11} \cdot SRXSOH_{cyto} - k_{12f} \cdot SRXSOH_{cyto} \cdot HSP90$ $+ k_{12b} \cdot SRXSSHSP90 - k_{13} \cdot SRXSOH_{cyto}$
$\frac{dSRXSSHSP90}{dt}$	=	$k_{12f} \cdot SRXSOH \cdot HSP90 - k_{12b} \cdot SRXSSHSP90 - k_{14} \cdot SRXSSHSP90-$ $k_{15} \cdot SRXSSHSP90$
$\frac{dSRX_{mito}}{dt}$	=	$k_{14} / V_{ratio} \cdot SRXSSHSP90 - k_{16} \cdot SRX_{mito} - k_{4f} \cdot SRX_{mito} \cdot PRX3SO_2H +$ $k_{4b} \cdot PRX3SO_2H{:}SRX + k_{4c} \cdot PRX3SO_2H{:}SRX$
$\frac{dPRX3SO_2H{:}SRX}{dt}$	=	$k_{4f} \cdot SRX_{mito} \cdot PRX3SO_2H - k_{4b} \cdot PRX3SO_2H{:}SRX - k_{4c} \cdot PRX3SO_2H{:}SRX -$ $k_{4d} \cdot PRX3SO_2H{:}SRX$
$\frac{dPRX3SH}{dt}$	=	$- k_1 \cdot H_2O_{2mito} \cdot PRX3SH + k_{2b} \cdot TRXSH \cdot PRX3SS$

$\frac{dPRX3SOH}{dt}$	=	$k_1 \cdot H_2O_{2mito} \cdot PRX3SH - k_{2a} \cdot PRX3SOH - k_3 \cdot H_2O_{2mito} \cdot PRX3SOH + k_{4c} \cdot PRX3SO_2H{:}SRX$
$\frac{dPRX3SO_2H}{dt}$	=	$k_3 \cdot H_2O_{2mito} \cdot PRX3SOH - k_{4f} \cdot SRX_{mito} \cdot PRX3SO_2H + k_{4b} \cdot PRX3SO_2H{:}SRX + k_{4d} \cdot PRX3SO_2H{:}SRX$
$\frac{dTRXSS}{dt}$	=	$k_{2b} \cdot TRXSH \cdot PRX3SS - k_{2c} \cdot 0.5 \cdot (TR_{tot} + TRXSS + K_{m2c} - ((TR_{tot} + TRXSS + K_{m2c})^2 - 4 \cdot TR_{tot} \cdot TRXSS)^{0.5})$

Table S5. Algebraic Equations of Oscillation Model

$PRX3SS$	=	$PRX3_{tot} - PRX3SH - PRX3SOH - PRX3SO_2H - PRX3SO_2H{:}SRX$
$PRX3SO_2H_{tot}$	=	$PRX3SO_2H + PRX3SO_2H{:}SRX$
SRX_{mito_tot}	=	$SRX_{mito} + PRX3SO_2H{:}SRX$
SRX_{cyto_tot}	=	$SRX_{cyto} + SRXSOH + SRXSSHSP90$

References

Adimora, N. J., D. P. Jones and M. L. Kemp (2010). "A model of redox kinetics implicates the thiol proteome in cellular hydrogen peroxide responses." Antioxidants & redox signaling **13**(6): 731-743.

Antunes, F. and E. Cadenas (2000). "Estimation of H2O2 gradients across biomembranes." FEBS Lett **475**(2): 121-126.

Antunes, F. and E. Cadenas (2001). "Cellular titration of apoptosis with steady state concentrations of H 2 O 2: submicromolar levels of H 2 O 2 induce apoptosis through Fenton chemistry independent of the cellular thiol state." Free Radical Biology and Medicine **30**(9): 1008-1018.

Barth, E., G. Stämmler, B. Speiser and J. Schaper (1992). "Ultrastructural quantitation of mitochondria and myofilaments in cardiac muscle from 10 different animal species including man." Journal of Molecular and Cellular Cardiology **24**(7): 669-681.

Benfeitas, R., G. Selvaggio, F. Antunes, P. M. Coelho and A. Salvador (2014). "Hydrogen peroxide metabolism and sensing in human erythrocytes: a validated kinetic model and reappraisal of the role of peroxiredoxin II." Free Radical Biology and Medicine **74**: 35-49.

Benfeitas, R. M. V. (2011). The physiological role of peroxiredoxin 2 in human erythrocytes: a kinetic analysis.

Boukhenouna, S., H. Mazon, G. Branlant, C. Jacob, M. B. Toledano and S. Rahuel-Clermont (2015). "Evidence That Glutathione and the Glutathione System Efficiently Recycle 1-Cys Sulfiredoxin In Vivo." Antioxidants & Redox Signaling **22**(9): 731-743.

Boveris, A. and B. Chance (1973). "The mitochondrial generation of hydrogen peroxide. General properties and effect of hyperbaric oxygen." Biochemical Journal **134**(3): 707-716.

Boveris, A., N. Oshino and B. Chance (1972). "The cellular production of hydrogen peroxide." Biochemical Journal **128**(3): 617-630.

Carvalho, L. A. C., D. R. Truzzi, T. S. Fallani, S. V. Alves, J. C. Toledo, Jr., O. Augusto, L. E. S. Netto and F. C. Meotti (2017). "Urate hydroperoxide oxidizes human peroxiredoxin 1 and peroxiredoxin 2." J Biol Chem **292**(21): 8705-8715.

Chae, H. Z., H. J. Kim, S. W. Kang and S. G. Rhee (1999). "Characterization of three isoforms of mammalian peroxiredoxin that reduce peroxides in the presence of thioredoxin." Diabetes Res Clin Pract **45**(2-3): 101-112.

Chang, T.-S., W. Jeong, H. A. Woo, S. M. Lee, S. Park and S. G. Rhee (2004). "Characterization of mammalian sulfiredoxin and its reactivation of hyperoxidized peroxiredoxin through reduction of cysteine sulfinic acid in the active site to cysteine." Journal of Biological Chemistry **279**(49): 50994-51001.

Cox, A. G., A. G. Pearson, J. M. Pullar, T. J. Jönsson, W. T. Lowther, C. C. Winterbourn and M. B. Hampton (2009). "Mitochondrial peroxiredoxin 3 is more resilient to hyperoxidation than cytoplasmic peroxiredoxins." Biochemical Journal **421**(1): 51-58.

Cox, A. G., A. V. Peskin, L. N. Paton, C. C. Winterbourn and M. B. Hampton (2009). "Redox potential and peroxide reactivity of human peroxiredoxin 3." Biochemistry **48**(27): 6495-6501.

Dalla Rizza, J., L. M. Randall, J. Santos, G. Ferrer-Sueta and A. Denicola (2019). "Differential parameters between cytosolic 2-Cys peroxiredoxins, PRDX1 and PRDX2." Protein Science **28**(1): 191-201.

del Olmo, M., A. Kramer and H. Herzel (2019). "A Robust Model for Circadian Redox Oscillations." Int J Mol Sci **20**(9).

Drechsel, D. A. and M. Patel (2010). "Respiration-dependent H2O2 removal in brain mitochondria via the thioredoxin/peroxiredoxin system." J Biol Chem **285**(36): 27850-27858.

Gauthier, L. D., J. L. Greenstein, B. O'Rourke and R. L. Winslow (2013). "An integrated mitochondrial ROS production and scavenging model: implications for heart failure." Biophys J **105**(12): 2832-2842.

Geiger, T., A. Wehner, C. Schaab, J. Cox and M. Mann (2012). "Comparative proteomic analysis of eleven common cell lines reveals ubiquitous but varying expression of most proteins." Mol Cell Proteomics **11**(3): M111.014050.

Ghaemmaghami, S., W.-K. Huh, K. Bower, R. W. Howson, A. Belle, N. Dephoure, E. K. O'Shea and J. S. Weissman (2003). "Global analysis of protein expression in yeast." Nature **425**(6959): 737-741.

Griffith, M., A. Araújo, R. Travasso and A. Salvador (2024). "The architecture of redox microdomains: Cascading gradients and peroxiredoxins' redox-oligomeric coupling integrate redox signaling and antioxidant protection." Redox Biology **69**: 103000.

Gromer, S., L. D. Arscott, C. H. Williams, Jr., R. H. Schirmer and K. Becker (1998). "Human placenta thioredoxin reductase. Isolation of the selenoenzyme, steady state kinetics, and inhibition by therapeutic gold compounds." J Biol Chem **273**(32): 20096-20101.

Holmgren, A. and M. Luthman (1978). "Tissue distribution and subcellular localization of bovine thioredoxin determined by radioimmunoassay." Biochemistry **17**(19): 4071-4077.

Jeong, W., S. J. Park, T. S. Chang, D. Y. Lee and S. G. Rhee (2006). "Molecular mechanism of the reduction of cysteine sulfinic acid of peroxiredoxin to cysteine by mammalian sulfiredoxin." J Biol Chem **281**(20): 14400-14407.

Jones, D. P. (2008). "Radical-free biology of oxidative stress." American Journal of Physiology-Cell Physiology **295**(4): C849-C868.

Kembro, J. M., M. A. Aon, R. L. Winslow, B. O'Rourke and S. Cortassa (2013). "Integrating mitochondrial energetics, redox and ROS metabolic networks: a two-compartment model." Biophys J **104**(2): 332-343.

Kil, I. S., K. W. Ryu, S. K. Lee, J. Y. Kim, S. Y. Chu, J. H. Kim, S. Park and S. G. Rhee (2015). "Circadian oscillation of sulfiredoxin in the mitochondria." Molecular cell **59**(4): 651-663.

Kim, H., Y. Jung, B. S. Shin, H. Kim, H. Song, S. H. Bae, S. G. Rhee and W. Jeong (2010). "Redox regulation of lipopolysaccharide-mediated sulfiredoxin induction, which depends on both AP-1 and Nrf2." J Biol Chem **285**(45): 34419-34428.

Komalapriya, C., D. Kaloriti, A. T. Tillmann, Z. Yin, C. Herrero-de-Dios, M. D. Jacobsen, R. C. Belmonte, G. Cameron, K. Haynes and C. Grebogi (2015). "Integrative model of oxidative stress adaptation in the fungal pathogen Candida albicans." PloS one **10**(9): e0137750.

Liebermeister, W., E. Noor, A. Flamholz, D. Davidi, J. Bernhardt and R. Milo (2014). "Visual account of protein investment in cellular functions." Proc Natl Acad Sci U S A **111**(23): 8488-8493.

Manta, B., M. Hugo, C. Ortiz, G. Ferrer-Sueta, M. Trujillo and A. Denicola (2009). "The peroxidase and peroxynitrite reductase activity of human erythrocyte peroxiredoxin 2." Archives of biochemistry and biophysics **484**(2): 146-154.

Marguerat, S., A. Schmidt, S. Codlin, W. Chen, R. Aebersold and J. Bähler (2012). "Quantitative analysis of fission yeast transcriptomes and proteomes in proliferating and quiescent cells." Cell **151**(3): 671-683.

Markowska, A., P. Rebuffat, G. Gottardo, G. Mazzochi and G. G. Nussdorfer (1994). "Age-dependent changes in the function and morphology of mitochondria of rat adrenal zona fasciculata." Histol Histopathol **9**(2): 263-268.

Milo, R. (2013). "What is the total number of protein molecules per cell volume? A call to rethink some published values." Bioessays **35**(12): 1050-1055.

Montano, S. J., J. Lu, T. N. Gustafsson and A. Holmgren (2014). "Activity assays of mammalian thioredoxin and thioredoxin reductase: fluorescent disulfide substrates, mechanisms, and use with tissue samples." Anal Biochem **449**: 139-146.

Moore, R. B., M. V. Mankad, S. K. Shriver, V. N. Mankad and G. A. Plishker (1991). "Reconstitution of Ca (2+)-dependent K+ transport in erythrocyte membrane vesicles requires a cytoplasmic protein." Journal of Biological Chemistry **266**(28): 18964-18968.

Munro, D., S. Banh, E. Sotiri, N. Tamanna and J. R. Treberg (2016). "The thioredoxin and glutathione-dependent H2O2 consumption pathways in muscle mitochondria: Involvement in H2O2 metabolism and consequence to H2O2 efflux assays." Free Radical Biology and Medicine **96**: 334-346.

Nagy, P., A. Karton, A. Betz, A. V. Peskin, P. Pace, R. J. O'Reilly, M. B. Hampton, L. Radom and C. C. Winterbourn (2011). "Model for the exceptional reactivity of peroxiredoxins 2 and 3 with hydrogen peroxide a kinetic and computational study." Journal of Biological Chemistry **286**(20): 18048-18055.

Noh, Y. H., J. Y. Baek, W. Jeong, S. G. Rhee and T. S. Chang (2009). "Sulfiredoxin Translocation into Mitochondria Plays a Crucial Role in Reducing Hyperoxidized Peroxiredoxin III." J Biol Chem **284**(13): 8470-8477.

Orrico, F., A. C. Lopez, D. Saliwonczyk, C. Acosta, I. Rodriguez-Grecco, I. Mouro-Chanteloup, M. A. Ostuni, A. Denicola, L. Thomson and M. N. Möller (2022). "The permeability of human red blood cell membranes to hydrogen peroxide is independent of aquaporins." J Biol Chem **298**(1): 101503.

Oshino, N., D. Jamieson, T. Sugano and B. Chance (1975). "Optical measurement of the catalase-hydrogen peroxide intermediate (Compound I) in the liver of anaesthetized rats and its implication to hydrogen peroxide production in situ." Biochemical Journal **146**(1): 67-77.

Peskin, A. V., N. Dickerhof, R. A. Poynton, L. N. Paton, P. E. Pace, M. B. Hampton and C. C. Winterbourn (2013). "Hyperoxidation of peroxiredoxins 2 and 3: rate constants for the reactions of the sulfenic acid of the peroxidatic cysteine." Journal of Biological Chemistry **288**(20): 14170-14177.

Peskin, A. V., F. M. Low, L. N. Paton, G. J. Maghzal, M. B. Hampton and C. C. Winterbourn (2007). "The high reactivity of peroxiredoxin 2 with H2O2 is not reflected in its reaction with other oxidants and thiol reagents." Journal of Biological Chemistry **282**(16): 11885-11892.

Peskin, A. V., F. C. Meotti, L. F. de Souza, R. F. Anderson, C. C. Winterbourn and A. Salvador (2020). "Intra-dimer cooperativity between the active site cysteines during the oxidation of peroxiredoxin 2." Free Radical Biology and Medicine **158**: 115-125.

Peskin, A. V., F. C. Meotti, K. M. Kean, C. Göbl, A. S. Peixoto, P. E. Pace, C. R. Horne, S. G. Heath, J. M. Crowther, R. C. J. Dobson, P. A. Karplus and C. C. Winterbourn (2021). "Modifying the resolving cysteine affects the structure and hydrogen peroxide reactivity of peroxiredoxin 2." J Biol Chem **296**: 100494.

Portillo-Ledesma, S., L. M. Randall, D. Parsonage, J. Dalla Rizza, P. A. Karplus, L. B. Poole, A. Denicola and G. Ferrer-Sueta (2018). "Differential Kinetics of Two-Cysteine Peroxiredoxin Disulfide Formation Reveal a Novel Model for Peroxide Sensing." Biochemistry **57**(24): 3416-3424.

Portillo-Ledesma, S., F. Sardi, B. Manta, M. V. Tourn, A. Clippe, B. Knoops, B. Alvarez, E. L. Coitiño and G. Ferrer-Sueta (2014). "Deconstructing the catalytic efficiency of peroxiredoxin-5 peroxidatic cysteine." Biochemistry **53**(38): 6113-6125.

Poynton, R. A., A. V. Peskin, A. C. Haynes, W. T. Lowther, M. B. Hampton and C. C. Winterbourn (2016). "Kinetic analysis of structural influences on the susceptibility of peroxiredoxins 2 and 3 to hyperoxidation." Biochem J **473**(4): 411-421.

Roussel, X., G. Béchade, A. Kriznik, A. Van Dorsselaer, S. Sanglier-Cianferani, G. Branlant and S. Rahuel-Clermont (2008). "Evidence for the formation of a covalent thiosulfinate intermediate with peroxiredoxin in the catalytic mechanism of sulfiredoxin." J Biol Chem **283**(33): 22371-22382.

Roussel, X., G. Béchade, A. Kriznik, A. Van Dorsselaer, S. Sanglier-Cianferani, G. Branlant and S. Rahuel-Clermont (2008). "Evidence for the Formation of a Covalent Thiosulfinate Intermediate with Peroxiredoxin in the Catalytic Mechanism of Sulfiredoxin *." Journal of Biological Chemistry **283**(33): 22371-22382.

Roussel, X., S. Boukhenouna, S. Rahuel-Clermont and G. Branlant (2011). "The rate-limiting step of sulfiredoxin is associated with the transfer of the γ-phosphate of ATP to the sulfinic acid of overoxidized typical 2-Cys peroxiredoxins." FEBS Letters **585**(3): 574-578.

Selvaggio, G., P. Coelho and A. Salvador (2018). "Mapping the phenotypic repertoire of the cytoplasmic 2-Cys peroxiredoxin - Thioredoxin system. 1. Understanding commonalities and differences among cell types." Redox Biol **15**: 297-315.

Starkov, A. A., A. Y. Andreyev, S. F. Zhang, N. N. Starkova, M. Korneeva, M. Syromyatnikov and V. N. Popov (2014). "Scavenging of H2O2 by mouse brain mitochondria." Journal of Bioenergetics and Biomembranes **46**(6): 471-477.

Stein, K. T., S. J. Moon, A. N. Nguyen and H. D. Sikes (2020). "Kinetic modeling of H2O2 dynamics in the mitochondria of HeLa cells." PLoS Comput Biol **16**(9): e1008202.

Stone, J. R. and S. Yang (2006). "Hydrogen peroxide: a signaling messenger." Antioxid Redox Signal **8**(3-4): 243-270.

Tomalin, L. E., A. M. Day, Z. E. Underwood, G. R. Smith, P. Dalle Pezze, C. Rallis, W. Patel, B. C. Dickinson, J. Bähler and T. F. Brewer (2016). "Increasing extracellular H2O2 produces a bi-phasic response in intracellular H2O2, with peroxiredoxin hyperoxidation only triggered once the cellular H2O2-buffering capacity is overwhelmed." Free Radical Biology and Medicine **95**: 333-348.

Travasso, R. D., F. S. dos Aidos, A. Bayani, P. Abranches and A. Salvador (2017). "Localized Redox Relays as a Privileged Mode of Cytoplasmic Hydrogen Peroxide Signaling." Redox Biology.

Treberg, J. R., K. Braun and P. Selseleh (2019). "Mitochondria can act as energy-sensing regulators of hydrogen peroxide availability." Redox Biology **20**: 483-488.

Treberg, J. R., D. Munro, S. Banh, P. Zacharias and E. Sotiri (2015). "Differentiating between apparent and actual rates of H2O2 metabolism by isolated rat muscle mitochondria to test a simple model of mitochondria as regulators of H2O2 concentration." Redox Biol **5**: 216-224.

Trujillo, M., A. Clippe, B. Manta, G. Ferrer-Sueta, A. Smeets, J.-P. Declercq, B. Knoops and R. Radi (2007).

"Pre-steady state kinetic characterization of human peroxiredoxin 5: taking advantage of Trp84 fluorescence increase upon oxidation." Archives of biochemistry and biophysics **467**(1): 95-106.

Turanov, A. A., D. Su and V. N. Gladyshev (2006). "Characterization of alternative cytosolic forms and cellular targets of mouse mitochondrial thioredoxin reductase." J Biol Chem **281**(32): 22953-22963.

Vazquez, A. (2018). Chapter 2 - Biochemical Horsepower. Overflow Metabolism. A. Vazquez, Academic Press**:** 7-14.

Villar, S. F., L. Corrales-González, B. Márquez de los Santos, J. Dalla Rizza, A. Zeida, A. Denicola and G. Ferrer-Sueta (2023). "Kinetic and structural assessment of the reduction of human 2-Cys peroxiredoxins by thioredoxins." The FEBS Journal.

Wang, M., C. J. Herrmann, M. Simonovic, D. Szklarczyk and C. Mering (2015). "Version 4.0 of PaxDb: protein abundance data, integrated across model organisms, tissues, and cell - lines." Proteomics **15**(18): 3163-3168.

Wang, X., L. Wang, X. Wang, F. Sun and C. C. Wang (2012). "Structural insights into the peroxidase activity and inactivation of human peroxiredoxin 4." Biochem J **441**(1): 113-118.

Wei, Q., H. Jiang, Z. Xiao, A. Baker, M. R. Young, T. D. Veenstra and N. H. Colburn (2011). "Sulfiredoxin–peroxiredoxin IV axis promotes human lung cancer progression through modulation of specific phosphokinase signaling." Proceedings of the National Academy of Sciences **108**(17): 7004-7009.

Winterbourn, C. C. and M. B. Hampton (2008). "Thiol chemistry and specificity in redox signaling." Free Radical Biology and Medicine **45**(5): 549-561.

Wiśniewski, J. R., A. Vildhede, A. Norén and P. Artursson (2016). "In-depth quantitative analysis and comparison of the human hepatocyte and hepatoma cell line HepG2 proteomes." Journal of Proteomics **136**: 234-247.

Woo, H. A., S. H. Yim, D. H. Shin, D. Kang, D.-Y. Yu and S. G. Rhee (2010). "Inactivation of peroxiredoxin I by phosphorylation allows localized H 2 O 2 accumulation for cell signaling." Cell **140**(4): 517-528.

Yang, K. S., S. W. Kang, H. A. Woo, S. C. Hwang, H. Z. Chae, K. Kim and S. G. Rhee (2002). "Inactivation of human peroxiredoxin I during catalysis as the result of the oxidation of the catalytic site cysteine to cysteine-sulfinic acid." J Biol Chem **277**(41): 38029-38036.

Zhong, L., E. S. Arnér and A. Holmgren (2000). "Structure and mechanism of mammalian thioredoxin reductase: the active site is a redox-active selenolthiol/selenenylsulfide formed from the conserved cysteine-selenocysteine sequence." Proc Natl Acad Sci U S A **97**(11): 5854-5859.
Adimora, N. J., D. P. Jones and M. L. Kemp (2010). "A model of redox kinetics implicates the thiol proteome in cellular hydrogen peroxide responses." Antioxidants & redox signaling **13**(6): 731-743.

Antunes, F. and E. Cadenas (2000). "Estimation of H2O2 gradients across biomembranes." FEBS Lett **475**(2): 121-126.

Antunes, F. and E. Cadenas (2001). "Cellular titration of apoptosis with steady state concentrations of H 2 O 2: submicromolar levels of H 2 O 2 induce apoptosis through Fenton chemistry independent of the cellular thiol state." Free Radical Biology and Medicine **30**(9): 1008-1018.

Barth, E., G. Stämmler, B. Speiser and J. Schaper (1992). "Ultrastructural quantitation of mitochondria and myofilaments in cardiac muscle from 10 different animal species including man." Journal of Molecular and Cellular Cardiology **24**(7): 669-681.

Benfeitas, R., G. Selvaggio, F. Antunes, P. M. Coelho and A. Salvador (2014). "Hydrogen peroxide metabolism and sensing in human erythrocytes: a validated kinetic model and reappraisal of the role of peroxiredoxin II." Free Radical Biology and Medicine **74**: 35-49.

Benfeitas, R. M. V. (2011). The physiological role of peroxiredoxin 2 in human erythrocytes: a kinetic analysis.

Boukhenouna, S., H. Mazon, G. Branlant, C. Jacob, M. B. Toledano and S. Rahuel-Clermont (2015). "Evidence That Glutathione and the Glutathione System Efficiently Recycle 1-Cys Sulfiredoxin In Vivo." Antioxidants & Redox Signaling **22**(9): 731-743.

Boveris, A. and B. Chance (1973). "The mitochondrial generation of hydrogen peroxide. General properties and effect of hyperbaric oxygen." Biochemical Journal **134**(3): 707-716.

Boveris, A., N. Oshino and B. Chance (1972). "The cellular production of hydrogen peroxide." Biochemical Journal **128**(3): 617-630.

Carvalho, L. A. C., D. R. Truzzi, T. S. Fallani, S. V. Alves, J. C. Toledo, Jr., O. Augusto, L. E. S. Netto and F. C. Meotti (2017). "Urate hydroperoxide oxidizes human peroxiredoxin 1 and peroxiredoxin 2." J Biol Chem **292**(21): 8705-8715.

Chae, H. Z., H. J. Kim, S. W. Kang and S. G. Rhee (1999). "Characterization of three isoforms of mammalian peroxiredoxin that reduce peroxides in the presence of thioredoxin." Diabetes Res Clin Pract **45**(2-3): 101-112.

Chang, T.-S., W. Jeong, H. A. Woo, S. M. Lee, S. Park and S. G. Rhee (2004). "Characterization of mammalian sulfiredoxin and its reactivation of hyperoxidized peroxiredoxin through reduction of cysteine sulfinic acid in the active site to cysteine." Journal of Biological Chemistry **279**(49): 50994-51001.

Cox, A. G., A. G. Pearson, J. M. Pullar, T. J. Jönsson, W. T. Lowther, C. C. Winterbourn and M. B. Hampton (2009). "Mitochondrial peroxiredoxin 3 is more resilient to hyperoxidation than cytoplasmic peroxiredoxins." Biochemical Journal **421**(1): 51-58.

Cox, A. G., A. V. Peskin, L. N. Paton, C. C. Winterbourn and M. B. Hampton (2009). "Redox potential and peroxide reactivity of human peroxiredoxin 3." Biochemistry **48**(27): 6495-6501.

Dalla Rizza, J., L. M. Randall, J. Santos, G. Ferrer-Sueta and A. Denicola (2019). "Differential parameters between cytosolic 2-Cys peroxiredoxins, PRDX1 and PRDX2." Protein Science **28**(1): 191-201.

del Olmo, M., A. Kramer and H. Herzel (2019). "A Robust Model for Circadian Redox Oscillations." Int J Mol Sci **20**(9).

Drechsel, D. A. and M. Patel (2010). "Respiration-dependent H2O2 removal in brain mitochondria via the thioredoxin/peroxiredoxin system." J Biol Chem **285**(36): 27850-27858.

Gauthier, L. D., J. L. Greenstein, B. O'Rourke and R. L. Winslow (2013). "An integrated mitochondrial ROS production and scavenging model: implications for heart failure." Biophys J **105**(12): 2832-2842.

Geiger, T., A. Wehner, C. Schaab, J. Cox and M. Mann (2012). "Comparative proteomic analysis of eleven common cell lines reveals ubiquitous but varying expression of most proteins." Mol Cell Proteomics **11**(3): M111.014050.

Gerencser, A. A., C. Chinopoulos, M. J. Birket, M. Jastroch, C. Vitelli, D. G. Nicholls and M. D. Brand (2012). "Quantitative measurement of mitochondrial membrane potential in cultured cells: calcium-induced de- and hyperpolarization of neuronal mitochondria." J Physiol **590**(12): 2845-2871.

Ghaemmaghami, S., W.-K. Huh, K. Bower, R. W. Howson, A. Belle, N. Dephoure, E. K. O'Shea and J. S. Weissman (2003). "Global analysis of protein expression in yeast." Nature **425**(6959): 737-741.

Griffith, M., A. Araújo, R. Travasso and A. Salvador (2024). "The architecture of redox microdomains: Cascading gradients and peroxiredoxins' redox-oligomeric coupling integrate redox signaling and antioxidant protection." Redox Biology **69**: 103000.

Gromer, S., L. D. Arscott, C. H. Williams, Jr., R. H. Schirmer and K. Becker (1998). "Human placenta thioredoxin reductase. Isolation of the selenoenzyme, steady state kinetics, and inhibition by therapeutic gold compounds." J Biol Chem **273**(32): 20096-20101.

Holmgren, A. and M. Luthman (1978). "Tissue distribution and subcellular localization of bovine thioredoxin determined by radioimmunoassay." Biochemistry **17**(19): 4071-4077.

Jeong, W., S. J. Park, T. S. Chang, D. Y. Lee and S. G. Rhee (2006). "Molecular mechanism of the reduction of cysteine sulfinic acid of peroxiredoxin to cysteine by mammalian sulfiredoxin." J Biol Chem **281**(20): 14400-14407.

Jones, D. P. (2008). "Radical-free biology of oxidative stress." American Journal of Physiology-Cell Physiology **295**(4): C849-C868.

Kembro, J. M., M. A. Aon, R. L. Winslow, B. O'Rourke and S. Cortassa (2013). "Integrating mitochondrial energetics, redox and ROS metabolic networks: a two-compartment model." Biophys J **104**(2): 332-343.

Kil, I. S., K. W. Ryu, S. K. Lee, J. Y. Kim, S. Y. Chu, J. H. Kim, S. Park and S. G. Rhee (2015). "Circadian oscillation of sulfiredoxin in the mitochondria." Molecular cell **59**(4): 651-663.

Kim, H., Y. Jung, B. S. Shin, H. Kim, H. Song, S. H. Bae, S. G. Rhee and W. Jeong (2010). "Redox regulation of lipopolysaccharide-mediated sulfiredoxin induction, which depends on both AP-1 and Nrf2." J Biol Chem **285**(45): 34419-34428.

Komalapriya, C., D. Kaloriti, A. T. Tillmann, Z. Yin, C. Herrero-de-Dios, M. D. Jacobsen, R. C. Belmonte, G. Cameron, K. Haynes and C. Grebogi (2015). "Integrative model of oxidative stress adaptation in the fungal pathogen Candida albicans." PloS one **10**(9): e0137750.

Liebermeister, W., E. Noor, A. Flamholz, D. Davidi, J. Bernhardt and R. Milo (2014). "Visual account of protein investment in cellular functions." Proc Natl Acad Sci U S A **111**(23): 8488-8493.

Manta, B., M. Hugo, C. Ortiz, G. Ferrer-Sueta, M. Trujillo and A. Denicola (2009). "The peroxidase and peroxynitrite reductase activity of human erythrocyte peroxiredoxin 2." Archives of biochemistry and biophysics **484**(2): 146-154.

Marguerat, S., A. Schmidt, S. Codlin, W. Chen, R. Aebersold and J. Bähler (2012). "Quantitative analysis of fission yeast transcriptomes and proteomes in proliferating and quiescent cells." Cell **151**(3): 671-683.

Markowska, A., P. Rebuffat, G. Gottardo, G. Mazzochi and G. G. Nussdorfer (1994). "Age-dependent changes in the function and morphology of mitochondria of rat adrenal zona fasciculata." Histol Histopathol **9**(2): 263-268.

Milo, R. (2013). "What is the total number of protein molecules per cell volume? A call to rethink some published values." Bioessays **35**(12): 1050-1055.

Montano, S. J., J. Lu, T. N. Gustafsson and A. Holmgren (2014). "Activity assays of mammalian thioredoxin and thioredoxin reductase: fluorescent disulfide substrates, mechanisms, and use with tissue samples." Anal Biochem **449**: 139-146.

Moore, R. B., M. V. Mankad, S. K. Shriver, V. N. Mankad and G. A. Plishker (1991). "Reconstitution of Ca (2+)-dependent K+ transport in erythrocyte membrane vesicles requires a cytoplasmic protein." Journal of Biological Chemistry **266**(28): 18964-18968.

Munro, D., S. Banh, E. Sotiri, N. Tamanna and J. R. Treberg (2016). "The thioredoxin and glutathione-dependent H2O2 consumption pathways in muscle mitochondria: Involvement in H2O2 metabolism and consequence to H2O2 efflux assays." Free Radical Biology and Medicine **96**: 334-346.

Nagy, P., A. Karton, A. Betz, A. V. Peskin, P. Pace, R. J. O'Reilly, M. B. Hampton, L. Radom and C. C. Winterbourn (2011). "Model for the exceptional reactivity of peroxiredoxins 2 and 3 with hydrogen peroxide a kinetic and computational study." Journal of Biological Chemistry **286**(20): 18048-18055.

Noh, Y. H., J. Y. Baek, W. Jeong, S. G. Rhee and T. S. Chang (2009). "Sulfiredoxin Translocation into Mitochondria Plays a Crucial Role in Reducing Hyperoxidized Peroxiredoxin III." J Biol Chem **284**(13): 8470-8477.

Orrico, F., A. C. Lopez, D. Saliwonczyk, C. Acosta, I. Rodriguez-Grecco, I. Mouro-Chanteloup, M. A. Ostuni, A. Denicola, L. Thomson and M. N. Möller (2022). "The permeability of human red blood cell membranes to hydrogen peroxide is independent of aquaporins." J Biol Chem **298**(1): 101503.

Oshino, N., D. Jamieson, T. Sugano and B. Chance (1975). "Optical measurement of the catalase-hydrogen peroxide intermediate (Compound I) in the liver of anaesthetized rats and its implication to hydrogen peroxide production in situ." Biochemical Journal **146**(1): 67-77.

Peskin, A. V., N. Dickerhof, R. A. Poynton, L. N. Paton, P. E. Pace, M. B. Hampton and C. C. Winterbourn (2013). "Hyperoxidation of peroxiredoxins 2 and 3: rate constants for the reactions of the sulfenic acid of the peroxidatic cysteine." Journal of Biological Chemistry **288**(20): 14170-14177.

Peskin, A. V., F. M. Low, L. N. Paton, G. J. Maghzal, M. B. Hampton and C. C. Winterbourn (2007). "The high reactivity of peroxiredoxin 2 with H2O2 is not reflected in its reaction with other oxidants and thiol reagents." Journal of Biological Chemistry **282**(16): 11885-11892.

Peskin, A. V., F. C. Meotti, L. F. de Souza, R. F. Anderson, C. C. Winterbourn and A. Salvador (2020). "Intra-dimer cooperativity between the active site cysteines during the oxidation of peroxiredoxin 2." Free Radical Biology and Medicine **158**: 115-125.

Peskin, A. V., F. C. Meotti, K. M. Kean, C. Göbl, A. S. Peixoto, P. E. Pace, C. R. Horne, S. G. Heath, J. M. Crowther, R. C. J. Dobson, P. A. Karplus and C. C. Winterbourn (2021). "Modifying the resolving cysteine affects the structure and hydrogen peroxide reactivity of peroxiredoxin 2." J Biol Chem **296**: 100494.

Portillo-Ledesma, S., L. M. Randall, D. Parsonage, J. Dalla Rizza, P. A. Karplus, L. B. Poole, A. Denicola and G. Ferrer-Sueta (2018). "Differential Kinetics of Two-Cysteine Peroxiredoxin Disulfide Formation Reveal a Novel Model for Peroxide Sensing." Biochemistry **57**(24): 3416-3424.

Portillo-Ledesma, S., F. Sardi, B. Manta, M. V. Tourn, A. Clippe, B. Knoops, B. Alvarez, E. L. Coitiño and G. Ferrer-Sueta (2014). "Deconstructing the catalytic efficiency of peroxiredoxin-5 peroxidatic cysteine." Biochemistry **53**(38): 6113-6125.

Poynton, R. A., A. V. Peskin, A. C. Haynes, W. T. Lowther, M. B. Hampton and C. C. Winterbourn (2016). "Kinetic analysis of structural influences on the susceptibility of peroxiredoxins 2 and 3 to hyperoxidation." Biochem J **473**(4): 411-421.

Roussel, X., G. Béchade, A. Kriznik, A. Van Dorsselaer, S. Sanglier-Cianferani, G. Branlant and S. Rahuel-Clermont (2008). "Evidence for the formation of a covalent thiosulfinate intermediate with peroxiredoxin in

the catalytic mechanism of sulfiredoxin." J Biol Chem **283**(33): 22371-22382.

Roussel, X., G. Béchade, A. Kriznik, A. Van Dorsselaer, S. Sanglier-Cianferani, G. Branlant and S. Rahuel-Clermont (2008). "Evidence for the Formation of a Covalent Thiosulfinate Intermediate with Peroxiredoxin in the Catalytic Mechanism of Sulfiredoxin *." Journal of Biological Chemistry **283**(33): 22371-22382.

Roussel, X., S. Boukhenouna, S. Rahuel-Clermont and G. Branlant (2011). "The rate-limiting step of sulfiredoxin is associated with the transfer of the γ-phosphate of ATP to the sulfinic acid of overoxidized typical 2-Cys peroxiredoxins." FEBS Letters **585**(3): 574-578.

Selvaggio, G., P. Coelho and A. Salvador (2018). "Mapping the phenotypic repertoire of the cytoplasmic 2-Cys peroxiredoxin - Thioredoxin system. 1. Understanding commonalities and differences among cell types." Redox Biol **15**: 297-315.

Starkov, A. A., A. Y. Andreyev, S. F. Zhang, N. N. Starkova, M. Korneeva, M. Syromyatnikov and V. N. Popov (2014). "Scavenging of H2O2 by mouse brain mitochondria." Journal of Bioenergetics and Biomembranes **46**(6): 471-477.

Stein, K. T., S. J. Moon, A. N. Nguyen and H. D. Sikes (2020). "Kinetic modeling of H2O2 dynamics in the mitochondria of HeLa cells." PLoS Comput Biol **16**(9): e1008202.

Stone, J. R. and S. Yang (2006). "Hydrogen peroxide: a signaling messenger." Antioxid Redox Signal **8**(3-4): 243-270.

Tomalin, L. E., A. M. Day, Z. E. Underwood, G. R. Smith, P. Dalle Pezze, C. Rallis, W. Patel, B. C. Dickinson, J. Bähler and T. F. Brewer (2016). "Increasing extracellular H2O2 produces a bi-phasic response in intracellular H2O2, with peroxiredoxin hyperoxidation only triggered once the cellular H2O2-buffering capacity is overwhelmed." Free Radical Biology and Medicine **95**: 333-348.

Travasso, R. D., F. S. dos Aidos, A. Bayani, P. Abranches and A. Salvador (2017). "Localized Redox Relays as a Privileged Mode of Cytoplasmic Hydrogen Peroxide Signaling." Redox Biology.

Treberg, J. R., K. Braun and P. Selseleh (2019). "Mitochondria can act as energy-sensing regulators of hydrogen peroxide availability." Redox Biology **20**: 483-488.

Treberg, J. R., D. Munro, S. Banh, P. Zacharias and E. Sotiri (2015). "Differentiating between apparent and actual rates of H2O2 metabolism by isolated rat muscle mitochondria to test a simple model of mitochondria as regulators of H2O2 concentration." Redox Biol **5**: 216-224.

Trujillo, M., A. Clippe, B. Manta, G. Ferrer-Sueta, A. Smeets, J.-P. Declercq, B. Knoops and R. Radi (2007). "Pre-steady state kinetic characterization of human peroxiredoxin 5: taking advantage of Trp84 fluorescence increase upon oxidation." Archives of biochemistry and biophysics **467**(1): 95-106.

Turanov, A. A., D. Su and V. N. Gladyshev (2006). "Characterization of alternative cytosolic forms and cellular targets of mouse mitochondrial thioredoxin reductase." J Biol Chem **281**(32): 22953-22963.

Vazquez, A. (2018). Chapter 2 - Biochemical Horsepower. Overflow Metabolism. A. Vazquez, Academic Press**:** 7-14.

Villar, S. F., L. Corrales-González, B. Márquez de los Santos, J. Dalla Rizza, A. Zeida, A. Denicola and G. Ferrer-Sueta (2023). "Kinetic and structural assessment of the reduction of human 2-Cys peroxiredoxins by thioredoxins." The FEBS Journal.

Wang, M., C. J. Herrmann, M. Simonovic, D. Szklarczyk and C. Mering (2015). "Version 4.0 of PaxDb:

protein abundance data, integrated across model organisms, tissues, and cell‐lines." Proteomics **15**(18): 3163-3168.

Wang, X., L. Wang, X. Wang, F. Sun and C. C. Wang (2012). "Structural insights into the peroxidase activity and inactivation of human peroxiredoxin 4." Biochem J **441**(1): 113-118.

Wei, Q., H. Jiang, Z. Xiao, A. Baker, M. R. Young, T. D. Veenstra and N. H. Colburn (2011). "Sulfiredoxin–peroxiredoxin IV axis promotes human lung cancer progression through modulation of specific phosphokinase signaling." Proceedings of the National Academy of Sciences **108**(17): 7004-7009.

Winterbourn, C. C. and M. B. Hampton (2008). "Thiol chemistry and specificity in redox signaling." Free Radical Biology and Medicine **45**(5): 549-561.

Wiśniewski, J. R., A. Vildhede, A. Norén and P. Artursson (2016). "In-depth quantitative analysis and comparison of the human hepatocyte and hepatoma cell line HepG2 proteomes." Journal of Proteomics **136**: 234-247.

Woo, H. A., S. H. Yim, D. H. Shin, D. Kang, D.-Y. Yu and S. G. Rhee (2010). "Inactivation of peroxiredoxin I by phosphorylation allows localized H 2 O 2 accumulation for cell signaling." Cell **140**(4): 517-528.

Yang, K. S., S. W. Kang, H. A. Woo, S. C. Hwang, H. Z. Chae, K. Kim and S. G. Rhee (2002). "Inactivation of human peroxiredoxin I during catalysis as the result of the oxidation of the catalytic site cysteine to cysteine-sulfinic acid." J Biol Chem **277**(41): 38029-38036.

Zhong, L., E. S. Arnér and A. Holmgren (2000). "Structure and mechanism of mammalian thioredoxin reductase: the active site is a redox-active selenolthiol/selenenylsulfide formed from the conserved cysteine-selenocysteine sequence." Proc Natl Acad Sci U S A **97**(11): 5854-5859.
